# Decoding the Ambiguous Electron Paramagnetic Resonance Signals in the Lytic Polysaccharide Monooxygenase from *Photorhabdus luminescens*

**DOI:** 10.1021/acs.inorgchem.2c00766

**Published:** 2022-05-12

**Authors:** Rogelio J. Gómez-Piñeiro, Maria Drosou, Sylvain Bertaina, Christophe Decroos, A. Jalila Simaan, Dimitrios A. Pantazis, Maylis Orio

**Affiliations:** † Aix Marseille Université, CNRS, Centrale Marseille, iSm2, Marseille 13397, France; ‡ Inorganic Chemistry Laboratory, National and Kapodistrian University of Athens, Panepistimiopolis, Zografou 15771, Greece; § Aix-Marseille Université, CNRS, IM2NP UMR 7334, Marseille 13397, France; ∥ Max-Planck-Institut für Kohlenforschung, Kaiser-Wilhelm-Platz 1, Mülheim an der Ruhr 45470, Germany

## Abstract

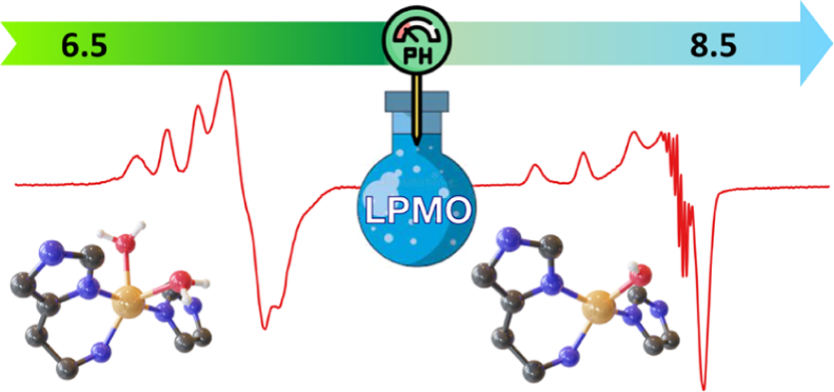

Understanding the structure and function of lytic polysaccharide monooxygenases (LPMOs), copper enzymes that degrade recalcitrant polysaccharides, requires the reliable atomistic interpretation of electron paramagnetic resonance (EPR) data on the Cu(II) active site. Among various LPMO families, the chitin-active *Pl*AA10 shows an intriguing phenomenology with distinct EPR signals, a major rhombic and a minor axial signal. Here, we combine experimental and computational investigations to uncover the structural identity of these signals. X-band EPR spectra recorded at different pH values demonstrate pH-dependent population inversion: the major rhombic signal at pH 6.5 becomes minor at pH 8.5, where the axial signal dominates. This suggests that a protonation change is involved in the interconversion. Precise structural interpretations are pursued with quantum chemical calculations. Given that accurate calculations of Cu *g*-tensors remain challenging for quantum chemistry, we first address this problem via a thorough calibration study. This enables us to define a density functional that achieves accurate and reliable prediction of *g*-tensors, giving confidence in our evaluation of *Pl*AA10 LPMO models. Large models were considered that include all parts of the protein matrix surrounding the Cu site, along with the characteristic second-sphere features of *Pl*AA10. The results uniquely identify the rhombic signal with a five-coordinate Cu ion bearing two water molecules in addition to three N-donor ligands. The axial signal is attributed to a four-coordinate Cu ion where only one of the waters remains bound, as hydroxy. Alternatives that involve decoordination of the histidine brace amino group are unlikely based on energetics and spectroscopy. These results provide a reliable spectroscopy-consistent view on the plasticity of the resting state in *Pl*AA10 LPMO as a foundation for further elucidating structure–property relationships and the formation of catalytically competent species. Our strategy is generally applicable to the study of EPR parameters of mononuclear copper-containing metalloenzymes.

## Abstract 2

Accurate quantum chemical protocols for the calculation of Cu(II) electron paramagnetic resonance parameters combined with experimental spectroscopic results on the lytic polysaccharide monooxygenase *Pl*AA10 enable the reliable active-site structural assignment of signals observed at different pH values.

## 1 Introduction

**Figure 1:**
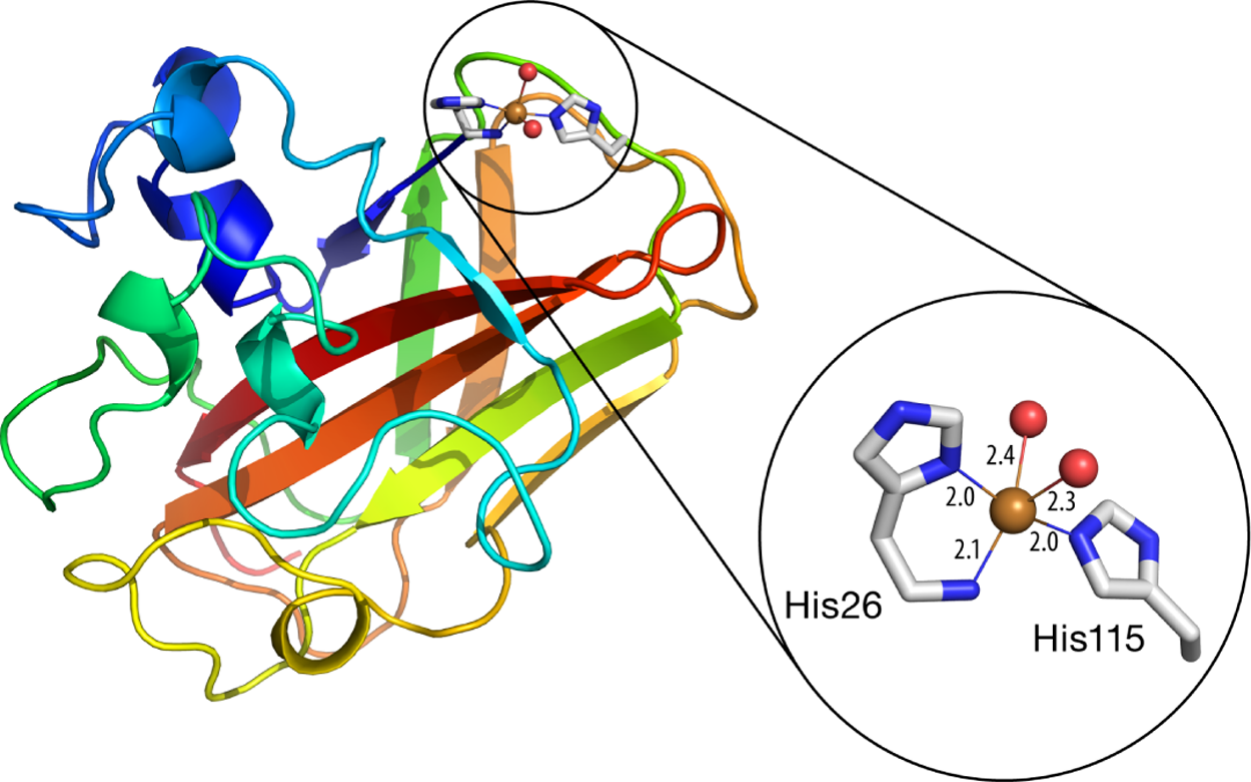
Structure of the *Pl*AA10 LPMO enzyme (PDB code: 6T5Z)^15^ and of its active site, with special focus on the copper center and its first coordination sphere. Bond distances shown in Å.

Lytic polysaccharide monooxygenase (LPMO) enzymes are intensely studied for their ability to oxidatively cleave the glycosidic bond of recalcitrant polysaccharides such as cellulose^1−3^ and chitin.^4,5^ Given the societal impulse to find new sustainable alternatives to meet global energy demands, there is great interest to apply the LPMO reactivity in the production of second-generation biofuels from biomass.^6,7^ Understanding the structure–function properties of the LPMO active site will give great insights into the development of future biomimetic models.^8^ The active site of LPMOs is composed of a mononuclear copper center coordinated by the N-terminal histidine amino acid in a bidentate fashion and a second histidine side chain to form a motif known as histidine brace. Two water molecules complete the first coordination sphere of the Cu(II) metal center (Figure 1).^1,4^ This coordination motif is very rare and is only observed in a few copper-containing metalloproteins such as the PmoB subunit of another enigmatic enzyme, the particulate methane monooxygenase,^9^ CopC^10,11^ and PmoF,^12^ two bacterial copper-transport proteins, and LPMO-like fungal copper-transport proteins.^13,14^

In the enzyme resting state, the copper center is at the +II oxidation state (3d^9^ configuration), leading to an *S* = 1/2 spin state.^1^ To investigate copper-containing systems, electron paramagnetic resonance (EPR) spectroscopy is the technique of choice as it enables probing the electronic and geometric configuration of the paramagnetic metal center with the information encoded in the *g* and hyperfine coupling, *A*, tensors.^16,17^ Despite the presence of a well conserved Cu site in the various LPMO enzymes, their EPR parameters are quite diverse,^6,18^ often complicating the reliable structural interpretation of EPR signals. In the chitin-active LPMO from *Photorhabdus luminescens*, named *Pl*AA10, and produced in our group, two co-existing species were detected by X-band EPR spectroscopy at 120 K using a frozen solution of *Pl*AA10 in MES [2-(*N*-morpholino)ethanesulfonic acid] buffer at pH 6.5.^15^ The first major species displayed the expected rhombic EPR signature (*g*_*z*_ ≠ *g*_*y*_ ≠ *g*_*x*_, *A*_*z*_^Cu^ ≠ *A*_*y*_^Cu^ ≠ A*_x_*^Cu^), in agreement with the EPR signature of other chitin-active LPMOs.^5,19−21^ This rhombicity is indicative of a distorted geometry falling between square base pyramidal and trigonal bipyramidal. On the contrary, the second, minor, species had distinct axial features (*g*_*z*_ > *g*_*y*_ ≈ *g*_*x*_, *A*_*z*_^Cu^ > *A*_*y*_^Cu^ ≈ A*_x_*^Cu^). By EPR simulations, the major/minor species distribution was fitted to 80% rhombic and 20% axial.

**Scheme 1:**
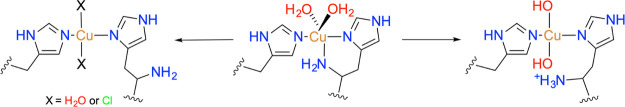
Proposed Copper Coordination in *Sli*LPMO10E (left) and in *Bl*AA10^21−23^

Another chitin-active LPMO, *Sli*LPMO10E from *Streptomyces lividans*, showed similar EPR characteristics.^21^ The copper bound protein in a Tris [tris(hydroxymethyl)aminomethane] buffer at pH 7.0 containing NaCl exhibited two distinct EPR signals at 10 K: one of rhombic character and another of axial character. Chaplin et al. suggested that the origin of the minor species with axial character in the *Sli*LPMO10E mixture may be due to the decoordination of the primary amine of the N-terminal histidine.^21^ In addition, the mixed signal may originate not only from this decoordination alone but also from a possible coordination of chloride anions present in excess (150 mM NaCl) in the Tris buffer (Scheme 1). It was also suggested that because the heterogeneity was observed in the apo-protein, the two configurations of the N-terminal coordination arm reflect a natural flexibility of LPMO enzymes for copper loading. The same rationale, that is, decoordination of the primary amine of the histidine brace, was suggested for the formation of the minor species in the *Pl*AA10 enzyme mixture. This heterogeneous solution may form during freezing of the sample in the absence of a glassing agent, for example glycerol, before the EPR measurement.^15,18^

Recently, Lindley et al. reported the study of a chitin-active LPMO, *Bl*AA10 from *Bacillus licheniformis*, that was investigated in different pH conditions using EPR spectroscopic techniques and computations. Their work focused on the protonation states of the coordination sphere of the protein active site. They proposed the species observed at moderate basic pH to result from the deprotonation of the ligating water molecules and the partial decoordination of the −NH_2_ group from the histidine brace motif.^22^

Finally, Serra et al. investigated a new enzyme that belongs to the AA10 family, *Pp*AA10 from *Pseudomonas putida*, by the means of Fourier transform infrared spectroscopy and EPR spectroscopies. Probing various buffer conditions, they investigated the influence of the pH on the EPR signature of the active and observed the presence of two different contributions to the spectra, having a rhombic and axial character, respectively. For the latter, they suggest that its formation would likely result from either alteration of the ligand water being replaced by a chloride ligand from the buffer or deprotonated, or coordination of an extra residue to the copper center (Scheme 1).^23^

In light of these recent works, we seek to establish a robust methodology to elucidate the origin of the intriguing EPR signal distribution in the AA10 family using the *Pl*AA10 LPMO enzyme by building theoretical model systems of the active site and calculating their EPR parameters using benchmarked protocols based on density functional theory (DFT) methods. These models are constructed using the *Pl*AA10 crystal structure and considering numerous subsequent modifications around the copper center, such as decoordination of water molecules and of the primary amine, along with changes in protonation states of all relevant groups.

The pre-requisite of this approach is to be able to predict accurate EPR parameters from a theoretical model system. Therefore, the quantum chemical methods employed must be of proven reliability for both the *g* and *A* tensors.^24^ Our group has recently presented a highly accurate protocol for the calculation of Cu(II) hyperfine coupling constants based on a specific density functional and a customized flexible-core basis set.^25^ No method of similarly high accuracy has been established for the calculation of Cu(II) *g*-tensors;^26^ therefore, in the present study, we first undertake a thorough methodological evaluation using a large set of reference synthetic complexes. On this basis, we define a new modified functional that yields the most accurate *g*-tensor values compared to any other method reported in the literature, either DFT- or wave-function-based, and hence will serve as a reference method for any future study of Cu(II) systems. Applying our benchmarked protocols on various atomistic models of the *Pl*AA10 enzyme, we elucidate the chemical nature of the species resulting in the axial and rhombic EPR signals by comparing our computational results to the EPR measurements performed under different pH conditions. This work showcases the success of combining EPR spectroscopy with computational chemistry and contributes to a precise description of biologically relevant species in the family of AA10 LPMO enzymes.

## 2 Methodology

### 2.1 Experimental Details

Cu(II)-loaded *Pl*AA10 was produced as previously described.^15^ Protein samples (concentration around 200 μM) for continuous-wave EPR were prepared in either 50 mM MES buffer (pH 6.5) or 50 mM Tris·H_2_SO_4_ buffer (pH 8.5). EPR spectra were recorded on a Bruker Elexsys E500 spectrometer (Bruker, Karlsruhe, Germany) operating at X-band at 120 K (BVT 3000 digital temperature controller) with the following acquisition parameters: modulation frequency, 100 kHz; modulation amplitude, 5 G; conversion time, 90 ms; sweep time, 92.1 s; and microwave power, 20 mW. EPR spectra were simulated using the EasySpin toolbox developed for MATLAB.^27^ The optimum Hamiltonian parameters have been obtained using the second order perturbation, and then, an exact diagonalization has been used for the final simulations. Pseudo-modulation treatment of the spectra was performed to graphically extract the exact number of nitrogen centers contributing to the superhyperfine pattern.^28,29^*A*_N_ constants were considered isotropic and used in the final simulation of the first-derivative spectra. The Hamiltonian used for the simulations is the following equation
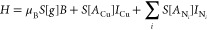
1with *i* = the number of nitrogen centers.

### 2.2 Computational Details

#### 2.2.1 Structural Models

For the methodological benchmarking part of this work, the coordinates of the 20 studied Cu complexes (Figure S1) were obtained from the Cambridge Structural Database (CSD) and were edited for completeness and chemical accuracy, for example, by removing solvent molecules or non-coordinating counterions and adding hydrogen atoms. The curated structures were subsequently employed in geometry optimizations. The set of compounds has been recently used in benchmarking quantum chemical methods for hyperfine coupling constants.^25^

**Figure 2:**
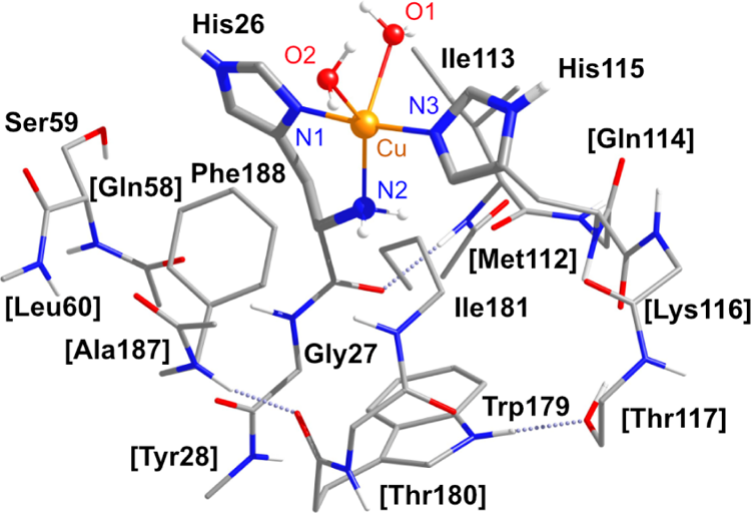
DFT model **A1** with the labels of the amino acids as defined in the 6T5Z X-ray structure of *Pl*AA10 LPMO.^15^ Hydrogen atoms bonded to carbon atoms are omitted for clarity. Truncated peripheral residues are denoted in square brackets.

Structural models of the *Pl*AA10 active site were constructed from the 6T5Z X-ray crystal structure with 1.6 Å resolution.^15^ The models include the amino acids that directly coordinate the Cu ion, His26 and His115, and second-sphere residues Ser59, Gly27, Ile113, Phe188, Trp179, and Ile181. Distinct interactions between the Cu coordination sphere and the protein envelope were conserved by also including fragments of the amino acids Tyr28, Gln58, Leu60, Met112, Gln114, Lys116, Thr117, Thr180, and Ala187. Two copper-coordinated crystallographic water molecules were included. Outer-layer crystallographically resolved solvent molecules were not included directly in the DFT models; long-range effects were accounted for through implicit solvation. The resulting model, denoted **A1** in the present work (Figure 2) has a five-coordinated Cu ion with two water ligands and consists of 197 atoms. All other structural modifications that were considered and evaluated in the present study were derived from this initial model.

#### 2.2.2 Geometry Optimizations

All calculations were performed with Orca quantum chemistry software.^30,31^ Geometry optimizations were carried out using the BP86 functional^32,33^ with the all-electron def2-TZVP basis sets.^34^ For the Coulomb fitting, the def2/J auxiliary basis sets were used.^35^ To investigate the dependence of the optimized geometry on the type of functional, geometry optimization of model **A1** was also performed with the hybrid functional B3LYP.^36,37^ Key structural parameters obtained with the BP86 and B3LYP functionals are compared in Table S1. Differences in Cu–ligand bond lengths of less than 0.03 Å and in N2–Cu–O2 and N1–Cu–N3 angles of less than 0.4° are observed, indicating negligible sensitivity of the calculated structural parameters on the choice of functional. The conductor-like polarizable continuum model with ε = 20, typically used to study enzymatic systems with active sites at the surface in buffer solutions,^38^ and the default refractive index of 1.33 was used in geometry optimizations of the enzyme models. Increased angular and radial integration grids (Grid4 and IntAcc 6.0, respectively, in Orca convention) and tight SCF convergence criteria were employed. Slow SCF convergence settings were applied in combination with recalculation of the full Fock matrix for each SCF step (DirectResetFreq 1) because this approach was found to ensure convergence even for uncommonly—electronically and computationally—difficult cases. In geometry optimizations of the *Pl*AA10 LPMO models, constraints were applied on selected backbone carbons and terminal hydrogen atoms (details provided in the Supporting Information) in order to maintain the structural effect of the protein matrix on the active site geometry.

#### 2.2.3 Calculations of EPR Parameters

Calculations of the copper hyperfine coupling constants and *g*-tensors employed increased general and radial integration grids (Grid6 and IntAcc 6.0), and specially enhanced grids for the copper center (SpecialGridIntAcc 11). The spin–orbit coupling operator was treated by an accurate mean-field (SOMF) approximation to the Breit–Pauli operator (SOCType 3).^39,40^ The potential was constructed to include one-electron terms, compute the Coulomb term in a semi-numeric way, incorporate exchange via one-center exact integrals including the spin–other orbit interaction, and include local DFT correlation (SOCFlags 1,2,3,1).

All EPR calculations used the previously defined aug-cc-pVTZ-Jmod basis set^25^ for Cu, while the def2-TZVP basis sets^34^ were used for all other atoms. The Cu basis set, a modified version of the property-optimized basis set originally proposed by Hedegård et al.,^41,42^ was shown to yield converged results for DFT calculations of hyperfine coupling constants.^25^ In contrast to the hyperfine coupling tensor that require a highly flexible and accurate description of the core region, the *g*-tensor is less sensitive to the choice of basis set and shows faster convergence, provided that the valence region is sufficiently well described. Even though a smaller and more standard basis set on Cu might be sufficient for *g*-tensor calculations, the large aug-cc-pVTZ-Jmod, which is flexible and accurate also in the valence space, is employed here for *g*-tensors as well because the computational overhead is acceptable, the results are converged (Table S2), and for maintaining consistency over all EPR property calculations.

*A*-tensors were computed with the B3PW91 functional,^36,43^ which was shown to be the best functional for copper hyperfine coupling constants in our recent evaluation study.^25^ An essential element of most modern DFT approximations is the admixture to the density functional of an adjustable amount of the electron exchange contribution from the Hartree–Fock theory, also known as “exact exchange” (the term exchange referring here to the quantum mechanical energy component arising from the antisymmetry requirement of the electronic wave function). For the calculation of *g*-tensors, we defined a modified version of B3PW91 with 40% exact (Hartree–Fock) exchange, following an extensive benchmarking of various DFT methods that is described in the first part of the present work. Other DFT functionals evaluated for the calculation of *g*-tensors include the standard generalized gradient approximation (GGA) functional PBE,^44^ the meta-GGA TPSS,^45^ the hybrids B3LYP,^36,37^ PBE0,^46^ and BHandHLYP, the hybrid-meta-GGA TPSSh,^47^ the long-range corrected or range separated functionals LC-BLYP^48^ and CAM-B3LYP,^49^ and the double-hybrid functionals B2PLYP,^50^ DSD-PBEP86,^51^ and PBE-QIDH.^52^ The RI approximation was used for the MP2 part in combination with a very large automatically generated correlation fitting auxiliary basis sets.^53^ The NoFrozenCore option and relaxed MP2 densities were used in the double-hybrid calculations. The exact exchange admixture was adjusted using the keywords ScalHFX and ScalDFX in Orca. Whenever we increased the contribution of exact exchange (ScalHFX), we decreased the default DFT exchange percentage by exactly the same amount. The use of scalar relativistic Hamiltonians, specifically the second-order Douglas–Kroll–Hess (DKH2)^54−60^ and the zeroth-order regular approximation,^61−63^ does not improve on average the quality of DFT calculations of *A*-tensors for copper systems.^25^ In the present work, we tested both Hamiltonians also for the calculation of *g*-tensors for a subset of the complexes (Table S3), applying picture change effects and recontracted versions^64^ of the def2 basis sets for the ligands. Similarly to the hyperfine coupling constants,^25^ an insignificant and not clearly beneficial effect was observed with the use of scalar relativistic Hamiltonians in the calculation of *g*-tensors; therefore, this approach was not adopted in the evaluation of the enzyme models.

## 3 Results and Discussion

### 3.1 DFT Methodology for Accurate Cu(II) *g*-Tensor Calculations

DFT has proven successful in the calculation of accurate hyperfine coupling constants for mononuclear copper systems. Previous benchmarking studies by Sciortino et al.^26^ and by us^25^ have identified density functionals that perform consistently well and with predictive accuracy. Moreover, the critical role of the basis set used for copper was investigated in detail and an optimal methodology has been proposed that combines the B3PW91 functional with the aug-cc-pVTZ-Jmod basis set.^25^ This approach was shown to consistently outperform applicable wave function methods for *A*-tensors.^25^ However, calculations of *g*-tensors for Cu systems present a more complex challenge.^65−67^ The evaluation study by Sciortino et al.^26^ documented the performance of representative functionals for *g*-tensors of several copper complexes, but the size and spread of errors suggest that no functional is a clearly and systematically superior choice. Remarkably, wave-function-based methods perform in general more poorly than DFT for Cu *g*-tensors,^68−71^ which underlines the difficulty of defining a robust and generally applicable theoretical protocol. In view of this problem, and given the need to have a reliable method for evaluating the many structurally different LPMO models, we begin this study by an extensive methodological benchmarking on a large reference set of spectroscopically characterized mononuclear Cu complexes. As described in the following, this enabled us to define a superior theoretical approach that gives strong confidence in the EPR parameters predicted for the LPMO models.

We employed the set of 20 mononuclear Cu(II) complexes depicted in Figure S1, as used also for the benchmarking of hyperfine coupling constants.^25^ These complexes have experimentally resolved *g*-tensors obtained from EPR measurements (Table 1). The set exhibits great variability in the properties of ligands: the coordinating atoms are of three different types (N, O, and S) and the coordination to the Cu center is in a 4N, 4S, 4O, 5N, or 6S fashion, or with a combination of atom types: 2N2O, 2N2S, or 3N1O. Some ligands are chelating, ranging from bidentate to tetradentate, and of varying sizes. The coordination geometry is also changing and includes square planar, tetrahedral, distorted square base pyramidal, and octahedral. The complexes also display a wide range of *g*_max_ values, between 2.085 and 2.285. We note that in this work, we adopt the convention of equating *g*_max_ with *g*_*z*_ in the case of computed values, but we maintain both nomenclatures for greater clarity in specific settings. The variety in coordination and properties provides us with a broad range to evaluate the performance of different computational methods.

**Table 1:** Experimental *g*-Values of the Complexes Considered in the Calibration Study^a^

	complex^b^	*g*_*x*_	*g*_*y*_	*g*_*z*_	refs.
**1**	[Cu(NH_3_)_4_]^2+^	2.047	2.047	2.241	(72)
**2**	[Cu(dtc)_2_]	2.025	2.025	2.085	(73)
**3**	[Cu(acac)^2^]	2.060	2.060	2.285	(74)
**4**	[Cu(en)_2_]^2+^	2.040	2.046	2.202	(75)
**5**	[Cu(mnt)_2_]^2–^	2.023	2.023	2.093	(76)
**6**	[Cu(gly)_2_]	2.052	2.052	2.267	(72)
**7**	[Cu(kts)]	2.030	2.030	2.140	(77)
**8**	[Cu(sac)_2_]	2.050	2.050	2.240	(78)
**9**	[Cu(im)_4_]^2+^	2.047	2.047	2.262	(72)
**10**	[Cu(py)_4_]^2+^	2.053	2.053	2.263	(72)
**11**	[Cu(eta)]^2+^	2.030	2.030	2.160	(79)
**12**	[Cu(epa)]^2+^	2.053	2.053	2.213	(80)
**13**	[Cu(atpt)]^2+^	2.100	2.100	2.235	(81)
**14**	[Cu(GGH)]^−^			2.173	(26)
**15**	[Cu(GGG)]^−^			2.202	(26)
**16**	[Cu(salpn)]	2.060	2.060	2.261	(82)
**17**	[Cu(*S*,*S*-mnpala)]	2.060	2.060	2.240	(83)
**18**	[Cu(salen)]	2.052	2.052	2.192	(84)
**19**	[Cu(bipy)_2_(NCS)]^+^	2.088	2.088	2.259	(85)
**20**	[Cu(ttcn)_2_]^2+^	2.045	2.045	2.117	(86)

a Complexes **14** and **15** were only considered for the prediction of the *g*_*z*_ component. Molecular structures of the complexes are depicted in Figure S1.

b Ligand abbreviations: dtc = dimethyldithiocarbamate, acac = acetylacetone; en = ethylenediamine; mnt = maleonitriledithiolate; gly = glycine; kts = 2-keto-3-ethoxybutyraldehyde-bis(thiosemicarbazone); sac = salicylaldehyde imine; im = imidazole; py = pyridine; epa = *N*,*N*′-ethylenebis(pyridine-2-aldimine); eta = *N*,*N*′-ethylenebis(thiophene-2-aldimine); atpt = 3,4-bis(3-amino-1-thiopropyl)toluene; GGH = glycine–glycine–histidine; GGG = glycine–glycine–glycine; salpn = *N*,*N*′-bis(salicylidene)-1,2-propanediamine; (*S*,*S*)-mnpala = 2,5,8-trimethyl-5-nitro-3,7-diazanonanedioate; salen = bis(salicylidene)ethylenediamine; bipy = 2,2′-bipyridine; and ttcn = 1,4,7-trithiacyclononane.

To evaluate the performance of the different functionals, the difference (*D*) between the calculated and experimental *g*-tensor components was used, defined as follows: *D*(*g*_*x*,*y*_) = *g*_*x*,*y*_^calc^ – *g*_*x*,*y*_^exp^ and *D*(*g*_*z*_) = gzcalc – *g*_*z*_^exp^, where *g*_*x*,*y*_ = *g*_⊥_ = (*g*_*x*_ + *g*_*y*_)/2 and *g*_*z*_ = *g*_∥_ is the maximum tensor component. In addition, the parameter Δ*g*, defined as the difference between the parallel and the perpendicular *g*-tensor components: Δ*g* = *g*_*z*_ – *g*_*x*,*y*_, is also used as a criterion of the performance of the different functionals according to the following: *D*(Δ*g*) = Δ*g*^calc^ – Δ*g*^exp^. For each examined functional, the mean difference (MD) between the calculated and experimental parameters defined above, is estimated as the average of the *D* values of the *N* = 20 Cu(II) complexes. For the case of *g*_*z*_, the equation is
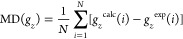
2where *i* runs over the Cu(II) complexes **1**–**20**. The mean per mille difference (‰ MPD) is the average of the per mille differences of complexes **1**–**20** for each functional and is given by
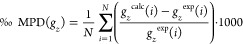
3

The calculation of the mean absolute per mille difference ‰ MAPD, by comparing to the ‰ MPD, enables us to identify possible systematic over- or underestimation of the *g-*tensor components by each functional. It is given by
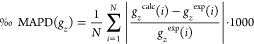
4

Finally, the mean percent difference (MPD) and mean absolute percent difference (MAPD) of the *g* shift of the *z* component (*g*_*z*_^s^), defined as the deviation from the free-electron value (*g*_e_ = 2.002319): *g*_*z*_^s^ = *g*_*z*_ – *g*_e_ are also presented.

**Figure 3:**
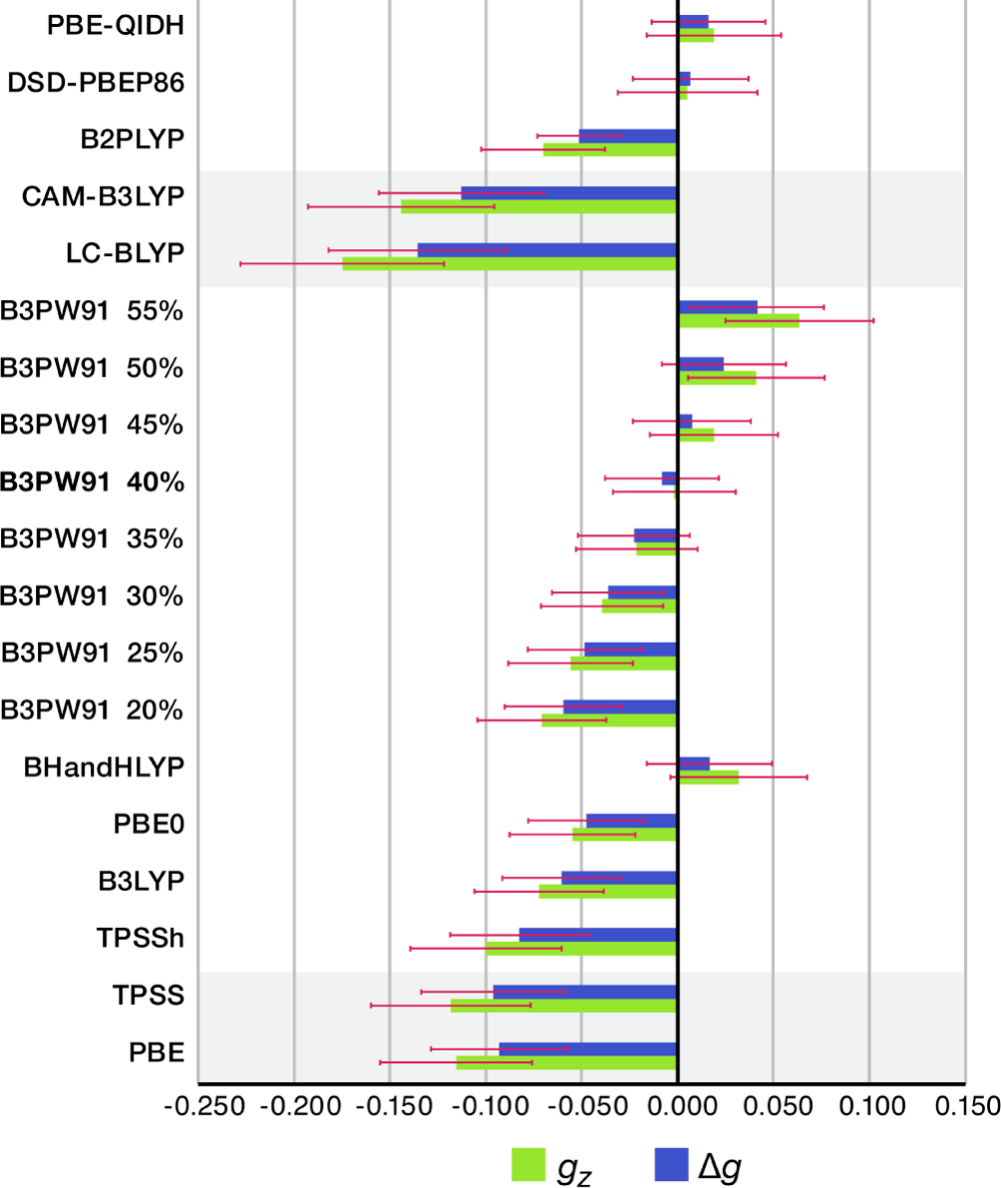
Comparison of the MDs of the calculated *g*_*z*_ and Δ*g* from the experimental values between different DFT functionals for the set of 20 Cu(II) complexes. The effect of variable exact exchange is shown in the series of results (from 20 to 55% exact exchange) with the B3PW91 functional. The red bars indicate standard deviations.

The performances of 19 functionals that belong to all different rungs of DFT in terms of the most important MD, ‰ MPD, and ‰ MAPD values for the set of Cu(II) complexes are compared in Table 2. The complete sets of computed values for all complexes and functionals are given in Tables S4–S22. We first observe that MD(*g*_*x*,*y*_) values are significantly smaller than MD(*g*_*z*_) values for all functionals, which means that the *g*_*z*_ parameter is much more sensitive than *g*_*x*,*y*_ to the chosen method. The MD(*g*_*x*,*y*_) follows the same trends as MD(*g*_*z*_); therefore, we focus on the average *D*(*g*_*z*_) and *D*(Δ*g*) values to compare the different methods. Figure 3 summarizes the major conclusions graphically.

**Table 2:** Comparison of the Performance of the 19 Studied Functionals, Including the B3PW91 Series with Variable Hartree–Fock Exchange Admixture, for the *g*-Tensor Calculations of the 20 Cu(II) Complexes in Terms of the Evaluation Parameters Defined in Eqs 2–4 and in the Text

functional	MD(*g*_*x*,*y*_)	MD(*g*_*z*_)	‰ MPD(*g*_*x*,*y*_)	‰ MPD(*g*_*z*_)	‰ MAPD(*g*_*z*_)	MAPD(*g*_*z*_^s^)	MPD(Δ*g*)	MAPD(Δ*g*)
PBE	–0.023	–0.115	–11.3	–51.9	51.9	55.7	–57.8	57.8
TPSS	–0.023	–0.118	–11.2	–53.2	53.2	56.8	–59.4	59.4
TPSSh	–0.018	–0.100	–8.6	–44.9	44.9	47.2	–50.1	50.1
B3LYP	–0.011	–0.072	–5.5	–32.3	32.3	33.5	–35.8	35.8
PBE0	–0.007	–0.055	–3.2	–24.5	24.6	24.6	–26.8	27.8
BHandHLYP	0.015	0.032	7.6	14.8	16.7	24.7	20.7	27.4
B3PW91 (20%)	–0.011	–0.071	–5.3	–31.7	31.7	32.8	–35.1	35.1
B3PW91-25%	–0.007	–0.056	–3.4	–25.0	25.0	25.0	–27.3	28.2
B3PW91-30%	–0.003	–0.039	–1.3	–17.5	19.5	20.1	–18.7	22.8
B3PW91-35%	0.002	–0.021	1.0	–9.3	14.8	16.6	–9.1	18.8
**B3PW91-40%**	0.007	–0.002	3.4	–0.5	12.4	15.7	1.5	18.7
B3PW91-45%	0.012	0.019	5.9	9.0	13.7	19.6	13.1	22.3
B3PW91-50%	0.017	0.041	8.4	18.9	19.5	28.2	25.7	29.8
B3PW91-55%	0.022	0.064	10.9	29.2	29.2	40.5	39.0	40.0
LC-BLYP	–0.041	–0.175	–20.0	–78.6	78.6	85.1	–85.5	85.5
CAM-B3LYP	–0.033	–0.144	–16.0	–64.7	64.7	69.3	–69.9	69.9
B2PLYP	–0.018	–0.070	–8.8	–31.7	31.7	39.2	–36.8	36.8
DSD-PBEP86	–0.001	0.005	–0.5	2.4	13.8	18.6	5.3	19.9
PBE-QIDH	0.003	0.019	1.7	8.6	14.6	19.3	13.4	23.4

The magnitude and anisotropy of computed *g*-factors, the spin population on the Cu ion, and the admixture of the exact (Hartree–Fock) exchange in a density functional are all closely interrelated. This connection provides a convenient perspective for the analysis and discussion of results. PBE and TPSS show significant underestimation of both *g*_*z*_ and Δ*g*. The ‰ MPD(*g*_*z*_) and ‰ MAPD(*g*_*z*_) for those functionals have equal absolute values, which shows that the underestimation is systematic. Progressive admixture of the exact exchange is the principal factor that affects the results qualitatively. Thus, increasing percentages of exact exchange in TPSSh (10%), B3LYP and B3PW91 (20%), and PBE0 (25%) progressively limit the underestimation, albeit without eventually correcting it to a satisfactory extent. The BHandHLYP functional, with 50% exact exchange admixture, performs much better, but in this case, systematic overestimation of *g*_*z*_ and Δ*g* is observed.

**Figure 4:**
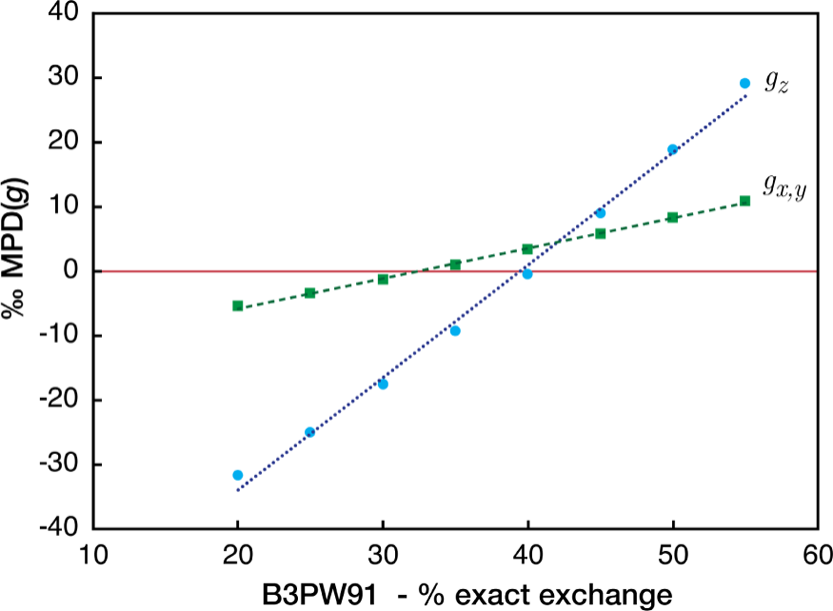
Correlation of the percentage of exact exchange in the B3PW91 functional with the mean per mille difference (‰ MPD) from experiment of the computed *g*-tensor component across the reference set of Cu complexes.

Adjusting the percentage of exact exchange in global hybrid functionals provides a way to improve the predictions. To explore this aspect, we chose the B3PW91 as a platform because this functional in its default definition (20% exact exchange) is the method of choice for computing Cu(II) hyperfine coupling constants. We increased the percentage of exact exchange from 20 to 55% in steps of 5% and recomputed the *g*-tensors for all complexes. The results show a definitive, almost quantitative correlation between the percentage of exact exchange and the errors in *g* values (Figure 4). The average underestimation obtained with the default definition of the functional is essentially eliminated at 40% exact exchange, while further increase in exact exchange results monotonically in overestimation of both the *g*-shifts and the *g*-anisotropy. These trends are mirrored on the correlation of the deviation, Δ*g*_*z*_, of the calculated *g*_*z*_ from the experimental value with the Cu spin populations computed with the respective functional (Figure S2) that follow closely the change in exact exchange admixture, not in a global sense but specifically for any individual compound. Therefore, the adequate description of covalency in Cu(II) complexes, a critical requirement for the reliable prediction of *g*-tensors, is where most common functionals falter because of the systematic underestimation of spin localization on copper or underestimation of the paramagnetic contribution to the target quantity. The increase to 40% exact exchange in the B3PW91 functional provides the best average errors in relevant metrics. This is consistent with the prior literature on the subject, particularly with studies by Kaupp and co-workers. B3PW91 has been associated with good performance for transition metal systems^87^ and increasing the admixture of exact exchange has been reported to be favorable for *g*-tensor calculations.^87−89^ Other benchmarking studies on 3d, 4d, and 5d transition metal complexes also converged to an optimal value of 40% exact exchange, while the choice of the pure DFT components was deemed less important.^90,91^ A comparable approach was used recently for the calculation of EPR parameters in other LPMO enzymes.^22,92^

Neither the range-separated CAM-B3LYP nor the fully long-range corrected LC-BLYP (which goes to 100% exact exchange at long range) show improved performance over standard global hybrid functionals. On the contrary, they perform on average even more poorly than PBE and TPSS. We notice that these functionals also deviate considerably from the correlation between computed *g*_*z*_ values and the Cu spin population observed for the other functionals (see Figure S2). The poor performance may imply that the optimal exact exchange percentages and range-separation parameters deviate significantly from the standard definition of these functionals for the target property and system. For example, a higher percentage of exact exchange might be more appropriate as the minimum (short-range limit) value, or a significantly higher percentage should already be reached at shorter interelectronic distances than the default ones. In either case, the two functionals of this family tested here are not applicable to the problem.

Double-hybrid functionals deserve particular mention. These functionals mix perturbation theory (MP2) contribution on top of a Kohn–Sham (KS) determinant with a high percentage of exact exchange.^50^ Therefore, virtual KS orbitals are also utilized in an attempt to achieve a balanced description of static and dynamic correlation effects. Because dynamic correlation is known to play an important role in the calculation of *g*-tensors,^93^ double-hybrid functionals should provide a good start. According to our results, the original B2PLYP functional does not perform competitively in agreement with the observations of Sciortino et al.^26^ On the other hand, the good performance of PBE-QIDH and, even more so, of DSD-PBEP86 shows that double-hybrid functionals hold promise in terms of achieving systematically improved computational predictions of *g*-tensors. On average, DSD-PBEP86 is second-best compared to the modified B3PW91. Overall, it does not manage to achieve better error control compared to the tuned global hybrid. Therefore, its use in the present context is not justified in view of the considerable additional computational cost. Nevertheless, the encouraging performance of double-hybrid DFT deserves a more thorough and extensive investigation.

Taking into account the present results as well as previous benchmark studies on calculations of Cu hyperfine coupling constants and *g*-tensors, the question remains whether one can conceive of a single method that performs equally well for both EPR properties. Such a method remains elusive, but we can surmise that a DFT-based approach would require a very particular handling of exact exchange admixture. A specifically optimized range-separated functional or double-hybrid might be realizable, but one would have to carefully balance such an approach with compromises in the performance of the functional for other properties. It is likely that local, as opposed to global, hybrid functionals, as described by Kaupp and co-workers,^94^ represent a more promising development in this direction. For the moment, the results presented above lead us to conclude that doubling the default 20% exact exchange of B3PW91 provides the best solution for the calculation of *g*-tensors in the reference set of Cu complexes; therefore, this method is adopted for computing *g*-tensors of the LPMO models, while the default definition of the functional is used for calculations of hyperfine coupling constants.

### 3.2 pH Dependence Study of *Pl*AA10 by EPR Spectroscopy

**Figure 5:**
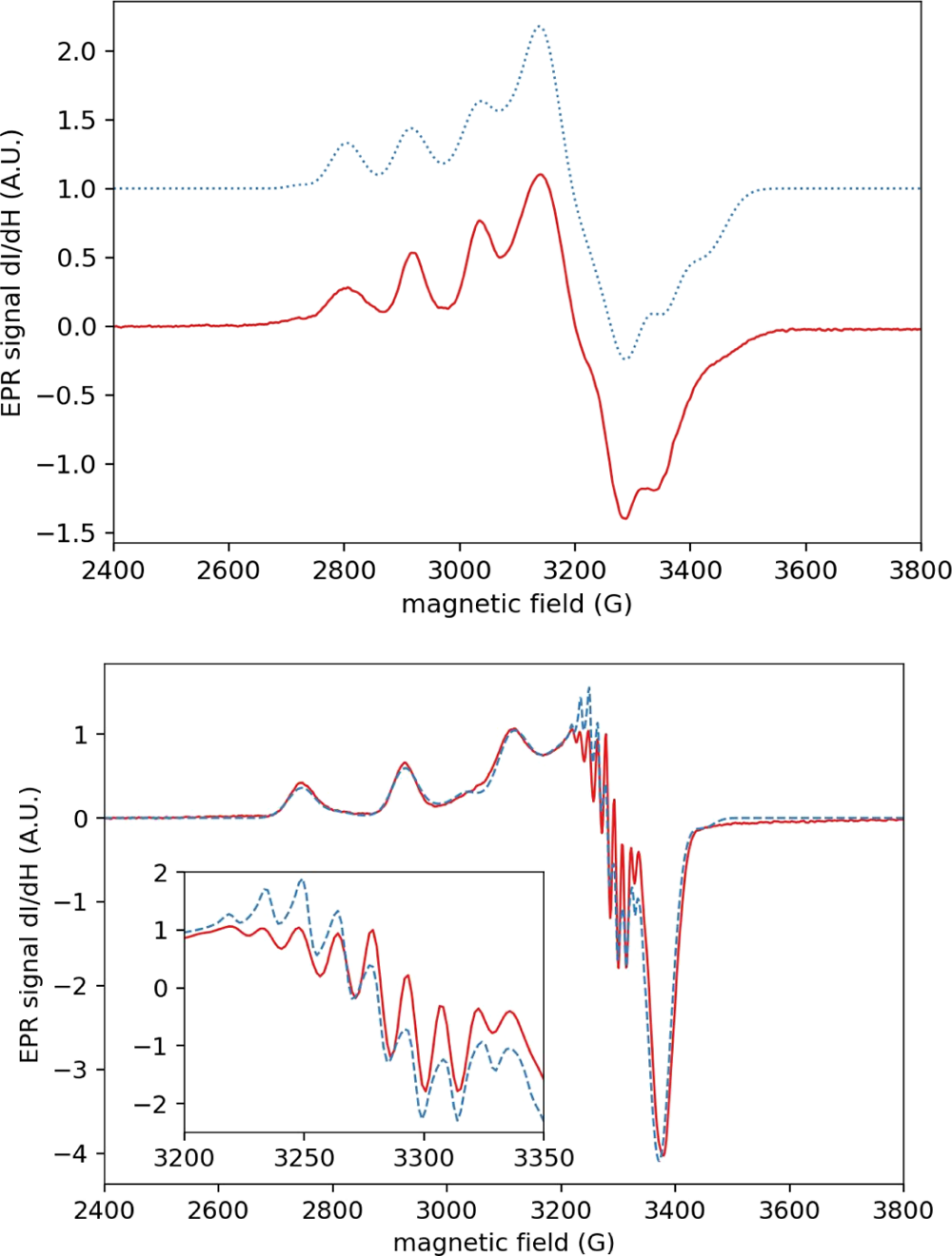
Experimental (red lines) and simulated (blue dashed lines) cw X-band EPR spectra of a 200 μM solution of *Pl*AA10 at *T* = 120 K in MES buffer (pH = 6.5, top) and in TRIS buffer (pH = 8.5, bottom).

Following previous work in the group on *Pl*AA10 which evidenced the contribution of two species to the EPR signature of the enzyme recorded at pH 6.5,^15^ new spectroscopic measurements were undertaken. X-band EPR spectra were recorded at 120 K using frozen samples of *Pl*AA10 under two different pH conditions at either pH 6.5 in MES buffer or at pH 8.5 in Tris-H_2_SO_4_ (2-amino-2-hydroxymethyl-propane-1,3-diol) buffer. EPR parameters simulated by using the EasySpin program package are reported in Table 3, and the corresponding simulated spectra are shown in Figure 5 together with the experimental ones.

**Table 3:** X-Band EPR Parameters of *Pl*AA10 at pH 6.5 and 8.5 Putting in Evidence the Existence of Two Signals in a Major/Minor Distribution

		*g*-tensor			*A*-tensor (MHz)		
pH	species distribution	*g*_*x*_	*g*_*y*_	*g*_*z*_	*A*_*x*_^Cu^	*A*_*y*_^Cu^	*A*_*z*_^Cu^
6.5	major (95%)	2.025	2.103	2.262	220	90	355
	minor (5%)	2.042	2.061	2.230	10	80	560
8.5	major (90%)	2.042	2.061	2.230	10	80	560
	minor (10%)	2.020	2.103	2.262	220	90	355

The EPR signal previously published was reproduced when working at pH 6.5. The major species is present at around 95% with rhombic EPR parameters. The minor species is observed in the remaining 5% of signal with axial EPR parameters. When the pH is increased to 8.5, the observed minor species is exactly reproducing the major species obtained at pH 6.5, that is, the one with a rhombic EPR character. In addition, the major species (around 90%) at pH 8.5 is reproducing very similar EPR values than that of the minor species observed at pH 6.5. The interconversion behavior of *Pl*AA10 is thus solely dependent on the pH. The axial species is major in basic conditions, while the rhombic species predominates in acidic conditions.

The rhombic/axial shifts observed are evidenced in two parameters for both the *g*- and *A*-tensors: the maximum absolute value (*g*_max_ and |*A*_max_^Cu^|) and the anisotropic value (Δ*g*, Δ*A*^Cu^), obtained by calculating the difference between the largest and the smallest values (Δ*g* = *g*_max_ – *g*_min_, Δ*A*^Cu^ = |*A*_max_^Cu^| – |*A*_min_^Cu^|, Table 4). In terms of the *g*-tensor, the *g*_max_ is very slightly decreased from rhombic to axial. However, the effect is more important when taking the Δ*g* (0.237 vs 0.188). In terms of the *A*-tensor, the *A*_max_^Cu^ values are greatly increased in the axial species when compared to the rhombic species (355 vs 560 MHz). A similar effect is observed in Δ*A*^Cu^ (265 vs 550 MHz). For our comparative analysis with respect to the constructed active site models, we will compare our computational results to those of the major species, that is, the rhombic EPR parameters for pH = 6.5 and the axial parameters at pH = 8.5.

**Table 4:** Experimentally Derived EPR Parameters (*g*_max_, Δ*g*, *A*_max_^Cu^, Δ*A*) of *Pl*AA10 as a Function of the pH

		*g*-tensor		*A*-tensor (MHz)	
pH	species distribution	*g*_max_	Δ*g*	*A*_max_^Cu^	Δ*A*^Cu^
6.5	major (95%)	2.262	0.237	355	265
	minor (5%)	2.230	0.188	560	550
8.5	major (90%)	2.230	0.188	560	550
	minor (10%)	2.262	0.237	355	265

**Figure 6:**
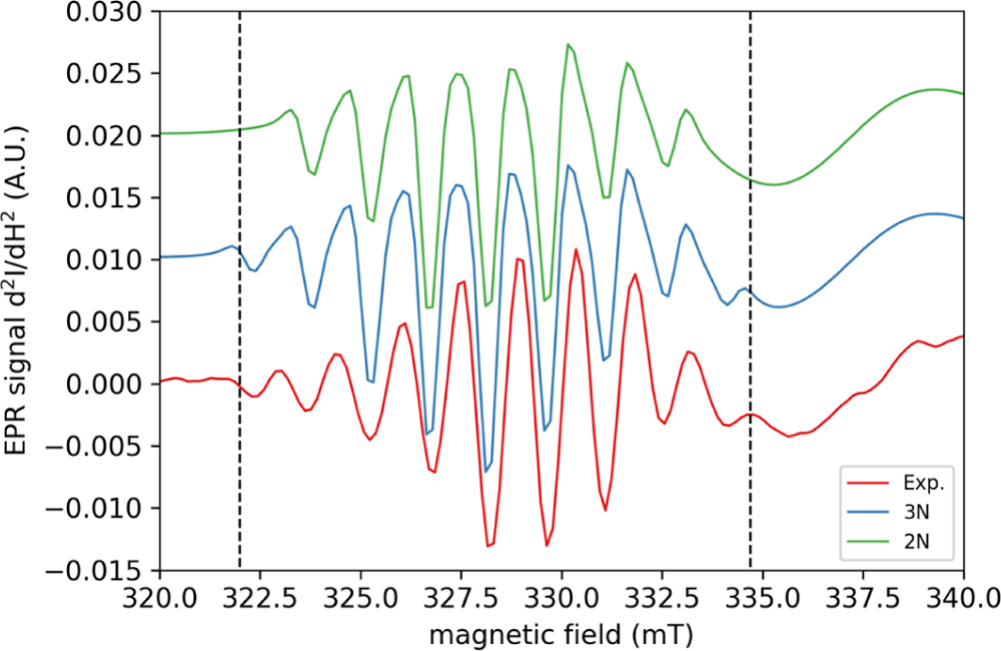
Experimental (red line) and simulated (green and blue lines with two and three nitrogen atoms, respectively) of the second-derivative cw X-band EPR spectrum of a 200 μM solution of *Pl*AA10 at *T* = 120 K in TRIS buffer (pH = 8.5).

Interestingly, we observe a well-defined superhyperfine pattern in the EPR spectrum recorded at pH = 8.5. To determine to origin of this interaction, we included nitrogen centers in our simulations. To increase the spectral resolution of the narrow hyperfine lines of the nitrogen atoms, we performed second-derivative analysis using the pseudo-modulation method. The result of our second-derivative simulation is presented in Figure 6.

From Figure 6, we observe that the simulation obtained when considering three nitrogen atoms with isotropic coupling constants of 45 MHz is in close agreement with the experimental data. This is highlighted by the satellite lines that are found at 322 and 335 mT (see vertical dashed black lines in Figure 6). On the contrary, such features are absent when using only two nitrogen atoms in the simulation. This point will be further discussed in light of the results from the computational investigation.

### 3.3 Evaluation of Models for the *Pl*AA10 Active Site

#### 3.3.1 Structures and Energetics

**Figure 7:**
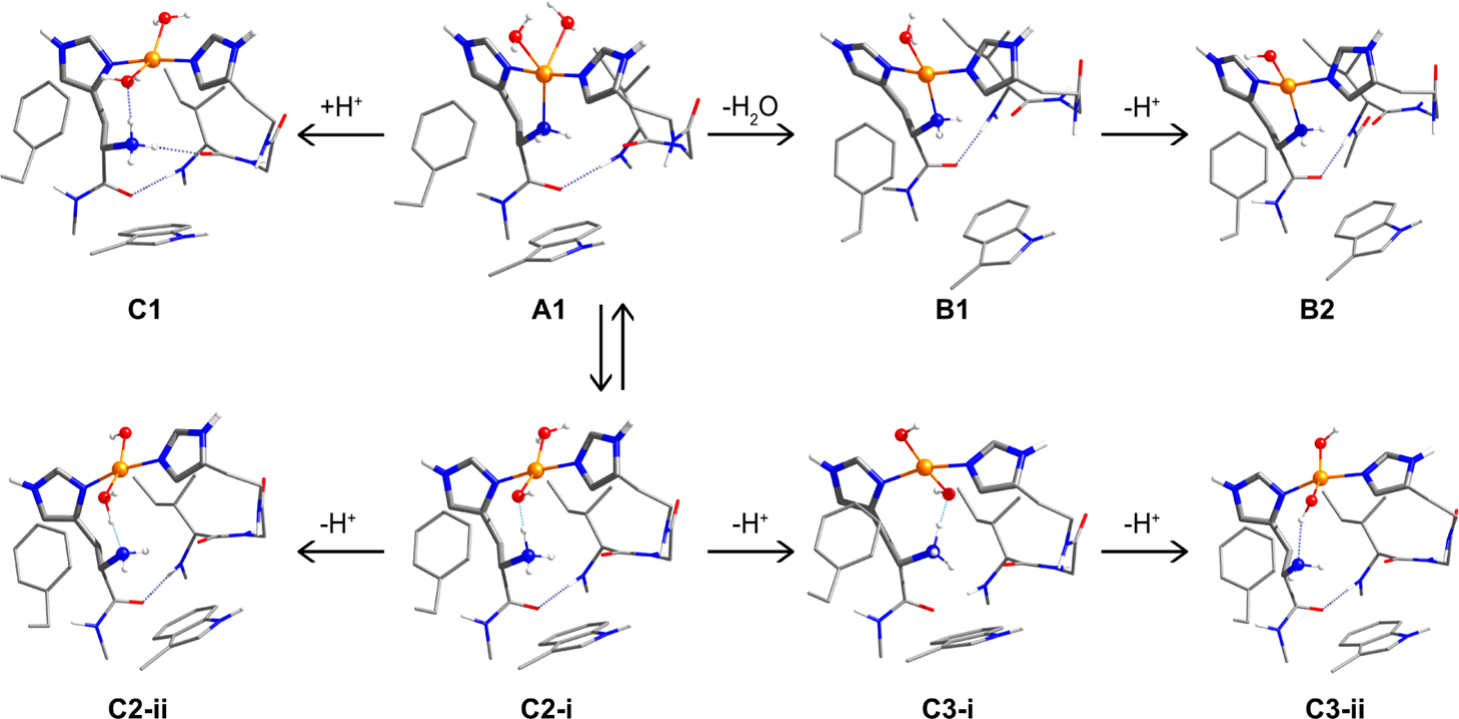
Core structures of the optimized models derived from **A1**.

Starting from the parent model **A1** (Figure 2), we constructed seven types of model of the *PlAA*10 active site by varying the copper coordination sphere and the protonation states of the ligands with ionizable protons, which are the two oxygen ligands, O1 and O2, and the N-terminal amine of His26. Several geometry optimizations were attempted for each type of model starting from slightly different geometries. These investigations resulted in the seven core structures that are presented in Figure 7. Key structural parameters of all models are shown in Table 5. The DFT model **A1**, whose structural parameters are most consistent with the crystallographic structure (Table 5), features a five-coordinated copper in a N_3_O_2_ coordination sphere with distorted square-pyramidal geometry. It has O1 and O2 in the aquo form, while the ligating N-terminal histidine bears two protons, that is, [H_2_O, H_2_O, −NH_2_].

**Table 5:** Selected Structural Parameters (Distances in Å, Angles α and β in Degrees, and Structural Indices τ) of the Optimized Models of the *PlAA*10 LPMO Active Site Considered in the Present Study

model set	structure	Cu–N1	Cu–N2	Cu–N3	Cu–O1	Cu–O2	α	β	τ
A: CuN_3_O_2_	**A1**	1.958	2.143	1.979	2.332	2.363	134.0	174.2	0.67
B: CuN_3_O	**B1**	1.961	2.089	1.977	2.177		158.7	164.6	0.26
C: CuN_2_O_2_	**C1**	1.973	3.601	1.961	2.179	2.285	151.1	171.0	0.27
	**C2-i**	1.996	3.519	1.994	2.100	1.937	165.7	173.9	0.14
	**C2-ii**	2.028	3.581	2.005	1.902	2.035	159.5	167.7	0.23
	**C3-i**	2.098	3.509	2.082	1.934	1.893	175.0	169.0	0.11
	**C3-ii**	2.088	4.000	2.058	1.930	1.909	156.5	160.9	0.30
exp.	**6T5Z**	2.038	2.149	1.997	2.404	2.322	131.7	166.1	0.57

In the presentation of the derivative models, we begin with model set B that includes structures with a N_3_O coordination sphere. Structure **B1** is obtained by decoordination of a single water molecule from model **A1**, which results in [H_2_O, −NH_2_] protonation states. **B2** is obtained from **B1** by deprotonation of the single copper-coordinating ligand, which results in the [OH^–^, −NH_2_] protonation state. It is important to note here that geometry optimizations of structures derived from **A1** with deprotonation of a single water ligand, that is, [H_2_O, OH^–^, −NH_2_], leads to a distorted square pyramidal structure with a water axial ligand that decoordinates from the metal and results in structure **B2**.

Structures that belong to model set C possess a N_2_O_2_ coordination sphere. Parent structure **C1** is obtained by decoordination of the N-terminal histidine and protonation of the amine group resulting in protonation states [H_2_O, H_2_O, decoordinated −NH_3_^+^]. In **C1**, the NH_3_^+^ group forms a hydrogen bond with a ligated water. In models **C2** (labeled **C2-i** and **C2-ii**), the copper bears a water and a hydroxo ligand [H_2_O, OH^–^, decoordinated −NH_*x*_^+^]. Model **C2-i** is obtained from parent structure **C1** by removing a proton from the water ligand that is hydrogen-bonded to the NH_3_^+^ group. **A1** and **C2-i** are structural isomers. Model **C2-ii** results from deprotonation of the decoordinated NH_3_^+^ group and has protonation states [H_2_O, OH^–^, decoordinated −NH_2_]. Finally, in models **C3**, the Cu ion bears two hydroxo ligands [OH^–^, OH^–^, decoordinated −NH_*x*_^(+)^]. **C3-i** [OH^–^, OH^–^, decoordinated −NH_3_^+^] is obtained by the deprotonation of one of the water ligands of **C2-i** and is therefore a structural isomer of **C2-ii**. Finally, model **C3-ii** [OH^–^, OH^–^, decoordinated −NH_2_] is obtained from **C3-i** by deprotonation of the −NH_3_^+^ group.

In terms of computed relative energies, **A1** and **C2-i** are isomers and **C2-i** is calculated to be 6.0 kcal mol^–1^ higher than **A1**. Among the structures that result from **C2-i** deprotonation, **C2-ii** is favored by 9.8 kcal mol^–1^ relative to **C3-i**, which shows that in model **C2-i**, the presence of the hydrogen bond favors the deprotonation of the primary amine. Model **C2-ii** is estimated at 11.3 kcal mol^–1^ higher than **B2** when directly compared. Therefore, the present results show that model **B2** is more stable than **C3-i** by 21.1 kcal mol^–1^. This is a crucial result because in a recent study by Lindley et al.^22^ the axial EPR signal of the *Bl*AA10 LPMO formed at pH 8.5 was assigned to a decoordinated histidine brace structure similar to the present model **C3-i**. This scenario is clearly disfavored in the present case. After evaluation of the models with respect to energetics, we proceed with evaluation of their EPR parameters against the available experimental EPR data.

#### 3.3.2 EPR Parameters and Electronic Structure

The calculated *g*- and *A*-tensor components for all DFT models, using the B3PW91 functional with 40 and 20% exact exchange admixture, respectively, are shown in Table 6. EPR parameters of structures **A1**, **B1**, and **C1**, which have protonated water ligands, maintain a rhombic character. Structures **B2** and **C2**, where copper is coordinated to a single hydroxo ligand, produce axial EPR parameters. Structures **C3**, where both water ligands are in their hydroxyl form, also exhibit rhombic character. To better assess the agreement with the experiment, we examined the rhombic/axial shift parameters (*g*_max_, *A*_max_^Cu^, Δ*g*, and Δ*A*^Cu^) of all considered structures, as shown in Table 7. The rhombic character of the **A1**, **B1**, **C1**, and **C3** EPR parameters is evident from the *A*_max_^Cu^ and Δ*A*^Cu^ values which range between 309 and 446 MHz and between 269 and 375 MHz, respectively. On the contrary, structures **B2** and **C2** have *A*_max_^Cu^ and Δ*A*^Cu^ values which range between 569 and 604 MHz and between 546 and 599 MHz, respectively. At the same time, **B2** and **C2** have slightly smaller *g*_max_ and Δ*g* values than the rest of the models.

**Table 6:** Calculated *g*-Factors and Cu Hyperfine Coupling Constants (MHz) for all Models

model set	model	*g*_*x*_	*g*_*y*_	*g*_*z*_	*A*_*x*_^Cu^	*A*_*y*_^Cu^	*A*_*z*_^Cu^
A: CuN_3_O_2_	**A1**	2.032	2.138	2.257	101	–299	370
B: CuN_3_O	**B1**	2.053	2.092	2.254	71	165	–446
	**B2**	2.059	2.077	2.243	23	–37	–569
C: CuN_2_O_2_	**C1**	2.041	2.160	2.329	15	181	309
	**C2-i**	2.064	2.066	2.243	–15	–16	–597
	**C2-ii**	2.057	2.078	2.246	5	–53	–604
	**C3-i**	2.047	2.088	2.248	85	200	–436
	**C3-ii**	2.045	2.100	2.258	91	227	–393
*Pl*AA10							
pH 6.5	rhombic	2.025	2.103	2.262	220	90	355
pH 8.5	axial	2.042	2.061	2.230	10	80	560

**Table 7:** Axial/Rhombic Shifts [*g*_max_ (=*g*_*z*_), *A*_max_^Cu^ (=*A*_*z*_^Cu^) and Δ*g*, Δ*A*^Cu^] for all Models

	model	*g*_max_	Δ*g*	|*A*_max_^Cu^|	Δ*A*^Cu^
A: CuN_3_O_2_	**A1**	2.257	0.225	370	269
B: CuN_3_O	**B1**	2.254	0.201	446	375
	**B2**	2.243	0.184	569	546
C: CuN_2_O_2_	**C1**	2.329	0.288	309	293
	**C2-i**	2.243	0.179	597	582
	**C2-ii**	2.246	0.189	604	599
	**C3-i**	2.248	0.201	436	351
	**C3-ii**	2.258	0.213	393	303
*Pl*AA10					
pH 6.5	rhombic	2.262	0.237	355	265
pH 8.5	axial	2.230	0.188	560	550

The results confirm that structure **A1** best reproduces the major species in the EPR spectrum of *Pl*AA10 at pH = 6.5. Among the models **B2** and **C2** that produce axial EPR parameters, structure **B2** has *A*_max_^Cu^ and Δ*A* values closer to the experiment than both protonated (**C2-i**) and deprotonated (**C2-ii**) **C2** forms. Overall, the computed EPR parameters of structures **A1** and **B2** are in best agreement with experimental values obtained for *Pl*AA10 at pH = 6.5 and 8.5, respectively.

**Figure 8:**
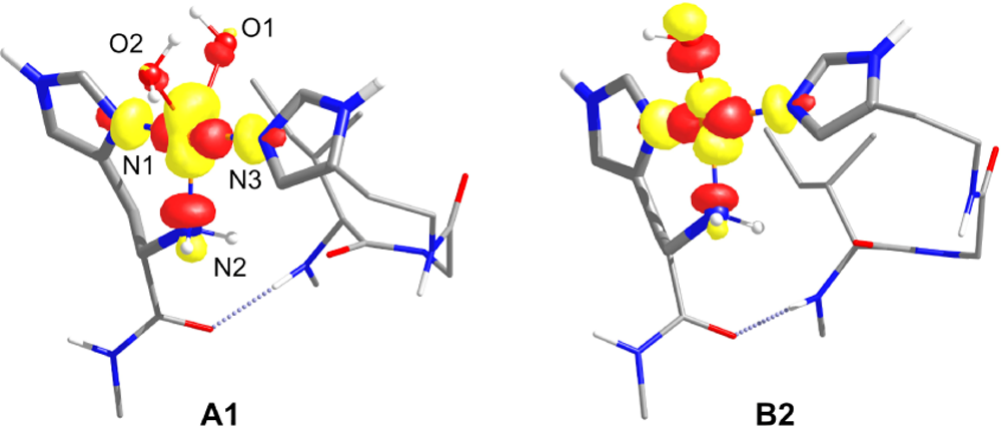
SOMOs of models **A1** and **B2** (for clarity, only the immediate environment of the Cu center is depicted).

The observed rhombic and axial spin-Hamiltonian parameters can be qualitatively rationalized based on the geometry of the copper coordination sphere. The singly occupied molecular orbital (SOMO) of square planar and square-pyramidal copper complexes has mostly d_*x*^2^–*y*^2^_ character. Distortion from the perfect square planar and square-pyramidal geometry results in increasing extent of mixing of the d_*z*^2^_ orbital into the d_*x*^2^–*y*^2^_ SOMO. The rhombic spin-Hamiltonian parameters arise from this d orbital mixing. The extent of geometrical distortion can be expressed using the geometry index τ (defined in Scheme S2),^95^ given in Table 5 for each structure. Structure **A1** has a geometry index value of 0.67 and therefore shows a more pronounced deviation from the square-pyramidal geometry compared to **B2** which has a τ value of 0.34. The d_*z*^2^_ orbital mixing into the d_*x*^2^–*y*^2^_ SOMO of **A1** is clearly seen on the molecular orbital diagram of the SOMO shown in Figure 8. On the contrary, the SOMO of **B2** has a dominant d_*x*^2^–*y*^2^_ character, which explains the computed—and experimentally observed—axial EPR parameters.

A final point concerns the ^14^N isotropic hyperfine constants. The analysis of spectroscopic data presented above established the presence of three nitrogen centers in the immediate surrounding of the unpaired electron. However, in line with structure-based expectations, DFT calculations on models **C2-i** and **C3-i** confirm that in these coordination geometries, only the coordinating imidazole nitrogen atoms (N1 and N3) have large isotropic hyperfine coupling values (ca. 35 MHz for **C2-i** and 45 MHz for **C3-i**). In both cases, the computed hyperfine coupling constant for the decoordinated N2 nitrogen nucleus is negligible (ca. 0.5 MHz). Therefore, these models cannot be reconciled with the spectroscopic observations, in contrast to model **B2** that indeed has three large ^14^N hyperfine couplings as required by the experiment (Table S23). These results further support that the major species observed in high pH conditions is consistent with a Cu(II) ion bound to three nitrogen centers and a hydroxo ligand featuring a N_3_O coordination sphere.

### 3.4 Discussion

The theoretical methodology we developed recently for Cu(II) hyperfine coupling constants^25^ and, in this work, for accurate *g*-tensors, can be reliably applied in the present case to provide a solid identification of the coordination sphere of copper species when experimental EPR data are available. This work is thus filling a methodological gap that was recently pointed out and prevented a clear identification of unknown copper coordination spheres in proteins.^22,24,92^ Armed with these methodologies, we focused on resolving the ambiguous, pH-dependent EPR signals from the LPMO enzyme *Pl*AA10. In the construction of the presented set of active site models, many plausible chemical modifications that could occur when changing the experimental pH conditions were considered.

Our results point to a N_3_O_2_ coordination sphere for the species observed at low pH, that is, model **A1** with two H_2_O ligands on Cu. This model best reproduces the experimental rhombic EPR signal that can be attributed to the *Pl*AA10 active site from the 6T5Z X-ray crystal structure used as a starting point for our computational investigation. The rhombic-to-axial shift of the observed EPR signal upon increasing pH indicates that the axial signal is induced either by deprotonation of the active site or by a pH-induced structural change of the protein. Our results show that the axial signal can be assigned uniquely to a structure with N_3_O coordination where the copper ion is coordinated to a single hydroxyl ligand, that is, model **B2** of the present work. Neither the mere decoordination of a single water ligand (**B1**) nor the decoordination of the protonated amine(**C1**) creates the shift to axial EPR parameters. These models exhibit well defined rhombic signatures. Alternative structures of N_2_O_2_ coordination and two hydroxyl ligands on copper, **C3-i** and **C3-ii**, also produce rhombic EPR parameters. Only models **B2**, **C2-i**, and **C2-ii** produce axial EPR signals. Of these candidate structures, models **C2-i** and **C2-ii** are both energetically unfavorable and inconsistent with the experimental requirement for three copper-ligated nitrogen nuclei. Therefore, model B2 provides the only convincing and fully experimentally consistent interpretation of the axial EPR signal produced at the higher pH value.

The proposed hypothesis of an **A1** and **B2** equilibrium is consistent with a recent EPR study of the *Pp*AA10 enzyme, where the rhombic signal was assigned to the distorted square pyramidal structure described by crystallography and the axial signal at higher pH was assigned to the formation of a nearly planar species that resulted from the replacement of a copper-coordinating water molecule with a hydroxide or Cl^–^ ion along with decoordination of a water ligand.^23^ On the other hand, the **A1** and **B2** equilibrium is in apparent contradiction with a recent work on the *Bl*AA10 enzyme which investigated the protonation states of the active site as function of pH by means of EPR spectroscopy and DFT calculations.^22^ The preferred description of the low pH species in that work was of a N_3_O_2_ model comprising one water and one hydroxy ligand on Cu, even though comparison of the computed EPR parameters with the experimental data in acidic conditions shows non-negligible deviation. For the major species observed at moderate-high pH, the structure proposed contained a four-coordinated copper center in an N_2_O_2_ coordination sphere with two hydroxy ligands. The model featured decoordination of the amine from the N-terminal histidine along with its protonation. However, the reported simulation of the observed superhyperfine pattern for the species associated with the axial signal at moderate-high pH was carried out including only two nitrogen nuclei and does not achieve satisfactory agreement with the experiment.^22^ This type of interpretation receives no support in the present case of *Pl*AA10. While we acknowledge that the enzymes are distinct and that different phenomena might be at play, the excellent agreement between experimental and computed EPR parameters for the models presented in this work allow us to propose an alternative structure for the axial species observed in basic conditions. Our suggested N_3_(OH) structure, model **B2**, displays a fully coordinated histidine brace and a single hydroxy ligand, an assignment strongly supported by our refined simulations of the experimental superhyperfine pattern: using the pseudo-modulation method, we show that the agreement between the experimental and the simulated second-derivative EPR spectrum can only be reached upon inclusion of three nitrogen centers. This conclusion is further supported by DFT computations on both energetic and spectroscopic grounds. Therefore, the combination of EPR measurements with the properly calibrated DFT methodology employed in this work provide a definitive picture of the experimentally observed pH-dependent species and allows us to identify unambiguously the coordination spheres of the copper active sites of the *Pl*AA10 family, with implications for re-evaluating relevant assignments in other AA10 LPMOs.

## 4 Conclusions

In the present work, we described an accurate quantum chemical approach for the calculation of Cu(II) *g*-tensors and its application together with our recently developed methodology for copper hyperfine coupling constants to the structural assignment of EPR signals in the LPMO from *Photorhabdus luminescens*, *Pl*AA10. Based on the benchmarked accuracy of our applied methodology in the prediction of the relevant EPR parameters, we can safely assign the species observed at pH = 6.5 and 8.5. The low pH structure is a distorted square-pyramidal copper center with two water molecules displaying rhombic EPR parameters, whereas the higher pH species is a distorted square-planar copper bound to a single hydroxy ligand leading to axial EPR parameters. This assignment is fully consistent with all aspects of the quantum chemical calculations and with the spectroscopic observations, confidently excluding structural alternatives on the basis of copper EPR parameters and relative energetics of isomeric forms, as well as on the assignment of the number and magnitude of nitrogen superhyperfine parameters. The successful application of our approach provides a solid framework to establish correlations between structural features and spectroscopic data on LPMOs. By combining EPR spectroscopy and quantum chemistry, this work contributes to an accurate description of the biologically relevant species of AA10 LPMOs and allows us to reach a better understanding of the enzyme, while the computational strategy is broadly applicable for the reliable prediction of EPR parameters of intermediates involved in the catalytic mechanism of LPMO and in copper proteins in general.

## Supporting Information Available

Set of the Cu(II) complexes considered in this work; comparison of key structural parameters between functionals for model **A1**; definition of geometry index τ; evaluation of basis sets; evaluation of relativistic effects; detailed results for the benchmark set of complexes; correlation of Δ*g*_*z*_ with Cu spin density for representative complexes; ^14^N hyperfine coupling constants; and Cartesian coordinates of all models (PDF)
Cartesian coordinates of all optimized structures (TXT)

## References

[ref1] Quinlan R. J.; Sweeney M. D.; Lo Leggio L.; Otten H.; Poulsen J.-C. N.; Johansen K. S.; Krogh K. B. R. M.; Jørgensen C. I.; Tovborg M.; Anthonsen A.; Tryfona T.; Walter C. P.; Dupree P.; Xu F.; Davies G. J.; Walton P. H. Insights into the oxidative degradation of cellulose by a copper metalloenzyme that exploits biomass components. Proc. Natl. Acad. Sci. U.S.A. 2011, 108, 15079–15084. 10.1073/pnas.1105776108. 21876164 PMC3174640

[ref2] Vu V. V.; Beeson W. T.; Phillips C. M.; Cate J. H. D.; Marletta M. A. Determinants of Regioselective Hydroxylation in the Fungal Polysaccharide Monooxygenases. J. Am. Chem. Soc. 2014, 136, 562–565. 10.1021/ja409384b. 24350607

[ref3] Beeson W. T.; Phillips C. M.; Cate J. H. D.; Marletta M. A. Oxidative Cleavage of Cellulose by Fungal Copper-Dependent Polysaccharide Monooxygenases. J. Am. Chem. Soc. 2012, 134, 890–892. 10.1021/ja210657t. 22188218

[ref4] Vaaje-Kolstad G.; Horn S. J.; van Aalten D. M. F.; Synstad B.; Eijsink V. G. H. The Non-catalytic Chitin-binding Protein CBP21 from Serratia marcescens Is Essential for Chitin Degradation. J. Biol. Chem. 2005, 280, 28492–28497. 10.1074/jbc.m504468200. 15929981

[ref5] Hemsworth G. R.; Taylor E. J.; Kim R. Q.; Gregory R. C.; Lewis S. J.; Turkenburg J. P.; Parkin A.; Davies G. J.; Walton P. H. The Copper Active Site of CBM33 Polysaccharide Oxygenases. J. Am. Chem. Soc. 2013, 135, 6069–6077. 10.1021/ja402106e. 23540833 PMC3636778

[ref6] Hemsworth G. R.; Johnston E. M.; Davies G. J.; Walton P. H. Lytic Polysaccharide Monooxygenases in Biomass Conversion. Trends Biotechnol. 2015, 33, 747–761. 10.1016/j.tibtech.2015.09.006. 26472212

[ref7] Merino S. T.; Cherry J. In Biofuels; Olsson L., Ed.; Springer Berlin Heidelberg: Berlin, Heidelberg, 2007; pp 95–120.

[ref8] Barbosa F. C.; Silvello M. A.; Goldbeck R. Cellulase and oxidative enzymes: new approaches, challenges and perspectives on cellulose degradation for bioethanol production. Biotechnol. Lett. 2020, 42, 875–884. 10.1007/s10529-020-02875-4. 32239348

[ref9] Chan S. I.; Yu S. S.-F. Controlled Oxidation of Hydrocarbons by the Membrane-Bound Methane Monooxygenase: The Case for a Tricopper Cluster. Acc. Chem. Res. 2008, 41, 969–979. 10.1021/ar700277n. 18605740

[ref10] Zhang L.; Koay M.; Maher M. J.; Xiao Z.; Wedd A. G. Intermolecular Transfer of Copper Ions from the CopC Protein of Pseudomonas syringae. Crystal Structures of Fully Loaded Cu^I^Cu^II^ Forms. J. Am. Chem. Soc. 2006, 128, 5834–5850. 10.1021/ja058528x. 16637653

[ref11] Lawton T. J.; Kenney G. E.; Hurley J. D.; Rosenzweig A. C. The CopC Family: Structural and Bioinformatic Insights into a Diverse Group of Periplasmic Copper Binding Proteins. Biochemistry 2016, 55, 2278–2290. 10.1021/acs.biochem.6b00175. 27010565 PMC5260838

[ref12] Fisher O. S.; Sendzik M. R.; Ross M. O.; Lawton T. J.; Hoffman B. M.; Rosenzweig A. C. PCu_A_C domains from methane-oxidizing bacteria use a histidine brace to bind copper. J. Biol. Chem. 2019, 294, 16351–16363. 10.1074/jbc.ra119.010093. 31527086 PMC6827282

[ref13] Labourel A.; Frandsen K. E. H.; Zhang F.; Brouilly N.; Grisel S.; Haon M.; Ciano L.; Ropartz D.; Fanuel M.; Martin F.; Navarro D.; Rosso M.-N.; Tandrup T.; Bissaro B.; Johansen K. S.; Zerva A.; Walton P. H.; Henrissat B.; Leggio L. L.; Berrin J.-G. A fungal family of lytic polysaccharide monooxygenase-like copper proteins. Nat. Chem. Biol. 2020, 16, 345–350. 10.1038/s41589-019-0438-8. 31932718

[ref14] Garcia-Santamarina S.; Probst C.; Festa R. A.; Ding C.; Smith A. D.; Conklin S. E.; Brander S.; Kinch L. N.; Grishin N. V.; Franz K. J.; Riggs-Gelasco P.; Lo Leggio L.; Johansen K. S.; Thiele D. J. A lytic polysaccharide monooxygenase-like protein functions in fungal copper import and meningitis. Nat. Chem. Biol. 2020, 16, 337–344. 10.1038/s41589-019-0437-9. 31932719 PMC7036007

[ref15] Munzone A.; El Kerdi B.; Fanuel M.; Rogniaux H.; Ropartz D.; Réglier M.; Royant A.; Simaan A. J.; Decroos C. Characterization of a bacterial copper-dependent lytic polysaccharide monooxygenase with an unusual second coordination sphere. FEBS J. 2020, 287, 3298–3314. 10.1111/febs.15203. 31903721

[ref16] More C.; Belle V.; Asso M.; Fournel A.; Roger G.; Guigliarelli B.; Bertrand P. EPR spectroscopy: A powerful technique for the structural and functional investigation of metalloproteins. Biospectroscopy 1999, 5, S3–S18. 10.1002/(sici)1520-6343(1999)5:5+<s3::aid-bspy2>3.0.co;2-p. 10512534

[ref17] Van Doorslaer S.; Vinck E. The strength of EPR and ENDOR techniques in revealing structure–function relationships in metalloproteins. Phys. Chem. Chem. Phys. 2007, 9, 4620–4638. 10.1039/b701568b. 17700864

[ref18] Hemsworth G. R.; Ciano L.; Davies G. J.; Walton P. H. In Methods Enzymology; Armstrong F., Ed.; Academic Press, 2018; Vol. 613, pp 63–90. 10.1016/bs.mie.2018.10.014 30509474

[ref19] Forsberg Z.; Røhr Å. K.; Mekasha S.; Andersson K. K.; Eijsink V. G. H.; Vaaje-Kolstad G.; Sørlie M. Comparative Study of Two Chitin-Active and Two Cellulose-Active AA10-Type Lytic Polysaccharide Monooxygenases. Biochemistry 2014, 53, 1647–1656. 10.1021/bi5000433. 24559135

[ref20] Gregory R. C.; Hemsworth G. R.; Turkenburg J. P.; Hart S. J.; Walton P. H.; Davies G. J. Activity, stability and 3-D structure of the Cu(II) form of a chitin-active lytic polysaccharide monooxygenase from *Bacillus amyloliquefaciens*. Dalton Trans. 2016, 45, 16904–16912. 10.1039/c6dt02793h. 27722375

[ref21] Chaplin A. K.; Wilson M. T.; Hough M. A.; Svistunenko D. A.; Hemsworth G. R.; Walton P. H.; Vijgenboom E.; Worrall J. A. R. Heterogeneity in the Histidine-brace Copper Coordination Sphere in Auxiliary Activity Family 10 (AA10) Lytic Polysaccharide Monooxygenases. J. Biol. Chem. 2016, 291, 12838–12850. 10.1074/jbc.m116.722447. 27129229 PMC4933455

[ref22] Lindley P. J.; Parkin A.; Davies G. J.; Walton P. H. Mapping the protonation states of the histidine brace in an AA10 lytic polysaccharide monooxygenase using CW-EPR spectroscopy and DFT calculations. Faraday Discuss. 2021, 10.1039/d1fd00068c. 35171174

[ref23] Serra I.; Piccinini D.; Paradisi A.; Ciano L.; Bellei M.; Bortolotti C. A.; Battistuzzi G.; Sola M.; Walton P. H.; Di Rocco G. Activity and substrate specificity of lytic polysaccharide monooxygenases: An ATR FTIR-based sensitive assay tested on a novel species from *Pseudomonas putida*. Protein Sci. 2021, 31, 591–601. 10.1002/pro.4255. 34897841 PMC8862430

[ref24] Theibich Y. A.; Sauer S. P. A.; Leggio L. L.; Hedegård E. D. Estimating the accuracy of calculated electron paramagnetic resonance hyperfine couplings for a lytic polysaccharide monooxygenase. Comput. Struct. Biotechnol. J. 2021, 19, 555–567. 10.1016/j.csbj.2020.12.014. 33510861 PMC7807142

[ref25] Gómez-Piñeiro R. J.; Pantazis D. A.; Orio M. Comparison of Density Functional and Correlated Wave Function Methods for the Prediction of Cu(II) Hyperfine Coupling Constants. ChemPhysChem 2020, 21, 2667–2679. 10.1002/cphc.202000649. 33201578 PMC7756273

[ref26] Sciortino G.; Lubinu G.; Maréchal J.-D.; Garribba E. DFT Protocol for EPR Prediction of Paramagnetic Cu(II) Complexes and Application to Protein Binding Sites. Magnetochemistry 2018, 4, 55 10.3390/magnetochemistry4040055.

[ref27] Stoll S.; Schweiger A. EasySpin, a comprehensive software package for spectral simulation and analysis in EPR. J. Magn. Reson. 2006, 178, 42–55. 10.1016/j.jmr.2005.08.013. 16188474

[ref28] Hyde J. S.; Pasenkiewicz-Gierula M.; Jesmanowicz A.; Antholine W. E. Pseudo field modulation in EPR spectroscopy. Appl. Magn. Reson. 1990, 1, 483–496. 10.1007/bf03166028.

[ref29] El Bakkali-Tahéri N.; Tachon S.; Orio M.; Bertaina S.; Martinho M.; Robert V.; Réglier M.; Tron T.; Dorlet P.; Simaan A. J. Characterization of Cu(II)-reconstituted ACC Oxidase using experimental and theoretical approaches. Arch. Biochem. Biophys. 2017, 623–624, 31–41. 10.1016/j.abb.2017.03.012. 28522117

[ref30] Neese F. Software Update: the ORCA Program System, Version 4.0. Wiley Interdiscip. Rev.: Comput. Mol. Sci. 2018, 8, e1327 10.1002/wcms.1327.

[ref31] Neese F.; Wennmohs F.; Becker U.; Riplinger C. The ORCA quantum chemistry program package. J. Chem. Phys. 2020, 152, 224108 10.1063/5.0004608. 32534543

[ref32] Becke A. D. Density-Functional Exchange-Energy Approximation with Correct Asymptotic-Behavior. Phys. Rev. A: At., Mol., Opt. Phys. 1988, 38, 3098–3100. 10.1103/physreva.38.3098. 9900728

[ref33] Perdew J. P. Density-Functional Approximation for the Correlation-Energy of the Inhomogeneous Electron-Gas. Phys. Rev. B: Condens. Matter Mater. Phys. 1986, 33, 8822–8824. 10.1103/physrevb.33.8822. 9938299

[ref34] Weigend F.; Ahlrichs R. Balanced basis sets of split valence, triple zeta valence and quadruple zeta valence quality for H to Rn: Design and assessment of accuracy. Phys. Chem. Chem. Phys. 2005, 7, 3297–3305. 10.1039/b508541a. 16240044

[ref35] Weigend F. Accurate Coulomb-fitting basis sets for H to Rn. Phys. Chem. Chem. Phys. 2006, 8, 1057–1065. 10.1039/b515623h. 16633586

[ref36] Becke A. D. Density-functional thermochemistry. III. The role of exact exchange. J. Chem. Phys. 1993, 98, 5648–5652. 10.1063/1.464913.

[ref37] Lee C.; Yang W.; Parr R. G. Development of the Colle-Salvetti Correlation-Energy Formula Into a Functional of the Electron-Density. Phys. Rev. B: Condens. Matter Mater. Phys. 1988, 37, 785–789. 10.1103/physrevb.37.785. 9944570

[ref38] Li L.; Li C.; Zhang Z.; Alexov E. On the Dielectric “Constant” of Proteins: Smooth Dielectric Function for Macromolecular Modeling and Its Implementation in DelPhi. J. Chem. Theory Comput. 2013, 9, 2126–2136. 10.1021/ct400065j. 23585741 PMC3622359

[ref39] Heß B. A.; Marian C. M.; Wahlgren U.; Gropen O. A mean-field spin-orbit method applicable to correlated wavefunctions. Chem. Phys. Lett. 1996, 251, 365–371. 10.1016/0009-2614(96)00119-4.

[ref40] Neese F. Efficient and Accurate Approximations to the Molecular Spin-Orbit Coupling Operator and Their Use in Molecular g-Tensor Calculations. J. Chem. Phys. 2005, 122, 034107 10.1063/1.1829047. 15740192

[ref41] Hedegård E. D.; Kongsted J.; Sauer S. P. A. Optimized Basis Sets for Calculation of Electron Paramagnetic Resonance Hyperfine Coupling Constants: aug-cc-pVTZ-J for the 3d Atoms Sc–Zn. J. Chem. Theory Comput. 2011, 7, 4077–4087. 10.1021/ct200587k. 26598353

[ref42] Hedegård E. D.; Kongsted J.; Sauer S. P. A. Improving the calculation of electron paramagnetic resonance hyperfine coupling tensors for d-block metals. Phys. Chem. Chem. Phys. 2012, 14, 10669–10676. 10.1039/c2cp40969k. 22785432

[ref43] Perdew J. P.; Wang Y. Accurate and simple analytic representation of the electron-gas correlation energy. Phys. Rev. B: Condens. Matter Mater. Phys. 1992, 45, 13244–13249. 10.1103/physrevb.45.13244. 10001404

[ref44] Perdew J. P.; Burke K.; Ernzerhof M. Generalized gradient approximation made simple. Phys. Rev. Lett. 1996, 77, 3865–3868. 10.1103/physrevlett.77.3865. 10062328

[ref45] Tao J.; Perdew J. P.; Staroverov V. N.; Scuseria G. E. Climbing the Density Functional Ladder: Nonempirical Meta-Generalized Gradient Approximation Designed for Molecules and Solids. Phys. Rev. Lett. 2003, 91, 146401 10.1103/physrevlett.91.146401. 14611541

[ref46] Adamo C.; Barone V. Toward reliable density functional methods without adjustable parameters: The PBE0 model. J. Chem. Phys. 1999, 110, 6158–6170. 10.1063/1.478522.

[ref47] Staroverov V. N.; Scuseria G. E.; Tao J.; Perdew J. P. Comparative Assessment of a New Nonempirical Density Functional: Molecules and Hydrogen-Bonded Complexes. J. Chem. Phys. 2003, 119, 12129–12137. 10.1063/1.1626543.

[ref48] Tawada Y.; Tsuneda T.; Yanagisawa S.; Yanai T.; Hirao K. A long-range-corrected time-dependent density functional theory. J. Chem. Phys. 2004, 120, 8425–8433. 10.1063/1.1688752. 15267767

[ref49] Yanai T.; Tew D. P.; Handy N. C. A new hybrid exchange–correlation functional using the Coulomb-attenuating method (CAM-B3LYP). Chem. Phys. Lett. 2004, 393, 51–57. 10.1016/j.cplett.2004.06.011.

[ref50] Grimme S. Semiempirical hybrid density functional with perturbative second-order correlation. J. Chem. Phys. 2006, 124, 034108 10.1063/1.2148954. 16438568

[ref51] Kozuch S.; Martin J. M. L. DSD-PBEP86: in search of the best double-hybrid DFT with spin-component scaled MP2 and dispersion corrections. Phys. Chem. Chem. Phys. 2011, 13, 20104–20107. 10.1039/c1cp22592h. 21993810

[ref52] Brémond É.; Sancho-García J. C.; Pérez-Jiménez Á. J.; Adamo C. Communication: Double-hybrid functionals from adiabatic-connection: The QIDH model. J. Chem. Phys. 2014, 141, 031101 10.1063/1.4890314. 25053294

[ref53] Stoychev G. L.; Auer A. A.; Neese F. Automatic Generation of Auxiliary Basis Sets. J. Chem. Theory Comput. 2017, 13, 554–562. 10.1021/acs.jctc.6b01041. 28005364

[ref54] Douglas M.; Kroll N. M. Quantum electrodynamical corrections to the fine structure of helium. Ann. Phys. 1974, 82, 89–155. 10.1016/0003-4916(74)90333-9.

[ref55] Hess B. A. Applicability of the No-Pair Equation with Free-Particle Projection Operators to Atomic and Molecular-Structure Calculations. Phys. Rev. A: At., Mol., Opt. Phys. 1985, 32, 756–763. 10.1103/physreva.32.756. 9896123

[ref56] Hess B. A. Relativistic electronic-structure calculations employing a two-component no-pair formalism with external-field projection operators. Phys. Rev. A: At., Mol., Opt. Phys. 1986, 33, 3742–3748. 10.1103/physreva.33.3742. 9897114

[ref57] Jansen G.; Hess B. A. Revision of the Douglas-Kroll transformation. Phys. Rev. A: At., Mol., Opt. Phys. 1989, 39, 6016–6017. 10.1103/physreva.39.6016. 9901188

[ref58] Wolf A.; Reiher M.; Hess B. A. The generalized Douglas-Kroll transformation. J. Chem. Phys. 2002, 117, 9215–9226. 10.1063/1.1515314. 15267790

[ref59] Reiher M. Douglas–Kroll–Hess Theory: a relativistic electrons-only theory for chemistry. Theor. Chem. Acc. 2006, 116, 241–252. 10.1007/s00214-005-0003-2.

[ref60] Nakajima T.; Hirao K. The Douglas–Kroll–Hess Approach. Chem. Rev. 2012, 112, 385–402. 10.1021/cr200040s. 21678899

[ref61] van Lenthe E.; Baerends E. J.; Snijders J. G. Relativistic Regular Two-component Hamiltonians. J. Chem. Phys. 1993, 99, 4597–4610. 10.1063/1.466059.

[ref62] van Lenthe E.; Baerends E. J.; Snijders J. G. Relativistic Total-Energy Using Regular Approximations. J. Chem. Phys. 1994, 101, 9783–9792. 10.1063/1.467943.

[ref63] van Lenthe E.; Snijders J. G.; Baerends E. J. The zero-order regular approximation for relativistic effects: The effect of spin-orbit coupling in closed shell molecules. J. Chem. Phys. 1996, 105, 6505–6516. 10.1063/1.472460.

[ref64] Pantazis D. A.; Chen X.-Y.; Landis C. R.; Neese F. All-electron scalar relativistic basis sets for third-row transition metal atoms. J. Chem. Theory Comput. 2008, 4, 908–919. 10.1021/ct800047t. 26621232

[ref65] Remenyi C.; Reviakine R.; Kaupp M. Density Functional Study of EPR Parameters and Spin-Density Distribution of Azurin and Other Blue Copper Proteins. J. Phys. Chem. B 2007, 111, 8290–8304. 10.1021/jp071745v. 17592871

[ref66] Sinnecker S.; Neese F. QM/MM calculations with DFT for taking into account protein effects on the EPR and optical spectra of metalloproteins. Plastocyanin as a case study. J. Comput. Chem. 2006, 27, 1463–1475. 10.1002/jcc.20426. 16807973

[ref67] Schulz C. E.; van Gastel M.; Pantazis D. A.; Neese F. Converged Structural and Spectroscopic Properties for Refined QM/MM Models of Azurin. Inorg. Chem. 2021, 60, 7399–7412. 10.1021/acs.inorgchem.1c00640. 33939922 PMC8154437

[ref68] Vancoillie S.; Malmqvist P.-Å.; Pierloot K. Calculation of EPR g Tensors for Transition-Metal Complexes Based on Multiconfigurational Perturbation Theory (CASPT2). ChemPhysChem 2007, 8, 1803–1815. 10.1002/cphc.200700128. 17647251

[ref69] Sayfutyarova E. R.; Chan G. K.-L. Electron paramagnetic resonance g-tensors from state interaction spin-orbit coupling density matrix renormalization group. J. Chem. Phys. 2018, 148, 184103 10.1063/1.5020079. 29764129

[ref70] Singh S. K.; Atanasov M.; Neese F. Challenges in Multireference Perturbation Theory for the Calculations of the g-Tensor of First-Row Transition-Metal Complexes. J. Chem. Theory Comput. 2018, 14, 4662–4677. 10.1021/acs.jctc.8b00513. 30067364

[ref71] Zhou C.; Wu D.; Gagliardi L.; Truhlar D. G. Calculation of the Zeeman Effect for Transition-Metal Complexes by Multiconfiguration Pair-Density Functional Theory. J. Chem. Theory Comput. 2021, 17, 5050–5063. 10.1021/acs.jctc.1c00208. 34338523

[ref72] Scholl H. J.; Huettermann J. ESR and ENDOR of copper(II) complexes with nitrogen donors: probing parameters for prosthetic group modeling of superoxide dismutase. J. Phys. Chem. 1992, 96, 9684–9691. 10.1021/j100203a023.

[ref73] Suzuki Y.; Fujii S.; Tominaga T.; Yoshimoto T.; Yoshimura T.; Kamada H. The origin of an EPR signal observed in dithiocarbamate-loaded tissues: Copper(II)-dithiocarbamate complexes account for the narrow hyperfine lines. Biochim. Biophys. Acta 1997, 1335, 242–245. 10.1016/s0304-4165(97)00027-5. 9202186

[ref74] Ritterskamp N.; Sharples K.; Richards E.; Folli A.; Chiesa M.; Platts J. A.; Murphy D. M. Understanding the Coordination Modes of [Cu(acac)_2_(imidazole)_*n*=1,2_] Adducts by EPR, ENDOR, HYSCORE, and DFT Analysis. Inorg. Chem. 2017, 56, 11862–11875. 10.1021/acs.inorgchem.7b01874. 28933856

[ref75] Carter E.; Hazeland E. L.; Murphy D. M.; Ward B. D. Structure, EPR/ENDOR and DFT characterisation of a [Cu^II^(en)_2_](OTf)_2_ complex. Dalton Trans. 2013, 42, 15088–15096. 10.1039/c3dt51694f. 24000097

[ref76] Bader K.; Dengler D.; Lenz S.; Endeward B.; Jiang S.-D.; Neugebauer P.; van Slageren J. Room temperature quantum coherence in a potential molecular qubit. Nat. Commun. 2014, 5, 5304 10.1038/ncomms6304. 25328006

[ref77] Antholine W. E.; Basosit R.; Hyde J. S.; Lyman S.; Petering D. H. Immobile- and mobile-phase ESR spectroscopy of copper complexes: studies on biologically interesting bis(thiosemicarbazonato)copper(II) chelates. Inorg. Chem. 1984, 23, 3543–3548. 10.1021/ic00190a022.

[ref78] Kuźniarska-Biernacka I.; Kurzak K.; Kurzak B.; Jezierska J. Spectrochemical Properties of Noncubical Transition Metal Complexes in Solutions. XV. Solution Properties of bis(Salicylideneaniline)Copper(II). J. Solution Chem. 2003, 32, 719–741. 10.1023/b:josl.0000002991.55538.1.

[ref79] García-Tojal J.; García-Orad A.; Serra J. L.; Pizarro J. L.; Lezama L.; Arriortua M. I.; Rojo T. Synthesis and spectroscopic properties of copper(II) complexes derived from thiophene-2-carbaldehyde thiosemicarbazone. Structure and biological activity of [Cu(C_6_H_6_N_3_S_2_)_2_]. J. Inorg. Biochem. 1999, 75, 45–54. 10.1016/s0162-0134(99)00031-8. 10402676

[ref80] Singh N.; Niklas J.; Poluektov O.; Van Heuvelen K. M.; Mukherjee A. Mononuclear nickel (II) and copper (II) coordination complexes supported by bispicen ligand derivatives: Experimental and computational studies. Inorg. Chim. Acta 2017, 455, 221–230. 10.1016/j.ica.2016.09.001.

[ref81] Sarkar S.; Sen S.; Dey S.; Zangrando E.; Chattopadhyay P. Coordination behavior of 3,4-bis(2-pyridylmethylthio)toluene with copper(II) ions: Synthesis, structural characterization and reactivity, and DNA binding study of the dinuclear copper(II) complex. Polyhedron 2010, 29, 3157–3163. 10.1016/j.poly.2010.08.005.

[ref82] Ananth K. M.; Kanthimathi M.; Nair B. U. An e.p.r. study of tetradentate Schiff base copper complexes with an N–(CH_2_)_*n*_–N, *n* = 3–6 backbone. Transition Met. Chem. 2001, 26, 333–338. 10.1023/a:1007169527677.

[ref83] Comba P.; Hambley T. W.; Lawrance G. A.; Martin L. L.; Renold P.; Váarnagy K. Template syntheses of chiral tetradentate ligands derived from L-amino acids. Structural and spectroscopic characterization of the free ligands and of their copper(II) complexes. J. Chem. Soc., Dalton Trans. 1991, 277–283. 10.1039/dt9910000277.

[ref84] Bhadbhade M. M.; Srinivas D. Effects on molecular association, chelate conformation, and reactivity toward substitution in copper Cu(5-X-salen) complexes, salen2- = N,N’-ethylenebis(salicylidenaminato), X = H, CH_3_O, and Cl: synthesis, x-ray structures, and EPR investigations. Inorg. Chem. 1993, 32, 5458–5466. 10.1021/ic00076a010.

[ref85] Balaiah B.; Sastry B. A.; Chary M. N.; Ponticelli G.; Massacesi M. IR, EPR and optical absorption studies of some 2,2’-bipyridine complexes of Copper(II). J. Mol. Struct. 1982, 78, 289–297. 10.1016/0022-2860(82)80015-x.

[ref86] Glass R. S.; Steffen L. K.; Swanson D. D.; Wilson G. S.; de Gelder R.; de Graaff R. A. G.; Reedijk J. Bis(trithiacyclononane)metal(II) compounds and Jahn-Teller distortions from octahedral geometry, electrochemistry, spectroscopy, and crystal structures of the copper bis(tetrafluoroborate) bis(acetonitrile) complex at 177 K and the cadmium bis(tetrafluoroborate) and copper bis(tetrafluoroborate) bis(nitromethane) complexes at 300 K. Inorg. Chim. Acta 1993, 207, 241–252. 10.1016/s0020-1693(00)90716-3.

[ref87] Kaupp M.; Reviakine R.; Malkina O. L.; Arbuznikov A.; Schimmelpfennig B.; Malkin V. G. Calculation of electronic g-tensors for transition metal complexes using hybrid density functionals and atomic meanfield spin-orbit operators. J. Comput. Chem. 2002, 23, 794–803. 10.1002/jcc.10049. 12012356

[ref88] Fritscher J.; Hrobárik P.; Kaupp M. Computational Studies of Electron Paramagnetic Resonance Parameters for Paramagnetic Molybdenum Complexes. 1. Method Validation on Small and Medium-Sized Systems. J. Phys. Chem. B 2007, 111, 4616–4629. 10.1021/jp070638y. 17408258

[ref89] Fritscher J.; Hrobárik P.; Kaupp M. Computational Studies of EPR Parameters for Paramagnetic Molybdenum Complexes. II. Larger MoV Systems Relevant to Molybdenum Enzymes. Inorg. Chem. 2007, 46, 8146–8161. 10.1021/ic070341e. 17725345

[ref90] Gohr S.; Hrobárik P.; Repiský M.; Komorovský S.; Ruud K.; Kaupp M. Four-Component Relativistic Density Functional Theory Calculations of EPR g- and Hyperfine-Coupling Tensors Using Hybrid Functionals: Validation on Transition-Metal Complexes with Large Tensor Anisotropies and Higher-Order Spin–Orbit Effects. J. Phys. Chem. A 2015, 119, 12892–12905. 10.1021/acs.jpca.5b10996. 26636191

[ref91] Wodyński A.; Kaupp M. Density Functional Calculations of Electron Paramagnetic Resonance g- and Hyperfine-Coupling Tensors Using the Exact Two-Component (X2C) Transformation and Efficient Approximations to the Two-Electron Spin–Orbit Terms. J. Phys. Chem. A 2019, 123, 5660–5672. 10.1021/acs.jpca.9b03979. 31184482

[ref92] Courtade G.; Ciano L.; Paradisi A.; Lindley P. J.; Forsberg Z.; Sørlie M.; Wimmer R.; Davies G. J.; Eijsink V. G. H.; Walton P. H.; Aachmann F. L. Mechanistic basis of substrate–O_2_ coupling within a chitin-active lytic polysaccharide monooxygenase: An integrated NMR/EPR study. Proc. Natl. Acad. Sci. U.S.A. 2020, 117, 19178 10.1073/pnas.2004277117. 32723819 PMC7431007

[ref93] Ganyushin D.; Neese F. A fully variational spin-orbit coupled complete active space self-consistent field approach: Application to electron paramagnetic resonance g-tensors. J. Chem. Phys. 2013, 138, 104113 10.1063/1.4793736. 23514471

[ref94] Schattenberg C. J.; Maier T. M.; Kaupp M. Lessons from the Spin-Polarization/Spin-Contamination Dilemma of Transition-Metal Hyperfine Couplings for the Construction of Exchange-Correlation Functionals. J. Chem. Theory Comput. 2018, 14, 5653–5672. 10.1021/acs.jctc.8b00597. 30299950

[ref95] Addison A. W.; Rao T. N.; Reedijk J.; van Rijn J.; Verschoor G. C. Synthesis, structure, and spectroscopic properties of copper(II) compounds containing nitrogen–sulphur donor ligands; the crystal and molecular structure of aqua[1,7-bis(N-methylbenzimidazol-2′-yl)-2,6-dithiaheptane]copper(II) perchlorate. J. Chem. Soc., Dalton Trans. 1984, 1349–1356. 10.1039/dt9840001349.

