# Supplementary material for: Decoding the Ambiguous Electron Paramagnetic Resonance Signals in the Lytic Polysaccharide Monooxygenase from Photorhabdus luminescens
Source: Inorg Chem. 2022 May 12;61(20):8022–35. doi: 10.1021/acs.inorgchem.2c00766 (PMC9131454; doi:10.1021/acs.inorgchem.2c00766)
Supplement: Supplementary file 1 — ic2c00766_si_001.pdf [file ic2c00766_si_001.pdf]

SUPPORTING INFORMATION

for

Decoding the ambiguous EPR signals in the lytic polysaccharide monooxygenase from *Photorhabdus luminescens*

Rogelio J. Gómez-Piñeiro,[1] Maria Drosou,[2] Sylvain Bertaina,[3] Christophe Decroos,[1]
A. Jalila Simaan,[1] Dimitrios A. Pantazis,[4*] Maylis Orio[1*]

[1] *Aix Marseille Univ, CNRS, Centrale Marseille, iSm2, Marseille, France.*
[2] *Inorganic Chemistry Laboratory, National and Kapodistrian University of Athens, Panepistimiopolis, Zografou 15771, Greece.*
[3] *Aix-Marseille Université, CNRS, IM2NP UMR 7334, Marseille, France.*
[4] *Max-Planck-Institut für Kohlenforschung, Kaiser-Wilhelm-Platz 1, 45470 Mülheim an der Ruhr, Germany.*

E-mail: dimitrios.pantazis@kofo.mpg.de
E-mail: maylis.orio@univ-amu.fr

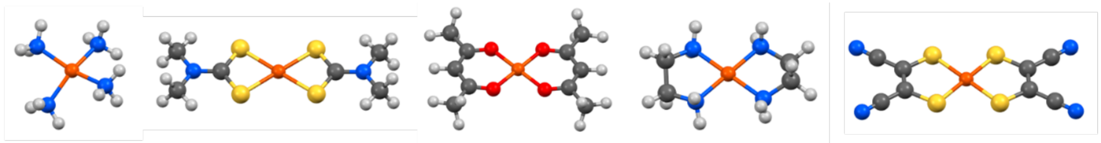

(1) $[Cu(NH_3)_4]^{2+}$ **(2)** $[Cu(dtc)_2]$ **(3)** $[Cu(acac)_2]$ **(4)** $[Cu(en)_2]^{2+}$ **(5)** $[Cu(mnt)_2]^{2+}$

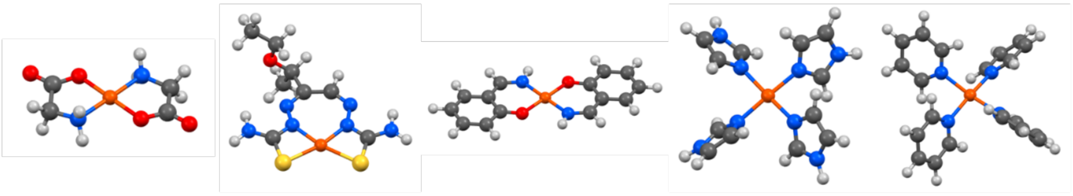

(6) $[Cu(gly)_2]$ **(7)** [Cu(kts)] **(8)** $[Cu(sac)_2]$ **(9)** $[Cu(im)_4]^{2+}$ **(10)** $[Cu(py)_4]^{2+}$

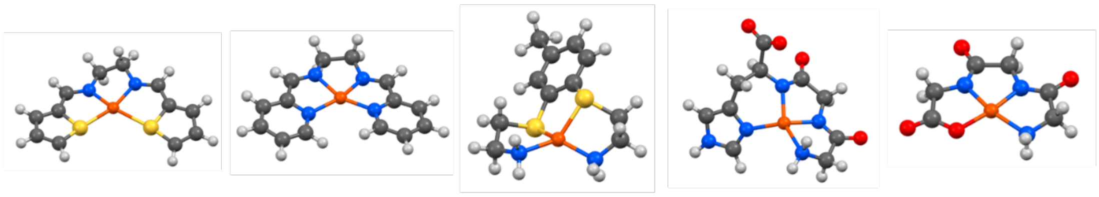

(11) $[Cu(eta)]^{2+}$ **(12)** $[Cu(epa)]^{2+}$ **(13)** $[Cu(atpt)]^{2+}$ **(14)** $[Cu(GGH)]^{-}$ **(15)** $[Cu(GGG)]^{-}$

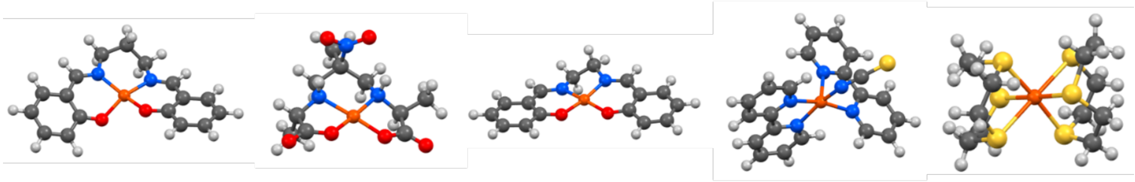

(16) [Cu(salpn)] **(17)** [Cu(S,S-mnpala)] **(18)** [Cu(salen)] **(19)** $[Cu(bipy)_2(NCS)]^{+}$ **(20)** $[Cu(ttcn)_2]^{2+}$

Figure S1. Set of the copper(II) complexes considered in this work. Ligand abbreviations: dtc=dimethyl-dithiocarbamate, acac=acetylacetone; en=ethylenediamine; mnt=maleonitriledithiolate; gly=glycine; kts=2-keto-3-ethoxybutyraldehyde-bis(thiosemicarbazone); sac=salicylaldehyde imine; im=imidazole; py=pyridine; epa=N,N’-ethylenebis(pyridine-2-aldimine); eta=N,N’-ethylenebis(thiophene-2-aldimine); atpt=3,4-bis(3-amino-1-thiopropyl) toluene; GGH=glycine-glycine-histidine; GGG=glycine-glycine-glycine; salpn = N,N’-bis(salicylidene)-1,2-propanediamine; (S,S)-mnpala=2,5,8-trimethyl-5-nitro-3,7-diazanonanedioate; salen = bis(saliculidene)ethylene-diamine; bipy=2,2’-bipyridine; ttcn=1,4,7-trithiacyclononane. Adapted with permission from Chem. Phys. Chem. 2020, 21, 2667–2679.[1]

Table S1. Comparison of key structural parameters (bond lengths in Å, angles in degrees) of the DFT model **A1** optimized with the BP86 and B3LYP functionals. The parameter τ is a geometry index that quantifies the structural distortion of 4- and 5-coordinate complexes (defined in Scheme S1); for 4-coordinate complexes[2] τ = 1.00 means perfect tetrahedral geometry and τ = 0.00 means zero distortion from the square planar geometry, whereas for 5-coordinate complexes[3] τ = 1.00 means perfect trigonal-bipyramidal geometry and τ = 0.00 means zero distortion from the square-pyramidal geometry. In model **A1**, angle α corresponds to the N2-Cu-O2 angle and β to the N1-Cu-N3 angle.

Functional	Cu-N1	Cu-N2	Cu-N3	Cu-O1	Cu-O2	α	β	τ
BP86	1.958	2.143	1.979	2.332	2.363	134.0	174.2	0.67
B3LYP	1.971	2.138	1.995	2.302	2.335	134.4	174.3	0.67

5-member coordination sphere

4-member coordination sphere

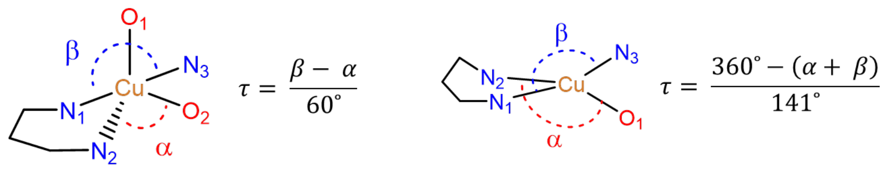

Scheme S1. Geometry index (τ) definition. See Figure 2 for assignment of specific atoms in the DFT models.

Table S2. Calculated g-factors, g-shifts ($g^s = (g_{calc.} - g_e)\times10^3$, with $g_e = 2.0023$), and corresponding absolute percent differences from experiment ($\mathrm{APD}(g^s) = \left|\frac{g^s_{calc.} - g^s_{exp.}}{g^s_{exp.}}\right| \times 100$) for $[Cu(NH_3)_4]^{2+}$ obtained with PBE0 and B3PW91, with various basis sets for Cu that were previously considered for calculations of Cu hyperfine coupling constants.[1] The def2-TZVP basis sets were used for all other atoms. The results confirm that the Cu basis set used for the hyperfine coupling constants (aug-cc-pVTZ-Jmod) provides converged results also for the g-tensor.

**PBE0**	g_x	g_y	g_z	$g_\perp{}^s$	$g_\parallel{}^s$	$g_{iso}{}^s$	APD($g_\perp{}^s$)	APD($g_\parallel{}^s$)	APD($g_{iso}{}^s$)
aug-cc-pVTZ	2.053	2.053	2.189	50.7	186.7	96.0	13.4	21.8	12.2
aug-cc-pVTZ-J	2.053	2.053	2.189	50.7	186.7	96.0	13.4	21.8	12.2
aug-cc-pVTZ-Jmod	2.053	2.053	2.189	50.7	187.7	96.0	13.4	21.8	12.2
aug-cc-pVTZ-Junc	2.053	2.053	2.189	50.7	186.7	96.0	13.4	21.8	12.2
aug-cc-pwCVTZ	2.053	2.053	2.189	50.7	186.7	96.0	13.4	21.8	12.2
cc-pwCVTZ	2.053	2.053	2.188	50.7	185.7	95.7	13.4	22.2	12.5
CP(PPP)	2.053	2.053	2.184	50.7	181.7	94.3	13.4	23.9	13.7
Def2-TZVP	2.053	2.052	2.185	49.7	182.7	94.0	11.2	23.5	14.0
Def2-QZVPP	2.053	2.053	2.188	50.7	185.7	95.7	13.4	22.2	12.5
**B3PW91**	g_x	g_y	g_z	$g_\perp{}^s$	$g_\parallel{}^s$	$g_{iso}{}^s$	APD($g_\perp{}^s$)	APD($g_\parallel{}^s$)	APD($g_{iso}{}^s$)
aug-cc-pVTZ	2.048	2.048	2.171	45.7	168.7	86.7	2.2	29.3	20.7
aug-cc-pVTZ-J	2.048	2.048	2.172	45.7	169.7	87.0	2.2	28.9	20.4
aug-cc-pVTZ-Jmod	2.048	2.048	2.172	45.7	169.7	87.0	2.2	28.9	20.4
aug-cc-pVTZ-Junc	2.048	2.048	2.172	45.7	169.7	87.0	2.2	28.9	20.4
aug-cc-pwCVTZ	2.045	2.045	2.171	42.7	168.7	84.7	4.5	29.3	22.6
cc-pwCVTZ	2.048	2.048	2.171	45.7	168.7	86.7	2.2	29.3	20.7
CP(PPP)	2.048	2.048	2.166	45.7	163.7	85.0	2.2	31.4	22.3
Def2-TZVP	2.048	2.048	2.166	45.7	163.7	85.0	2.2	31.4	22.3
Def2-QZVPP	2.048	2.048	2.170	45.7	167.7	86.4	2.2	29.7	21.0
Exp.	2.047	2.047	2.241	44.7	238.7	109.3			

Table S3. Comparison between non-relativistic results and those obtained with the scalar relativistic Hamiltonians DKH2 and ZORA. Experimental values are listed first for convenience. Calculated g-factors, g-shifts (g^s), and corresponding absolute percent differences from experiment (APD) for the first six complexes of the benchmark set, compared to experimental values. The calculations were performed with the B3PW91 functional, in combination with the aug-cc-pVTZ-Jmod basis set for Cu and the def2-TZVP or DKH/ZORA-def2-TZVP basis sets for ligand atoms. No systematic improvement is observed with the use of scalar relativistic Hamiltonians.

	g_x	g_y	g_z	$g_\perp^s$	$g_\parallel^s$	APD($g_\perp^s$)	APD($g_\parallel^s$)
**Exp.**							
**1**	2.047	2.047	2.241	44.7	238.7		
**2**	2.025	2.025	2.085	22.7	82.7		
**3**	2.060	2.060	2.285	57.7	282.7		
**4**	2.040	2.046	2.202	40.7	199.7		
**5**	2.023	2.023	2.093	20.7	90.7		
**6**	2.052	2.052	2.267	49.7	264.7		
**Non-relativistic**							
**1**	2.048	2.048	2.172	45.8	169.4	2.6	29.0
**2**	2.020	2.027	2.073	21.4	71.1	5.6	14.0
**3**	2.047	2.051	2.178	47.1	175.5	18.3	37.9
**4**	2.039	2.039	2.137	36.9	134.4	9.3	32.7
**5**	2.017	2.017	2.054	14.8	51.8	28.6	42.9
**6**	2.034	2.051	2.149	39.8	146.8	19.9	44.5
**DKH2**							
**1**	2.049	2.049	2.175	46.8	172.3	4.6	27.8
**2**	2.022	2.028	2.079	22.6	76.7	0.7	7.2
**3**	2.048	2.052	2.180	47.8	177.5	17.1	37.2
**4**	2.040	2.040	2.140	37.7	137.4	7.3	31.2
**5**	2.017	2.018	2.056	15.2	54.0	26.4	40.4
**6**	2.034	2.051	2.152	40.5	149.3	18.4	43.6
**ZORA**							
**1**	2.049	2.049	2.175	47.0	172.3	5.1	27.8
**2**	2.022	2.028	2.079	22.8	76.8	0.4	7.1
**3**	2.048	2.052	2.180	48.0	177.3	16.8	37.3
**4**	2.040	2.040	2.140	38.0	137.4	6.7	31.2
**5**	2.018	2.018	2.057	15.5	54.3	25.1	40.1
**6**	2.035	2.051	2.152	40.8	149.3	18.0	43.6

Detailed results for the benchmark set of Cu complexes

Table S4. Calculated g-tensor components, differences (D) from the experimental values of the Δg parameter, per mille differences (‰PD) from the experimental values of the $g_{x,y}$ ($g_{\perp}$) and g_z ($g_{\parallel}$) components, percent differences (PD) from the experimental values of the g_z^s ($g_{\parallel}^s$) and Δg values, and computed Löwdin atomic spin populations ρ_s on Cu for the set of 20 Cu(II) complexes, obtained with the **PBE** functional.

Comp.	g_x	g_y	g_z	Δg	$D(\Delta g)^a$	‰PD($g_{x,y}$)[b,c]	‰PD(g_z)	%PD(g_z^s)[d,e]	%PD(Δg)	ρ_s(Cu)
**1**	2.034	2.034	2.121	0.086	-0.108	-6.3	-53.8	-50.5	-55.4	0.583
**2**	2.014	2.023	2.046	0.027	-0.033	-3.0	-18.5	-46.7	-54.2	0.366
**3**	2.032	2.036	2.120	0.086	-0.139	-12.7	-72.2	-58.4	-61.7	0.536
**4**	2.028	2.028	2.094	0.065	-0.094	-7.2	-49.1	-54.2	-58.8	0.528
**5**	2.012	2.015	2.032	0.018	-0.052	-4.6	-29.2	-67.3	-73.9	0.363
**6**	2.025	2.033	2.101	0.072	-0.143	-11.2	-73.1	-62.6	-66.4	0.523
**7**	2.026	2.027	2.083	0.056	-0.054	-1.6	-26.7	-41.5	-49.1	0.462
**8**	2.026	2.034	2.101	0.071	-0.119	-9.9	-62.1	-58.6	-62.6	0.547
**9**	2.031	2.031	2.113	0.082	-0.133	-7.7	-65.9	-57.4	-62.0	0.578
**10**	2.030	2.031	2.107	0.077	-0.133	-11.0	-68.7	-59.7	-63.4	0.530
**11**	2.012	2.026	2.082	0.063	-0.067	-5.5	-36.2	-49.5	-51.6	0.421
**12**	2.030	2.031	2.101	0.071	-0.089	-10.9	-50.5	-53.0	-55.9	0.517
**13**	2.021	2.024	2.082	0.060	-0.075	-37.0	-68.3	-65.6	-55.6	0.393
**14**	2.019	2.021	2.068				-48.1	-61.3		0.355
**15**	2.023	2.025	2.088				-51.9	-57.2		0.506
**16**	2.027	2.033	2.105	0.075	-0.126	-14.5	-68.8	-60.1	-62.6	0.523
**17**	2.026	2.029	2.100	0.072	-0.108	-15.6	-62.7	-59.1	-60.1	0.504
**18**	2.027	2.029	2.099	0.071	-0.069	-11.6	-42.4	-49.0	-49.4	0.535
**19**	2.034	2.056	2.113	0.067	-0.104	-20.5	-64.8	-57.0	-60.7	0.493
**20**	2.016	2.023	2.064	0.045	-0.027	-12.5	-24.9	-46.0	-37.7	0.389

[a] $D(\Delta g) = \Delta g^{calc} - \Delta g^{exp}$

[b] $g_{x,y} = g_{\perp} = (g_x + g_y)/2$

[c] $‰PD(g_{x,y}) = \left(\frac{g_{x,y}^{calc} - g_{x,y}^{exp}}{g_{x,y}^{exp}}\right) \cdot 1000$

[d] $g_z^s = g_z - g_e$, $g_e = 2.002319$

[e] $\%PD(g_z{}^s) = \left(\frac{g_z^{s,calc} - g_z^{s,exp}}{g_z^{s,exp}}\right) \cdot 100$

Table S5. Calculated g-tensor components, differences (D) from the experimental values of the Δg parameter, per mille differences (‰PD) from the experimental values of the $g_{x,y}$ ($g_{\perp}$) and g_z ($g_{\parallel}$) components, percent differences (PD) from the experimental values of the g_z^{s} ($g_{\parallel}^{s}$) and Δg values, and computed Löwdin atomic spin populations ρ_s on Cu for the set of 20 Cu(II) complexes, obtained with the **TPSS** functional.

Comp.	g_x	g_y	g_z	Δg	D(Δg)	‰PD($g_{x,y}$)	‰PD(g_z)	%PD(g_z^{s})	%PD(Δg)	ρ_s(Cu)
**1**	2.033	2.033	2.113	0.079	-0.115	-6.7	-57.2	-53.7	-59.1	0.607
**2**	2.015	2.023	2.049	0.029	-0.031	-2.8	-17.4	-43.9	-51.0	0.387
**3**	2.032	2.035	2.113	0.080	-0.145	-12.9	-75.3	-60.8	-64.6	0.564
**4**	2.028	2.028	2.090	0.062	-0.097	-7.3	-50.8	-56.0	-61.0	0.551
**5**	2.013	2.015	2.033	0.019	-0.051	-4.5	-28.5	-65.8	-72.1	0.382
**6**	2.025	2.033	2.097	0.068	-0.147	-11.3	-75.1	-64.3	-68.4	0.551
**7**	2.027	2.028	2.082	0.055	-0.055	-1.4	-27.1	-42.1	-50.0	0.486
**8**	2.026	2.033	2.097	0.067	-0.123	-10.0	-64.0	-60.3	-64.7	0.573
**9**	2.031	2.031	2.107	0.076	-0.139	-7.9	-68.4	-59.6	-64.5	0.603
**10**	2.030	2.031	2.103	0.073	-0.137	-11.0	-70.5	-61.2	-65.2	0.557
**11**	2.018	2.028	2.082	0.059	-0.071	-3.4	-35.9	-49.2	-54.4	0.460
**12**	2.030	2.031	2.098	0.067	-0.093	-10.9	-52.1	-54.8	-58.2	0.545
**13**	2.022	2.024	2.082	0.059	-0.076	-36.8	-68.6	-65.9	-56.3	0.416
**14**	2.019	2.022	2.068				-48.2	-61.3		0.375
**15**	2.023	2.025	2.085				-53.2	-58.6		0.531
**16**	2.028	2.033	2.101	0.071	-0.130	-14.5	-70.8	-61.9	-64.8	0.550
**17**	2.026	2.029	2.096	0.068	-0.112	-15.6	-64.5	-60.7	-62.3	0.531
**18**	2.027	2.029	2.095	0.067	-0.073	-11.6	-44.2	-51.1	-52.2	0.562
**19**	2.035	2.051	2.108	0.066	-0.105	-21.7	-66.7	-58.7	-61.7	0.523
**20**	2.018	2.024	2.065	0.044	-0.028	-11.9	-24.5	-45.2	-38.3	0.414

Table S6. Calculated *g*-tensor components, differences (D) from the experimental values of the Δ*g* parameter, per mille differences (‰PD) from the experimental values of the $g_{x,y}$ ($g_{\perp}$) and g_z ($g_{\parallel}$) components, percent differences (PD) from the experimental values of the g_z^s ($g_{\parallel}^s$) and Δ*g* values, and computed Löwdin atomic spin populations ρ_s on Cu for the set of 20 Cu(II) complexes, obtained with the **TPSSh** functional.

Comp.	g_x	g_y	g_z	Δ*g*	D(Δ*g*)	‰PD($g_{x,y}$)	‰PD(g_z)	%PD(g_z^s)	%PD(Δ*g*)	ρ_s(Cu)
**1**	2.039	2.039	2.133	0.094	-0.100	-3.8	-48.2	-45.2	-51.7	0.647
**2**	2.018	2.025	2.061	0.039	-0.021	-1.7	-11.5	-29.0	-34.4	0.413
**3**	2.038	2.042	2.136	0.096	-0.129	-9.7	-65.4	-52.9	-57.5	0.621
**4**	2.033	2.033	2.108	0.075	-0.084	-5.0	-42.8	-47.2	-53.0	0.590
**5**	2.015	2.016	2.044	0.028	-0.042	-3.7	-23.6	-54.5	-59.9	0.413
**6**	2.029	2.040	2.116	0.081	-0.134	-8.6	-66.7	-57.1	-62.1	0.604
**7**	2.032	2.032	2.100	0.068	-0.042	0.8	-18.8	-29.2	-38.0	0.528
**8**	2.032	2.039	2.116	0.080	-0.110	-7.2	-55.5	-52.3	-57.7	0.623
**9**	2.036	2.036	2.126	0.090	-0.125	-5.3	-59.9	-52.2	-58.0	0.644
**10**	2.035	2.036	2.123	0.088	-0.122	-8.5	-61.8	-53.6	-58.2	0.602
**11**	2.032	2.037	2.103	0.069	-0.061	2.1	-26.2	-35.9	-46.9	0.536
**12**	2.036	2.037	2.118	0.082	-0.078	-7.8	-42.7	-44.9	-49.0	0.596
**13**	2.026	2.027	2.097	0.071	-0.064	-34.9	-61.6	-59.2	-47.6	0.455
**14**	2.023	2.025	2.083				-41.3	-52.6		0.422
**15**	2.027	2.031	2.101				-46.0	-50.7		0.579
**16**	2.034	2.039	2.122	0.086	-0.115	-11.4	-61.3	-53.6	-57.3	0.605
**17**	2.032	2.035	2.115	0.082	-0.098	-13.0	-55.6	-52.4	-54.4	0.584
**18**	2.033	2.035	2.115	0.081	-0.059	-8.8	-35.4	-40.8	-42.5	0.613
**19**	2.044	2.050	2.133	0.086	-0.085	-19.6	-55.6	-48.9	-49.5	0.591
**20**	2.023	2.028	2.080	0.055	-0.017	-9.6	-17.5	-32.3	-24.1	0.452

Table S7. Calculated g-tensor components, differences (D) from the experimental values of the Δg parameter, per mille differences (‰PD) from the experimental values of the $g_{x,y}$ ($g_{\perp}$) and g_z ($g_{\parallel}$) components, percent differences (PD) from the experimental values of the g_z^s ($g_{\parallel}^s$) and Δg values, and computed Löwdin atomic spin populations ρ_s on Cu for the set of 20 Cu(II) complexes, obtained with the **B3LYP** functional.

Comp.	g_x	g_y	g_z	Δg	D(Δg)	‰PD($g_{x,y}$)	‰PD(g_z)	%PD(g_z^s)	%PD(Δg)	ρ_s(Cu)
**1**	2.048	2.048	2.170	0.122	-0.072	0.3	-31.7	-29.8	-37.0	0.662
**2**	2.020	2.027	2.072	0.048	-0.012	-0.7	-6.3	-15.8	-19.3	0.413
**3**	2.047	2.051	2.176	0.127	-0.098	-5.3	-47.6	-38.5	-43.5	0.653
**4**	2.039	2.039	2.135	0.096	-0.063	-2.1	-30.4	-33.5	-39.4	0.605
**5**	2.017	2.017	2.055	0.038	-0.032	-2.9	-18.2	-42.0	-45.9	0.424
**6**	2.033	2.050	2.148	0.106	-0.109	-5.0	-52.5	-45.0	-50.6	0.630
**7**	2.036	2.037	2.123	0.086	-0.024	3.2	-8.0	-12.4	-21.4	0.543
**8**	2.039	2.046	2.147	0.104	-0.086	-3.6	-41.5	-39.1	-45.1	0.649
**9**	2.043	2.043	2.159	0.116	-0.099	-2.0	-45.6	-39.7	-46.1	0.661
**10**	2.042	2.042	2.155	0.113	-0.097	-5.3	-47.8	-41.5	-46.4	0.622
**11**	2.039	2.045	2.129	0.087	-0.043	5.9	-14.2	-19.5	-32.9	0.553
**12**	2.044	2.045	2.151	0.106	-0.054	-4.2	-28.2	-29.6	-33.6	0.618
**13**	2.030	2.031	2.117	0.086	-0.049	-33.1	-52.9	-50.8	-36.1	0.466
**14**	2.027	2.030	2.104				-31.9	-40.6		0.454
**15**	2.032	2.037	2.125				-34.9	-38.5		0.601
**16**	2.043	2.047	2.158	0.113	-0.088	-7.3	-45.6	-39.9	-43.9	0.634
**17**	2.039	2.042	2.147	0.107	-0.073	-9.6	-41.3	-39.0	-40.4	0.610
**18**	2.040	2.042	2.146	0.105	-0.035	-5.3	-21.0	-24.3	-25.2	0.640
**19**	2.055	2.058	2.174	0.118	-0.053	-15.0	-37.6	-33.1	-31.3	0.628
**20**	2.027	2.031	2.097	0.068	-0.004	-7.7	-9.5	-17.6	-6.0	0.460

Table S8. Calculated g-tensor components, differences (D) from the experimental values of the Δg parameter, per mille differences (‰PD) from the experimental values of the $g_{x,y}$ ($g_{\perp}$) and g_z ($g_{\parallel}$) components, percent differences (PD) from the experimental values of the g_z^s ($g_{\parallel}^s$) and Δg values, and computed Löwdin atomic spin populations ρ_s on Cu for the set of 20 Cu(II) complexes, obtained with the **PBE0** functional.

Comp.	g_x	g_y	g_z	Δg	D(Δg)	‰PD($g_{x,y}$)	‰PD(g_z)	%PD(g_z^s)	%PD(Δg)	ρ_s(Cu)
**1**	2.053	2.053	2.189	0.136	-0.058	2.9	-23.1	-21.6	-29.7	0.690
**2**	2.023	2.029	2.086	0.060	0.000	0.6	0.4	1.0	-0.5	0.441
**3**	2.053	2.056	2.197	0.142	-0.083	-2.7	-38.7	-31.3	-36.9	0.686
**4**	2.043	2.043	2.152	0.109	-0.050	0.1	-22.8	-25.1	-31.6	0.634
**5**	2.018	2.019	2.062	0.043	-0.027	-2.1	-14.8	-34.2	-38.2	0.447
**6**	2.037	2.056	2.165	0.119	-0.096	-2.7	-44.9	-38.4	-44.8	0.662
**7**	2.041	2.042	2.141	0.100	-0.010	5.6	0.7	1.0	-9.1	0.577
**8**	2.044	2.050	2.163	0.116	-0.074	-1.5	-34.5	-32.5	-39.1	0.677
**9**	2.047	2.047	2.176	0.129	-0.086	0.1	-38.1	-33.1	-40.1	0.687
**10**	2.046	2.047	2.173	0.126	-0.084	-3.1	-39.9	-34.6	-39.9	0.651
**11**	2.046	2.052	2.149	0.100	-0.030	9.5	-4.9	-6.8	-23.0	0.589
**12**	2.049	2.050	2.169	0.120	-0.040	-1.6	-19.7	-20.7	-25.1	0.650
**13**	2.035	2.035	2.135	0.100	-0.035	-30.9	-44.6	-42.8	-25.7	0.503
**14**	2.032	2.036	2.121				-23.8	-30.3		0.502
**15**	2.035	2.041	2.139				-28.7	-31.6		0.630
**16**	2.048	2.052	2.177	0.126	-0.075	-4.7	-37.2	-32.5	-37.1	0.667
**17**	2.044	2.046	2.166	0.121	-0.059	-7.2	-33.0	-31.1	-32.8	0.644
**18**	2.045	2.046	2.162	0.116	-0.024	-3.1	-13.7	-15.8	-16.9	0.669
**19**	2.060	2.066	2.199	0.136	-0.035	-12.0	-26.5	-23.3	-20.4	0.673
**20**	2.032	2.036	2.113	0.079	0.007	-5.4	-2.1	-3.9	9.2	0.495

Table S9. Calculated g-tensor components, differences (D) from the experimental values of the Δg parameter, per mille differences (‰PD) from the experimental values of the $g_{x,y}$ ($g_{\perp}$) and g_z ($g_{\parallel}$) components, percent differences (PD) from the experimental values of the g_z^{s} ($g_{\parallel}^{s}$) and Δg values, and computed Löwdin atomic spin populations ρ_s on Cu for the set of 20 Cu(II) complexes, obtained with the **BHandHLYP** functional.

Comp.	g_x	g_y	g_z	Δg	D(Δg)	‰PD($g_{x,y}$)	‰PD(g_z)	%PD(g_z^{s})	%PD(Δg)	ρ_s(Cu)
**1**	2.075	2.075	2.278	0.203	0.009	13.8	16.6	15.6	4.6	0.795
**2**	2.045	2.046	2.170	0.125	0.065	10.1	40.8	103.0	107.9	0.589
**3**	2.075	2.077	2.286	0.210	-0.015	7.8	0.5	0.4	-6.7	0.809
**4**	2.062	2.062	2.233	0.171	0.012	9.4	14.2	15.7	7.7	0.752
**5**	2.030	2.033	2.117	0.086	0.016	4.3	11.6	26.7	22.2	0.576
**6**	2.050	2.083	2.245	0.179	-0.036	6.9	-9.7	-8.3	-16.9	0.779
**7**	2.063	2.066	2.231	0.166	0.056	17.1	42.4	66.0	51.0	0.719
**8**	2.063	2.068	2.237	0.171	-0.019	7.6	-1.5	-1.4	-9.9	0.789
**9**	2.064	2.065	2.252	0.187	-0.028	8.5	-4.5	-3.9	-12.8	0.787
**10**	2.066	2.066	2.257	0.191	-0.019	6.2	-2.7	-2.3	-9.0	0.771
**11**	2.076	2.086	2.252	0.172	0.042	25.0	42.8	58.6	31.9	0.741
**12**	2.071	2.072	2.257	0.185	0.025	9.1	19.8	20.8	15.7	0.773
**13**	2.066	2.068	2.251	0.184	0.049	-15.5	7.4	7.1	36.4	0.690
**14**	2.059	2.071	2.230				26.0	33.1		0.724
**15**	2.051	2.062	2.208				2.8	3.1		0.747
**16**	2.072	2.075	2.264	0.191	-0.010	6.5	1.4	1.2	-5.1	0.793
**17**	2.066	2.066	2.252	0.186	0.006	2.9	5.4	5.1	3.4	0.773
**18**	2.065	2.065	2.237	0.173	0.033	6.1	20.7	23.9	23.4	0.784
**19**	2.084	2.097	2.300	0.210	0.039	1.2	18.3	16.1	22.7	0.811
**20**	2.062	2.064	2.211	0.148	0.076	8.9	44.5	82.1	105.4	0.668

Table S10. Calculated g-tensor components, differences (D) from the experimental values of the Δg parameter, per mille differences (‰PD) from the experimental values of the $g_{x,y}$ ($g_{\perp}$) and g_z ($g_{\parallel}$) components, percent differences (PD) from the experimental values of the g_z^s ($g_{\parallel}^s$) and Δg values, and computed Löwdin atomic spin populations ρ_s on Cu for the set of 20 Cu(II) complexes, obtained with the **B3PW91 (20%)** functional (i.e. 20% exact exchange).

Comp.	g_x	g_y	g_z	Δg	D(Δg)	‰PD($g_{x,y}$)	‰PD(g_z)	%PD(g_z^s)	%PD(Δg)	ρ_s(Cu)
**1**	2.048	2.048	2.172	0.124	-0.070	0.6	-30.9	-29.0	-36.3	0.663
**2**	2.020	2.027	2.073	0.050	-0.010	-0.6	-5.6	-14.0	-17.2	0.416
**3**	2.047	2.051	2.178	0.128	-0.097	-5.1	-46.9	-37.9	-42.9	0.652
**4**	2.039	2.039	2.137	0.097	-0.062	-1.8	-29.7	-32.7	-38.7	0.606
**5**	2.017	2.017	2.054	0.037	-0.033	-2.9	-18.6	-42.9	-47.1	0.424
**6**	2.034	2.051	2.149	0.107	-0.108	-4.8	-52.0	-44.5	-50.2	0.630
**7**	2.037	2.037	2.125	0.088	-0.022	3.5	-7.0	-10.9	-20.0	0.544
**8**	2.040	2.046	2.148	0.105	-0.085	-3.5	-41.2	-38.8	-44.8	0.648
**9**	2.043	2.043	2.161	0.117	-0.098	-1.8	-44.9	-39.1	-45.5	0.661
**10**	2.042	2.043	2.156	0.114	-0.096	-5.1	-47.2	-40.9	-45.8	0.622
**11**	2.040	2.046	2.131	0.088	-0.042	6.3	-13.3	-18.2	-32.0	0.555
**12**	2.044	2.046	2.152	0.107	-0.053	-3.9	-27.4	-28.8	-32.8	0.619
**13**	2.030	2.032	2.119	0.088	-0.047	-32.9	-51.9	-49.9	-34.8	0.470
**14**	2.027	2.031	2.104				-31.5	-40.1		0.455
**15**	2.032	2.037	2.126				-34.7	-38.2		0.601
**16**	2.043	2.048	2.159	0.114	-0.087	-7.1	-45.0	-39.4	-43.4	0.633
**17**	2.039	2.042	2.149	0.108	-0.072	-9.4	-40.6	-38.3	-39.8	0.610
**18**	2.040	2.042	2.147	0.105	-0.035	-5.2	-20.6	-23.8	-24.7	0.638
**19**	2.055	2.059	2.177	0.120	-0.051	-14.8	-36.4	-32.1	-30.1	0.629
**20**	2.027	2.032	2.098	0.068	-0.004	-7.6	-9.0	-16.5	-4.9	0.462

Table S11. Calculated g-tensor components, differences (D) from the experimental values of the Δg parameter, per mille differences (‰PD) from the experimental values of the $g_{x,y}$ ($g_{\perp}$) and g_z ($g_{\parallel}$) components, percent differences (PD) from the experimental values of the g_z^s ($g_{\parallel}^s$) and Δg values, and computed Löwdin atomic spin populations ρ_s on Cu for the set of 20 Cu(II) complexes, obtained with the **B3PW91 - 25%** functional.

Comp.	g_x	g_y	g_z	Δg	D(Δg)	‰PD($g_{x,y}$)	‰PD(g_z)	%PD(g_z^s)	%PD(Δg)	ρ_s(Cu)
**1**	2.053	2.053	2.188	0.136	-0.058	2.7	-23.5	-22.0	-30.0	0.686
**2**	2.023	2.029	2.084	0.058	-0.002	0.4	-0.6	-1.6	-3.4	0.435
**3**	2.052	2.056	2.196	0.142	-0.083	-2.8	-38.9	-31.4	-37.0	0.683
**4**	2.043	2.043	2.151	0.108	-0.051	-0.1	-23.2	-25.6	-32.0	0.630
**5**	2.018	2.019	2.061	0.043	-0.027	-2.2	-15.1	-34.8	-38.7	0.444
**6**	2.036	2.056	2.164	0.118	-0.097	-2.8	-45.2	-38.7	-45.0	0.658
**7**	2.040	2.041	2.140	0.099	-0.011	5.3	-0.1	-0.2	-10.1	0.571
**8**	2.044	2.050	2.162	0.115	-0.075	-1.6	-34.7	-32.7	-39.2	0.674
**9**	2.047	2.047	2.175	0.128	-0.087	-0.1	-38.3	-33.4	-40.3	0.684
**10**	2.046	2.046	2.172	0.126	-0.084	-3.3	-40.2	-34.9	-40.2	0.648
**11**	2.045	2.052	2.147	0.099	-0.031	9.0	-5.8	-7.9	-23.7	0.584
**12**	2.049	2.050	2.169	0.119	-0.041	-1.8	-20.1	-21.1	-25.5	0.647
**13**	2.034	2.035	2.133	0.099	-0.036	-31.2	-45.5	-43.7	-26.8	0.497
**14**	2.031	2.035	2.119				-24.6	-31.4		0.495
**15**	2.035	2.041	2.138				-29.0	-32.0		0.626
**16**	2.048	2.052	2.176	0.126	-0.075	-4.8	-37.5	-32.8	-37.3	0.663
**17**	2.044	2.046	2.165	0.120	-0.060	-7.4	-33.4	-31.4	-33.1	0.640
**18**	2.045	2.046	2.162	0.116	-0.024	-3.2	-13.9	-16.0	-17.0	0.666
**19**	2.060	2.065	2.198	0.135	-0.036	-12.2	-27.0	-23.8	-20.9	0.668
**20**	2.031	2.035	2.111	0.078	0.006	-5.7	-2.9	-5.3	7.7	0.489

Table S12. Calculated g-tensor components, differences (D) from the experimental values of the Δg parameter, per mille differences (‰PD) from the experimental values of the $g_{x,y}$ ($g_{\perp}$) and g_z ($g_{\parallel}$) components, percent differences (PD) from the experimental values of the g_z^s ($g_{\parallel}^s$) and Δg values, and computed Löwdin atomic spin populations ρ_s on Cu for the set of 20 Cu(II) complexes, obtained with the **B3PW91 - 30%** functional.

Comp.	g_x	g_y	g_z	Δg	D(Δg)	‰PD($g_{x,y}$)	‰PD(g_z)	%PD(g_z^s)	%PD(Δg)	ρ_s(Cu)
**1**	2.057	2.057	2.206	0.149	-0.045	5.1	-15.4	-14.5	-23.2	0.710
**2**	2.026	2.031	2.096	0.068	0.008	1.6	5.3	13.3	13.0	0.458
**3**	2.058	2.061	2.215	0.156	-0.069	-0.4	-30.4	-24.6	-30.6	0.713
**4**	2.047	2.047	2.167	0.120	-0.039	1.8	-16.0	-17.7	-24.5	0.655
**5**	2.020	2.021	2.070	0.050	-0.020	-1.4	-11.1	-25.6	-29.3	0.465
**6**	2.039	2.062	2.181	0.130	-0.085	-0.7	-38.0	-32.5	-39.3	0.685
**7**	2.044	2.046	2.156	0.111	0.001	7.4	7.6	11.9	1.1	0.600
**8**	2.048	2.054	2.178	0.127	-0.063	0.4	-27.8	-26.2	-33.3	0.700
**9**	2.050	2.051	2.191	0.140	-0.075	1.8	-31.4	-27.3	-34.7	0.706
**10**	2.050	2.050	2.189	0.139	-0.071	-1.3	-32.8	-28.4	-34.0	0.674
**11**	2.051	2.058	2.166	0.112	-0.018	12.0	2.8	3.9	-14.1	0.615
**12**	2.053	2.055	2.186	0.132	-0.028	0.5	-12.1	-12.8	-17.4	0.674
**13**	2.039	2.039	2.151	0.112	-0.023	-29.1	-37.6	-36.2	-17.1	0.529
**14**	2.036	2.041	2.138				-16.1	-20.4		0.542
**15**	2.038	2.045	2.151				-22.9	-25.3		0.651
**16**	2.053	2.057	2.194	0.139	-0.062	-2.3	-29.4	-25.7	-30.7	0.693
**17**	2.048	2.050	2.183	0.134	-0.046	-5.2	-25.5	-24.0	-25.8	0.669
**18**	2.049	2.050	2.177	0.128	-0.012	-1.2	-6.7	-7.8	-8.8	0.692
**19**	2.065	2.073	2.221	0.152	-0.019	-9.2	-17.0	-15.0	-11.3	0.705
**20**	2.036	2.040	2.126	0.089	0.017	-3.5	4.4	8.2	22.9	0.520

Table S13. Calculated g-tensor components, differences (D) from the experimental values of the Δg parameter, per mille differences (‰PD) from the experimental values of the $g_{x,y}$ ($g_{\perp}$) and g_z ($g_{\parallel}$) components, percent differences (PD) from the experimental values of the g_z^s ($g_{\parallel}^s$) and Δg values, and computed Löwdin atomic spin populations ρ_s on Cu for the set of 20 Cu(II) complexes, obtained with the **B3PW91 - 35%** functional.

Comp.	g_x	g_y	g_z	Δg	D(Δg)	‰PD($g_{x,y}$)	‰PD(g_z)	%PD(g_z^s)	%PD(Δg)	ρ_s(Cu)
**1**	2.062	2.062	2.226	0.163	-0.031	7.5	-6.8	-6.4	-15.8	0.733
**2**	2.029	2.033	2.111	0.080	0.020	3.1	12.4	31.3	32.6	0.484
**3**	2.063	2.066	2.236	0.171	-0.054	2.1	-21.6	-17.5	-23.9	0.741
**4**	2.051	2.051	2.184	0.133	-0.026	3.8	-8.3	-9.1	-16.3	0.680
**5**	2.021	2.023	2.079	0.057	-0.013	-0.3	-6.5	-15.0	-18.5	0.489
**6**	2.042	2.068	2.198	0.143	-0.072	1.4	-30.3	-26.0	-33.3	0.711
**7**	2.049	2.051	2.175	0.125	0.015	9.8	16.3	25.3	13.6	0.630
**8**	2.052	2.058	2.194	0.139	-0.051	2.4	-20.7	-19.5	-27.0	0.725
**9**	2.054	2.055	2.208	0.153	-0.062	3.7	-24.0	-20.9	-28.8	0.728
**10**	2.054	2.055	2.207	0.152	-0.058	0.8	-24.7	-21.5	-27.4	0.700
**11**	2.057	2.065	2.187	0.126	-0.004	15.3	12.5	17.2	-3.0	0.647
**12**	2.058	2.060	2.205	0.146	-0.014	2.9	-3.6	-3.8	-8.7	0.700
**13**	2.044	2.045	2.172	0.128	-0.007	-26.4	-28.0	-26.9	-5.4	0.566
**14**	2.042	2.048	2.160				-5.9	-7.5		0.592
**15**	2.042	2.049	2.166				-16.4	-18.0		0.676
**16**	2.059	2.062	2.214	0.153	-0.048	0.2	-21.0	-18.3	-23.7	0.721
**17**	2.053	2.055	2.202	0.148	-0.032	-3.0	-17.1	-16.2	-17.9	0.698
**18**	2.053	2.054	2.194	0.140	0.000	0.9	0.7	0.9	-0.1	0.718
**19**	2.070	2.080	2.244	0.168	-0.003	-6.1	-6.7	-5.9	-1.5	0.738
**20**	2.042	2.045	2.145	0.102	0.030	-0.8	13.2	24.4	41.2	0.554

Table S14. Calculated g-tensor components, differences (D) from the experimental values of the Δg parameter, per mille differences (‰PD) from the experimental values of the $g_{x,y}$ ($g_\perp$) and g_z ($g_\parallel$) components, percent differences (PD) from the experimental values of the g_z^s ($g_\parallel^s$) and Δg values, and computed Löwdin atomic spin populations ρ_s on Cu for the set of 20 Cu(II) complexes, obtained with the **B3PW91 - 40%** functional.

Comp.	g_x	g_y	g_z	Δg	D(Δg)	‰PD($g_{x,y}$)	‰PD(g_z)	%PD(g_z^s)	%PD(Δg)	ρ_s(Cu)
**1**	2.067	2.067	2.246	0.179	-0.015	9.9	2.3	2.1	-7.9	0.755
**2**	2.034	2.037	2.129	0.094	0.034	5.1	21.1	53.1	55.9	0.515
**3**	2.068	2.070	2.256	0.187	-0.038	4.4	-12.6	-10.2	-16.9	0.765
**4**	2.055	2.055	2.202	0.147	-0.012	5.9	0.1	0.1	-7.4	0.705
**5**	2.024	2.026	2.091	0.066	-0.004	1.0	-1.2	-2.7	-6.4	0.515
**6**	2.045	2.074	2.216	0.157	-0.058	3.6	-22.4	-19.2	-27.0	0.735
**7**	2.054	2.056	2.195	0.140	0.030	12.4	25.7	40.0	27.2	0.660
**8**	2.057	2.061	2.210	0.151	-0.039	4.4	-13.3	-12.5	-20.4	0.747
**9**	2.058	2.059	2.225	0.167	-0.048	5.6	-16.4	-14.3	-22.5	0.749
**10**	2.059	2.059	2.226	0.167	-0.043	2.9	-16.3	-14.1	-20.3	0.725
**11**	2.064	2.072	2.210	0.142	0.012	18.8	23.3	32.0	9.5	0.679
**12**	2.063	2.065	2.225	0.161	0.001	5.3	5.4	5.7	0.7	0.726
**13**	2.051	2.052	2.198	0.147	0.012	-23.0	-16.4	-15.8	8.6	0.606
**14**	2.048	2.056	2.185				5.4	6.9		0.640
**15**	2.045	2.054	2.181				-9.4	-10.4		0.700
**16**	2.064	2.067	2.233	0.168	-0.033	2.7	-12.2	-10.7	-16.4	0.747
**17**	2.058	2.059	2.221	0.163	-0.017	-0.7	-8.4	-7.9	-9.6	0.724
**18**	2.058	2.058	2.211	0.153	0.013	2.9	8.5	9.8	9.0	0.741
**19**	2.076	2.088	2.267	0.186	0.015	-3.0	3.7	3.3	8.5	0.766
**20**	2.049	2.051	2.167	0.117	0.045	2.4	23.7	43.7	62.7	0.591

Table S15. Calculated g-tensor components, differences (D) from the experimental values of the Δg parameter, per mille differences (‰PD) from the experimental values of the $g_{x,y}$ ($g_{\perp}$) and g_z ($g_{\parallel}$) components, percent differences (PD) from the experimental values of the g_z^s ($g_{\parallel}^s$) and Δg values, and computed Löwdin atomic spin populations ρ_s on Cu for the set of 20 Cu(II) complexes, obtained with the **B3PW91 - 45%** functional.

Comp.	g_x	g_y	g_z	Δg	D(Δg)	‰PD($g_{x,y}$)	‰PD(g_z)	%PD(g_z^s)	%PD(Δg)	ρ_s(Cu)
**1**	2.072	2.072	2.267	0.195	0.001	12.4	11.7	11.0	0.5	0.775
**2**	2.040	2.041	2.151	0.110	0.050	7.6	31.4	79.2	83.6	0.550
**3**	2.073	2.075	2.277	0.203	-0.022	6.7	-3.4	-2.8	-9.6	0.787
**4**	2.059	2.059	2.222	0.162	0.003	8.0	8.9	9.8	2.0	0.728
**5**	2.027	2.030	2.103	0.075	0.005	2.6	5.0	11.5	7.4	0.543
**6**	2.048	2.079	2.235	0.171	-0.044	5.7	-14.2	-12.2	-20.4	0.757
**7**	2.059	2.062	2.217	0.156	0.046	15.1	35.9	55.8	41.9	0.689
**8**	2.061	2.065	2.227	0.164	-0.026	6.4	-5.8	-5.4	-13.6	0.768
**9**	2.062	2.062	2.243	0.181	-0.034	7.4	-8.4	-7.4	-16.0	0.768
**10**	2.063	2.063	2.246	0.183	-0.027	4.9	-7.5	-6.5	-12.9	0.748
**11**	2.071	2.080	2.236	0.160	0.030	22.5	35.1	48.0	23.1	0.711
**12**	2.068	2.070	2.246	0.177	0.017	7.7	14.8	15.5	10.6	0.750
**13**	2.059	2.061	2.229	0.168	0.033	-19.0	-2.9	-2.8	24.8	0.648
**14**	2.054	2.065	2.211				17.3	22.1		0.684
**15**	2.049	2.058	2.197				-2.1	-2.3		0.723
**16**	2.069	2.072	2.254	0.183	-0.018	5.1	-3.2	-2.8	-8.8	0.770
**17**	2.063	2.063	2.241	0.178	-0.002	1.5	0.6	0.6	-1.0	0.749
**18**	2.062	2.062	2.228	0.166	0.026	4.9	16.3	18.9	18.4	0.762
**19**	2.081	2.094	2.291	0.203	0.032	-0.1	14.2	12.5	18.8	0.790
**20**	2.056	2.058	2.192	0.135	0.063	6.1	35.7	65.8	87.6	0.630

Table S16. Calculated g-tensor components, differences (D) from the experimental values of the Δg parameter, per mille differences (‰PD) from the experimental values of the $g_{x,y}$ ($g_{\perp}$) and g_z ($g_{\parallel}$) components, percent differences (PD) from the experimental values of the g_z^s ($g_{\parallel}^s$) and Δg values, and computed Löwdin atomic spin populations ρ_s on Cu for the set of 20 Cu(II) complexes, obtained with the **B3PW91 - 50%** functional.

Comp.	g_x	g_y	g_z	Δg	D(Δg)	‰PD($g_{x,y}$)	‰PD(g_z)	%PD(g_z^s)	%PD(Δg)	ρ_s(Cu)
**1**	2.077	2.077	2.289	0.212	0.018	14.8	21.5	20.2	9.3	0.793
**2**	2.046	2.047	2.176	0.129	0.069	10.6	43.6	109.8	115.7	0.588
**3**	2.077	2.079	2.298	0.220	-0.005	8.8	5.8	4.7	-2.2	0.806
**4**	2.064	2.064	2.242	0.178	0.019	10.1	18.1	19.9	12.0	0.750
**5**	2.031	2.033	2.118	0.086	0.016	4.4	11.9	27.5	22.8	0.573
**6**	2.051	2.085	2.254	0.186	-0.029	7.7	-5.9	-5.1	-13.6	0.777
**7**	2.065	2.068	2.240	0.173	0.063	17.9	46.5	72.3	57.4	0.717
**8**	2.065	2.069	2.244	0.177	-0.013	8.2	1.9	1.8	-6.6	0.786
**9**	2.066	2.066	2.261	0.195	-0.020	9.2	-0.3	-0.3	-9.1	0.785
**10**	2.067	2.067	2.267	0.199	-0.011	7.0	1.6	1.4	-5.1	0.769
**11**	2.078	2.088	2.262	0.179	0.049	26.2	47.4	65.0	37.9	0.740
**12**	2.073	2.074	2.267	0.194	0.034	10.1	24.5	25.7	21.0	0.771
**13**	2.068	2.071	2.263	0.193	0.058	-14.5	12.3	11.8	42.9	0.689
**14**	2.061	2.073	2.237				29.5	37.5		0.722
**15**	2.052	2.063	2.214				5.5	6.0		0.744
**16**	2.074	2.077	2.274	0.199	-0.002	7.4	5.9	5.1	-1.0	0.790
**17**	2.067	2.068	2.262	0.194	0.014	3.7	9.8	9.2	8.0	0.771
**18**	2.066	2.066	2.245	0.179	0.039	6.8	24.3	28.1	28.1	0.781
**19**	2.087	2.101	2.315	0.221	0.050	2.7	24.7	21.7	29.3	0.810
**20**	2.065	2.066	2.221	0.155	0.083	10.1	49.0	90.4	115.3	0.668

Table S17. Calculated g-tensor components, differences (D) from the experimental values of the Δg parameter, per mille differences (‰PD) from the experimental values of the $g_{x,y}$ ($g_{\perp}$) and g_z ($g_{\parallel}$) components, percent differences (PD) from the experimental values of the g_z^s ($g_{\parallel}^s$) and Δg values, and computed Löwdin atomic spin populations ρ_s on Cu for the set of 20 Cu(II) complexes, obtained with the **B3PW91 - 55%** functional.

Comp.	g_x	g_y	g_z	Δg	D(Δg)	‰PD($g_{x,y}$)	‰PD(g_z)	%PD(g_z^s)	%PD(Δg)	ρ_s(Cu)
**1**	2.082	2.082	2.312	0.230	0.036	17.1	31.6	29.7	18.6	0.810
**2**	2.053	2.054	2.204	0.151	0.091	14.0	57.3	144.4	151.7	0.627
**3**	2.081	2.083	2.320	0.237	0.012	10.7	15.1	12.2	5.5	0.823
**4**	2.068	2.068	2.263	0.195	0.036	12.1	27.5	30.3	22.5	0.770
**5**	2.035	2.038	2.134	0.098	0.028	6.6	19.7	45.4	39.9	0.603
**6**	2.053	2.090	2.273	0.201	-0.014	9.6	2.5	2.2	-6.5	0.794
**7**	2.070	2.074	2.263	0.191	0.081	20.7	57.5	89.3	73.6	0.742
**8**	2.069	2.072	2.261	0.191	0.001	9.9	9.6	9.0	0.6	0.802
**9**	2.069	2.070	2.280	0.211	-0.004	11.0	8.1	7.0	-2.0	0.801
**10**	2.071	2.072	2.288	0.216	0.006	8.9	11.0	9.5	3.1	0.788
**11**	2.085	2.096	2.290	0.199	0.069	29.9	60.2	82.5	53.4	0.766
**12**	2.078	2.079	2.289	0.211	0.051	12.3	34.5	36.2	31.8	0.790
**13**	2.078	2.081	2.299	0.219	0.084	-9.8	28.6	27.5	62.6	0.728
**14**	2.067	2.081	2.263				41.6	53.0		0.753
**15**	2.055	2.067	2.231				13.2	14.5		0.763
**16**	2.078	2.081	2.295	0.215	0.014	9.5	15.0	13.1	7.1	0.808
**17**	2.071	2.072	2.283	0.211	0.031	5.7	19.1	18.0	17.3	0.790
**18**	2.069	2.070	2.263	0.193	0.053	8.6	32.4	37.5	38.2	0.798
**19**	2.092	2.107	2.339	0.240	0.069	5.3	35.3	31.1	40.2	0.827
**20**	2.074	2.075	2.251	0.176	0.104	14.3	63.2	116.6	145.1	0.704

Table S18. Calculated g-tensor components, differences (D) from the experimental values of the Δg parameter, per mille differences (‰PD) from the experimental values of the $g_{x,y}$ ($g_\perp$) and g_z ($g_\parallel$) components, percent differences (PD) from the experimental values of the g_z^s ($g_\parallel^s$) and Δg values, and computed Löwdin atomic spin populations ρ_s on Cu for the set of 20 Cu(II) complexes, obtained with the **LC-BLYP** functional.

Comp.	g_x	g_y	g_z	Δg	D(Δg)	‰PD($g_{x,y}$)	‰PD(g_z)	%PD(g_z^s)	%PD(Δg)	ρ_s(Cu)
**1**	2.011	2.011	2.037	0.026	-0.168	-17.8	-91.2	-85.6	-86.5	0.642
**2**	2.006	2.008	2.015	0.008	-0.052	-8.7	-33.5	-84.4	-86.9	0.419
**3**	2.012	2.012	2.040	0.028	-0.197	-23.4	-107.3	-86.7	-87.6	0.644
**4**	2.010	2.010	2.032	0.022	-0.137	-16.2	-77.2	-85.1	-86.1	0.591
**5**	2.006	2.006	2.014	0.009	-0.061	-8.6	-37.5	-86.6	-87.5	0.432
**6**	2.009	2.012	2.036	0.025	-0.190	-20.2	-102.0	-87.3	-88.3	0.623
**7**	2.009	2.010	2.031	0.021	-0.089	-10.1	-51.1	-79.4	-80.9	0.539
**8**	2.010	2.012	2.035	0.024	-0.166	-19.1	-91.5	-86.2	-87.3	0.636
**9**	2.011	2.011	2.036	0.025	-0.190	-17.8	-100.0	-87.1	-88.2	0.642
**10**	2.011	2.011	2.036	0.025	-0.185	-20.6	-100.5	-87.2	-88.1	0.609
**11**	2.009	2.010	2.030	0.020	-0.110	-10.1	-60.4	-82.7	-84.5	0.548
**12**	2.010	2.011	2.034	0.024	-0.136	-20.7	-80.9	-84.9	-85.3	0.604
**13**	2.008	2.009	2.030	0.022	-0.113	-43.5	-91.7	-88.0	-84.0	0.464
**14**	2.008	2.009	2.029				-66.4	-84.5		0.499
**15**	2.009	2.010	2.032				-77.0	-85.0		0.592
**16**	2.010	2.011	2.036	0.025	-0.176	-23.9	-99.5	-87.0	-87.5	0.624
**17**	2.010	2.011	2.036	0.025	-0.155	-24.1	-91.2	-85.9	-85.9	0.605
**18**	2.010	2.011	2.035	0.024	-0.116	-20.2	-71.8	-83.0	-82.9	0.627
**19**	2.011	2.012	2.039	0.027	-0.144	-36.5	-97.5	-85.8	-84.2	0.635
**20**	2.008	2.009	2.025	0.017	-0.055	-17.9	-43.5	-80.3	-77.1	0.464

Table S19. Calculated g-tensor components, differences (D) from the experimental values of the Δg parameter, per mille differences (‰PD) from the experimental values of the $g_{x,y}$ ($g_{\perp}$) and g_z ($g_{\parallel}$) components, percent differences (PD) from the experimental values of the g_z^s ($g_{\parallel}^s$) and Δg values, and computed Löwdin atomic spin populations ρ_s on Cu for the set of 20 Cu(II) complexes, obtained with the **CAM-B3LYP** functional.

Comp.	g_x	g_y	g_z	Δg	D(Δg)	‰PD($g_{x,y}$)	‰PD(g_z)	%PD(g_z^s)	%PD(Δg)	ρ_s(Cu)
**1**	2.020	2.020	2.073	0.053	-0.141	-13.3	-75.2	-70.6	-72.8	0.691
**2**	2.011	2.015	2.035	0.022	-0.038	-6.0	-24.1	-60.8	-63.4	0.448
**3**	2.021	2.022	2.077	0.056	-0.169	-18.6	-90.9	-73.5	-75.3	0.698
**4**	2.018	2.018	2.063	0.045	-0.114	-12.2	-63.2	-69.7	-71.8	0.637
**5**	2.010	2.010	2.030	0.021	-0.049	-6.5	-29.9	-68.9	-70.7	0.459
**6**	2.016	2.023	2.069	0.049	-0.166	-16.0	-87.5	-74.9	-77.0	0.671
**7**	2.017	2.018	2.060	0.043	-0.067	-6.2	-37.2	-57.9	-61.0	0.584
**8**	2.019	2.021	2.068	0.048	-0.142	-14.8	-76.8	-72.3	-74.5	0.685
**9**	2.019	2.019	2.070	0.051	-0.164	-13.5	-84.8	-73.8	-76.3	0.689
**10**	2.019	2.020	2.070	0.050	-0.160	-16.4	-85.4	-74.2	-76.1	0.658
**11**	2.017	2.019	2.060	0.042	-0.088	-5.9	-46.3	-63.5	-67.8	0.596
**12**	2.019	2.020	2.067	0.048	-0.112	-16.3	-65.8	-69.1	-70.1	0.655
**13**	2.015	2.015	2.057	0.042	-0.093	-40.4	-79.5	-76.4	-68.8	0.510
**14**	2.015	2.015	2.055				-54.1	-68.8		0.542
**15**	2.016	2.018	2.061				-63.9	-70.5		0.636
**16**	2.019	2.021	2.071	0.051	-0.150	-19.4	-84.2	-73.6	-74.8	0.677
**17**	2.019	2.019	2.069	0.050	-0.130	-19.9	-76.4	-72.0	-72.2	0.654
**18**	2.019	2.020	2.067	0.048	-0.092	-16.0	-56.9	-65.8	-65.7	0.677
**19**	2.021	2.023	2.076	0.054	-0.117	-31.5	-80.8	-71.1	-68.3	0.691
**20**	2.015	2.016	2.050	0.034	-0.038	-14.5	-31.8	-58.7	-52.2	0.505

Table S20. Calculated *g*-tensor components, differences (D) from the experimental values of the Δ*g* parameter, per mille differences (‰PD) from the experimental values of the $g_{x,y}$ ($g_\perp$) and g_z ($g_\parallel$) components, percent differences (PD) from the experimental values of the g_z^s ($g_\parallel^s$) and Δ*g* values, and computed Löwdin atomic spin populations ρ_s on Cu for the set of 20 Cu(II) complexes, obtained with the **B2PLYP** functional.

Comp.	g_x	g_y	g_z	Δ*g*	D(Δ*g*)	‰PD($g_{x,y}$)	‰PD(g_z)	%PD(g_z^s)	%PD(Δ*g*)	ρ_s(Cu)
**1**	2.049	2.049	2.199	0.150	-0.044	0.8	-18.9	-17.7	-22.7	0.693
**2**	1.987	1.989	1.996	0.008	-0.052	-18.3	-42.7	-107.5	-86.5	0.286
**3**	2.052	2.056	2.218	0.164	-0.061	-2.9	-29.1	-23.5	-27.0	0.709
**4**	2.036	2.036	2.144	0.108	-0.051	-3.3	-26.4	-29.1	-32.3	0.621
**5**	1.994	1.995	1.997	0.002	-0.068	-14.0	-45.8	-105.7	-96.6	0.317
**6**	2.035	2.051	2.171	0.128	-0.087	-4.3	-42.2	-36.1	-40.3	0.668
**7**	2.026	2.029	2.116	0.089	-0.021	-1.2	-11.0	-17.2	-19.2	0.540
**8**	2.041	2.048	2.169	0.125	-0.065	-2.9	-31.7	-29.9	-34.3	0.683
**9**	2.043	2.043	2.180	0.137	-0.078	-2.0	-36.3	-31.6	-36.3	0.685
**10**	2.041	2.041	2.172	0.131	-0.079	-5.7	-40.2	-34.9	-37.7	0.648
**11**	2.031	2.038	2.125	0.090	-0.040	2.3	-16.1	-22.1	-30.4	0.562
**12**	2.043	2.044	2.167	0.124	-0.036	-4.8	-20.9	-22.0	-22.7	0.644
**13**	1.995	2.002	2.062	0.063	-0.072	-48.4	-77.5	-74.4	-53.0	0.416
**14**	2.018	2.023	2.099				-34.1	-43.4		0.508
**15**	2.030	2.033	2.129				-33.3	-36.7		0.615
**16**	2.045	2.049	2.186	0.139	-0.062	-6.2	-33.1	-28.9	-30.8	0.677
**17**	2.040	2.042	2.170	0.129	-0.051	-9.3	-31.2	-29.4	-28.2	0.649
**18**	2.041	2.043	2.167	0.125	-0.015	-4.8	-11.4	-13.2	-10.9	0.673
**19**	2.057	2.064	2.221	0.160	-0.011	-13.2	-17.0	-15.0	-6.4	0.703
**20**	2.001	2.006	2.041	0.038	-0.034	-20.1	-35.7	-65.9	-47.9	0.405

Table S21. Calculated g-tensor components, differences (D) from the experimental values of the Δg parameter, per mille differences (‰PD) from the experimental values of the $g_{x,y}$ ($g_{\perp}$) and g_z ($g_{\parallel}$) components, percent differences (PD) from the experimental values of the g_z^s ($g_{\parallel}^s$) and Δg values, and computed Löwdin atomic spin populations ρ_s on Cu for the set of 20 Cu(II) complexes, obtained with the **DSD-PBEP86** functional.

Comp.	g_x	g_y	g_z	Δg	D(Δg)	‰PD($g_{x,y}$)	‰PD(g_z)	%PD(g_z^s)	%PD(Δg)	ρ_s(Cu)
**1**	2.064	2.064	2.273	0.209	0.015	8.2	14.3	13.4	7.9	0.759
**2**	2.007	2.007	2.067	0.059	-0.001	-8.7	-8.9	-22.3	-1.5	0.462
**3**	2.068	2.070	2.296	0.227	0.002	4.3	4.9	4.0	1.1	0.779
**4**	2.049	2.049	2.208	0.158	-0.001	3.1	2.5	2.8	-0.4	0.701
**5**	1.995	2.002	2.016	0.017	-0.053	-12.0	-36.8	-85.0	-75.5	0.414
**6**	2.044	2.068	2.235	0.179	-0.036	2.0	-14.3	-12.2	-17.0	0.738
**7**	2.045	2.049	2.196	0.149	0.039	8.3	26.0	40.3	35.2	0.654
**8**	2.053	2.059	2.226	0.170	-0.020	3.0	-6.3	-5.9	-10.6	0.749
**9**	2.054	2.055	2.240	0.186	-0.029	3.5	-9.6	-8.4	-13.5	0.746
**10**	2.055	2.055	2.243	0.188	-0.022	0.9	-8.8	-7.6	-10.3	0.730
**11**	2.061	2.066	2.237	0.173	0.043	16.6	35.7	48.9	33.3	0.670
**12**	2.056	2.057	2.246	0.189	0.029	1.8	14.9	15.7	18.4	0.697
**13**	2.034	2.036	2.194	0.159	0.024	-30.8	-18.2	-17.5	17.9	0.622
**14**	2.047	2.050	2.197				11.0	14.0		0.670
**15**	2.041	2.046	2.179				-10.3	-11.4		0.691
**16**	2.062	2.064	2.259	0.196	-0.005	1.5	-0.8	-0.7	-2.5	0.755
**17**	2.055	2.056	2.241	0.186	0.006	-2.2	0.6	0.5	3.2	0.730
**18**	2.055	2.055	2.226	0.171	0.031	1.4	15.6	18.1	22.5	0.742
**19**	2.075	2.088	2.314	0.232	0.061	-3.2	24.3	21.3	35.9	0.780
**20**	2.031	2.034	2.142	0.110	0.038	-6.1	11.9	21.9	52.2	0.584

Table S22. Calculated g-tensor components, differences (D) from the experimental values of the Δg parameter, per mille differences (‰PD) from the experimental values of the $g_{x,y}$ ($g_{\perp}$) and g_z ($g_{\parallel}$) components, percent differences (PD) from the experimental values of the g_z^s ($g_{\parallel}^s$) and Δg values, and computed Löwdin atomic spin populations ρ_s on Cu for the set of 20 Cu(II) complexes, obtained with the **PBE-QIDH** functional.

Comp.	g_x	g_y	g_z	Δg	D(Δg)	‰PD($g_{x,y}$)	‰PD(g_z)	%PD(g_z^s)	%PD(Δg)	ρ_s(Cu)
**1**	2.067	2.068	2.285	0.218	0.024	10.0	19.7	18.5	12.2	0.769
**2**	2.016	2.016	2.092	0.077	0.017	-4.6	3.6	9.0	27.8	0.489
**3**	2.071	2.072	2.305	0.234	0.009	5.6	8.9	7.2	3.9	0.786
**4**	2.053	2.053	2.222	0.169	0.010	5.0	9.2	10.1	6.3	0.715
**5**	2.003	2.008	2.038	0.033	-0.037	-8.7	-26.4	-60.8	-53.5	0.449
**6**	2.046	2.073	2.245	0.186	-0.029	3.6	-9.5	-8.1	-13.4	0.749
**7**	2.050	2.054	2.211	0.159	0.049	10.6	33.2	51.6	44.9	0.670
**8**	2.056	2.062	2.236	0.177	-0.013	4.4	-1.9	-1.8	-6.9	0.758
**9**	2.057	2.058	2.252	0.194	-0.021	5.1	-4.4	-3.9	-9.5	0.757
**10**	2.058	2.058	2.254	0.196	-0.014	2.5	-4.0	-3.4	-6.7	0.738
**11**	2.064	2.073	2.250	0.182	0.052	19.0	41.8	57.2	39.7	0.682
**12**	2.061	2.062	2.258	0.196	0.036	4.2	20.3	21.3	22.7	0.718
**13**	2.042	2.043	2.214	0.172	0.037	-27.3	-9.2	-8.8	27.3	0.637
**14**	2.050	2.055	2.211				17.5	22.3		0.683
**15**	2.044	2.051	2.192				-4.6	-5.1		0.705
**16**	2.065	2.068	2.269	0.203	0.002	3.1	3.8	3.3	1.0	0.764
**17**	2.058	2.059	2.253	0.194	0.014	-0.6	5.9	5.5	8.0	0.741
**18**	2.058	2.058	2.236	0.179	0.039	2.8	20.2	23.4	27.6	0.752
**19**	2.078	2.090	2.320	0.235	0.064	-1.8	26.8	23.6	37.6	0.788
**20**	2.038	2.041	2.163	0.124	0.052	-2.6	21.9	40.4	71.9	0.604

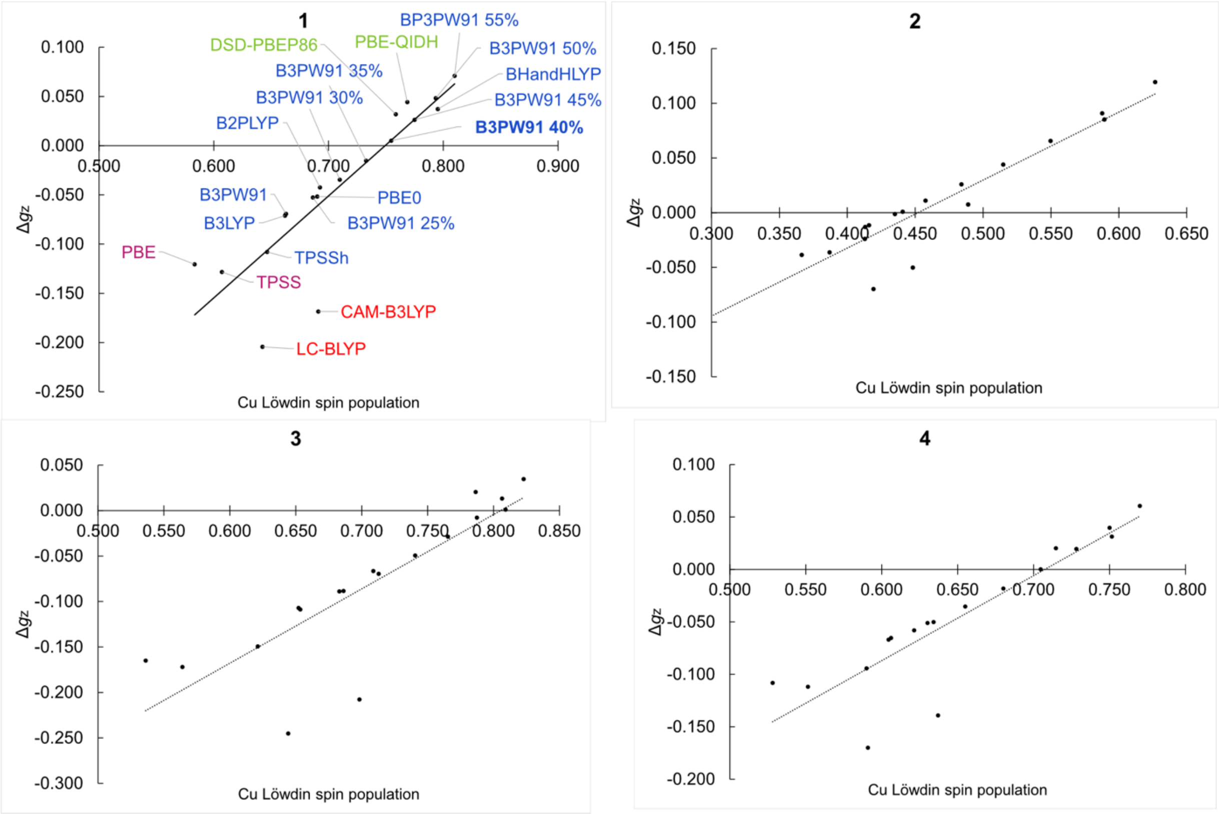

Figure S2. Correlation of the deviation, Δg_z, of the calculated g_z from the experimental value with the Cu spin populations computed with the respective functional for representative complexes **1**–**4**. Range-separated CAM-B3LYP and long-range corrected LC-BLYP consistently deviate from the observed trend.

Table S23. Calculated ^{14}N hyperfine coupling constants A_{iso} (MHz) for selected models.

Model	N1	N2	N3
**B2**	41	24	35
**C2-i**	34	0.5	38
**C3-i**	44	0.6	46

Cartesian coordinates of all optimized models

A1

0	N	-7.507404869	-11.6153558	-28.46639828
1	C	-7.412560215	-11.09611836	-29.85217025
2	C	-8.694215448	-10.321529	-30.25104239
3	O	-9.821068992	-10.73868169	-29.92722073
4	C	-7.181280087	-12.24807158	-30.85306069
5	C	-5.7917321	-12.77971668	-30.74795137
6	C	-4.84927717	-12.97200231	-31.7322232
7	N	-5.218097628	-13.11347076	-29.52953688
8	C	-3.961390195	-13.4980582	-29.77004489
9	N	-3.716173255	-13.42704824	-31.09280897
10	N	-8.495498256	-9.202086963	-30.9764593
11	C	-9.605812148	-8.41988703	-31.50150336
12	C	-9.149957446	-7.012513152	-31.92762083
13	O	-8.005318802	-6.59677985	-31.69389037
14	N	-10.09495917	-6.289819981	-32.57527565
15	C	-9.823183016	-5.014496993	-33.24485201
16	H	-10.72174499	-4.386782006	-33.24288399
17	H	-9.524393994	-5.204590002	-34.287202
18	H	-6.634855995	-8.496379015	-35.34582302
19	C	-6.877447011	-8.731616934	-34.30043392
20	C	-5.636856971	-8.999301064	-33.46504314
21	O	-5.737684513	-9.247275614	-32.24751607
22	H	-7.531247001	-9.610727015	-34.25221902
23	N	-4.438478474	-8.979167743	-34.09402248
24	C	-3.260095098	-9.541344976	-33.46429872
25	C	-2.196802917	-8.536882969	-33.03775925
26	O	-1.045905901	-8.933670979	-32.77854113
27	C	-2.636690181	-10.61287268	-34.3842672
28	O	-3.519243557	-11.71233943	-34.62237406
29	N	-2.568505643	-7.252756999	-32.89660556
30	C	-1.620982005	-6.229228081	-32.48717387
31	H	-1.491963008	-6.154969971	-31.39709605
32	H	-1.985815003	-5.271091977	-32.87373203
33	H	-12.461799	-11.734244	-31.272653
34	C	-12.32410202	-12.72164899	-31.732007
35	C	-11.95792497	-13.78797701	-30.724055
36	O	-12.49075851	-14.91197985	-30.7858791
37	H	-11.53192409	-12.62703682	-32.49077827
38	N	-11.06218552	-13.46672424	-29.75158227
39	C	-10.82191903	-14.38482303	-28.63875503
40	C	-10.97502598	-13.59496997	-27.34467797
41	O	-10.21536129	-12.64770459	-27.07201983
42	C	-9.480953109	-15.16476526	-28.70318445
43	C	-9.357707937	-15.91844531	-30.04218842
44	C	-9.39863407	-16.1240183	-27.50715868
45	C	-7.970026446	-16.50717606	-30.31404148
46	N	-11.98943957	-13.96469084	-26.53501109
47	C	-12.23761098	-13.28075801	-25.28306903
48	C	-11.18841081	-13.61474482	-24.21700357
49	O	-10.81712796	-14.77635796	-24.00617374
50	H	-13.21477001	-13.615247	-24.91178899
51	N	-10.74540267	-12.54064153	-23.52270161
52	C	-9.832752681	-12.63221671	-22.40634406
53	C	-10.36020124	-11.73023031	-21.2836977
54	O	-10.97624735	-10.68099365	-21.54491236
55	C	-8.395270126	-12.14515636	-22.76540829
56	C	-7.794237412	-12.79088335	-23.96478747

57	C	-7.47757029	-12.27214536	-25.19971375
58	N	-7.41725474	-14.12024574	-24.0299136
59	C	-6.906881313	-14.37631647	-25.2515476
60	N	-6.927960232	-13.26399968	-25.99296238
61	N	-10.06483029	-12.11580824	-20.03048087
62	C	-10.36296299	-11.26064098	-18.89707602
63	C	-9.689314015	-9.902912006	-19.04661399
64	H	-9.989706998	-11.76859501	-17.99901299
65	C	-8.172140207	-4.097299022	-25.63330826
66	C	-8.844191991	-5.273296018	-24.99355
67	C	-9.33368801	-5.385292983	-23.719741
68	C	-9.297051554	-6.445046018	-25.70376717
69	C	-10.03921869	-7.236574143	-24.77439987
70	C	-9.180331784	-6.887549937	-27.03440636
71	N	-10.03915811	-6.566051947	-23.57381653
72	C	-10.64696469	-8.44563601	-25.13829751
73	C	-9.789493085	-8.087598439	-27.39963533
74	C	-10.51364497	-8.858218594	-26.46389805
75	C	-5.717887048	-7.891400975	-20.59288698
76	C	-5.029188235	-8.970472432	-21.43837197
77	C	-5.160050002	-8.798478343	-22.95946086
78	H	-5.459144849	-9.938685099	-21.13497064
79	C	-6.598706805	-8.646849624	-23.45519255
80	N	-3.894341816	-4.989098672	-26.37741447
81	C	-4.014021005	-4.861951059	-27.81278702
82	H	-4.117209998	-3.802589965	-28.10056199
83	C	-5.114250636	-5.746194475	-28.42686783
84	C	-4.721853057	-7.202589258	-28.34894883
85	C	-5.200801541	-8.038754945	-27.33072487
86	C	-3.774246099	-7.721931778	-29.24688783
87	C	-4.72979325	-9.349830386	-27.19808638
88	C	-3.302550399	-9.02980043	-29.12151755
89	C	-3.773863384	-9.847567175	-28.08884207
90	Cu	-6.125680198	-13.10985631	-27.79502016
91	H	-8.447729142	-12.0085727	-28.27887497
92	H	-6.553198625	-10.41322435	-29.88774042
93	H	-7.931857382	-13.03395887	-30.66760094
94	H	-7.341507392	-11.88269951	-31.87594227
95	H	-4.871912853	-12.80352481	-32.80295602
96	H	-3.233451992	-13.80420178	-29.02829841
97	H	-7.545775447	-8.980144289	-31.31208388
98	H	-10.07068004	-8.924333433	-32.36576967
99	H	-10.38601409	-8.335434231	-30.73010855
100	H	-10.97962224	-6.745083184	-32.78778396
101	H	-9.01094366	-4.498151258	-32.72275154
102	H	-7.417028597	-7.88233182	-33.86254814
103	H	-4.413321213	-8.782284127	-35.09298812
104	H	-3.610054078	-10.03077585	-32.54110663
105	H	-2.32364307	-10.14679905	-35.33592056
106	H	-1.747222932	-11.02022236	-33.89255501
107	H	-3.518848987	-6.985182454	-33.13753511
108	H	-0.640148491	-6.444182248	-32.92969349
109	H	-13.24801209	-13.02547676	-32.23495487
110	H	-10.6356452	-12.53164616	-29.73098819
111	H	-11.62568966	-15.13408862	-28.70855982
112	H	-8.656174029	-14.4306386	-28.6383093
113	H	-10.11102167	-16.72372214	-30.06313985
114	H	-9.614862635	-15.2340381	-30.86427029
115	H	-10.2395821	-16.83566126	-27.53016267
116	H	-9.433433434	-15.59163656	-26.54650276
117	H	-8.468206256	-16.70653591	-27.52488869
118	H	-7.667894833	-17.23408163	-29.54457327

119	H	-7.209449624	-15.71053847	-30.36945208
120	H	-7.950260583	-17.03444379	-31.27877758
121	H	-12.57250687	-14.75650355	-26.78801976
122	H	-12.28870664	-12.19688251	-25.45701555
123	H	-11.09870022	-11.61127129	-23.74509059
124	H	-9.803201788	-13.68306859	-22.08622476
125	H	-8.440813922	-11.0645404	-22.95685669
126	H	-7.754019048	-12.29535126	-21.8828618
127	H	-7.614799788	-11.25521637	-25.54722091
128	H	-6.535056685	-15.34203903	-25.56952562
129	H	-9.541593322	-12.97303901	-19.87583177
130	H	-11.45226419	-11.14703491	-18.78638836
131	H	-9.241615536	-4.710284351	-22.87415484
132	H	-8.630249241	-6.300454494	-27.77277595
133	H	-10.4425167	-6.94380803	-22.70214654
134	H	-11.21607464	-9.033899923	-24.41652488
135	H	-9.713672587	-8.439827521	-28.42781939
136	H	-10.97860458	-9.791532897	-26.78488663
137	H	-3.959641118	-9.008978141	-21.17236384
138	H	-4.570447265	-7.926835456	-23.28695328
139	H	-4.691183495	-9.671465572	-23.44141281
140	H	-7.06324513	-7.719872933	-23.08920161
141	H	-7.223818212	-9.4849651	-23.11717054
142	H	-6.637954327	-8.623516712	-24.5530966
143	H	-3.04407021	-5.173524316	-28.22452904
144	H	-6.074504607	-5.573251597	-27.91773898
145	H	-5.241172661	-5.442339993	-29.47739132
146	H	-5.941043743	-7.652714675	-26.62663384
147	H	-3.393933098	-7.085193936	-30.04879684
148	H	-5.107545476	-9.97820677	-26.38846
149	H	-2.560489796	-9.411216583	-29.825253
150	H	-3.39752666	-10.86561278	-27.98292962
151	H	-2.830178235	-13.65254058	-31.5391793
152	H	-7.517321193	-14.80887041	-23.287349
153	H	-7.38028356	-10.83656538	-27.81118477
154	H	-4.248249851	-11.40159906	-35.18970995
155	N	-10.46444469	-8.838213452	-18.75312139
156	C	-10.11820497	-7.498699013	-19.19436703
157	H	-10.02216301	-6.80933199	-18.33938598
158	C	-11.21115737	-6.975546467	-20.12126361
159	H	-10.98344916	-5.941327727	-20.41506511
160	O	-11.32326533	-7.733784958	-21.33652869
161	O	-8.517410677	-9.814209573	-19.43884698
162	H	-11.4286586	-9.00726741	-18.47248206
163	H	-12.18040698	-6.971649712	-19.58991935
164	H	-9.148718227	-7.548425987	-19.70414194
165	H	-11.25592283	-8.69674782	-21.1439768
166	H	-7.000727004	-2.350806	-25.16716801
167	C	-7.552380983	-3.132924991	-24.62274098
168	C	-6.547010026	-3.871511005	-23.75651203
169	O	-5.732693538	-4.682974266	-24.23313299
170	N	-6.569866415	-3.584176099	-22.43712921
171	C	-5.630933997	-4.177287974	-21.50524193
172	C	-6.145717995	-5.486160023	-20.90963803
173	O	-7.269374261	-5.539301402	-20.37932044
174	H	-5.472591997	-3.445791006	-20.70196803
175	N	-5.326678276	-6.549752127	-21.01799593
176	H	-8.891191142	-3.544588711	-26.2622353
177	H	-7.38538643	-4.449111993	-26.31817408
178	H	-8.31575766	-2.637334012	-24.00605614
179	H	-4.675628566	-4.325143308	-22.02478009
180	H	-4.420355382	-6.424826924	-21.46213653

181	H	-1.78951701	-5.874399996	-23.964633
182	C	-2.66444999	-5.300129018	-24.28938601
183	C	-2.712921998	-5.311855965	-25.80940598
184	O	-1.685118561	-5.569994292	-26.46913467
185	H	-2.536117182	-4.262051439	-23.94497064
186	H	-3.583099792	-5.686331472	-23.83371274
187	H	-4.692307744	-4.781375114	-25.76081254
188	H	-7.27945286	-2.952697258	-22.07765151
189	H	-5.431883972	-8.037666012	-19.53807801
190	H	-6.810794907	-7.955405973	-20.65570258
191	O	-5.734602974	-15.40741389	-27.72599313
192	O	-4.021035882	-13.0377439	-26.72388383
193	H	-4.802768997	-15.6363569	-27.90635761
194	H	-3.895353435	-13.84613992	-26.19118769
195	H	-6.222764504	-15.77343538	-28.48946892
196	H	-4.073870153	-12.32731557	-26.05681566

Applied constraints

Specific atoms were constrained during optimization in order to maintain the structural effect of the protein matrix on the active site. The following command block was used in the Orca geometry optimization input files in order to keep atoms with the given numbers { C *number* C } constrained (atom numbers are indicated in the **A1** coordinates above). Note that counting starts from zero.

```
%geom
        Constraints
        { C 15 C }{ C 16 C }{ C 17 C }
        { C 18 C }{ C 19 C }{ C 20 C }{ C 22 C }
        { C 24 C }{ C 30 C }{ C 31 C }{ C 32 C }{ C 25 C }
        { C 33 C }{ C 34 C }{ C 35 C }{ C 39 C }
        { C 40 C }{ C 47 C }{ C 50 C }
        { C 62 C }{ C 63 C }{ C 64 C }{ C 156 C }{ C 157 C }
        { C 75 C }{ C 189 C }{ C 171 C }{ C 172 C }{ C 174 C }
        { C 166 C }{ C 167 C }{ C 168 C }{ C 66 C }{ C 67 C }
        { C 81 C }{ C 82 C }{ C 181 C }{ C 182 C }{ C 183 C }
        end
end
```

B1

N	-7.581376009	-11.584000371	-28.498019743
C	-7.535780630	-11.140386325	-29.910258782
C	-8.796426113	-10.327113502	-30.273478266
O	-9.929428265	-10.713942640	-29.931889285
C	-7.440152299	-12.350525428	-30.860212059
C	-6.093518693	-12.989021770	-30.821732295
C	-5.294231675	-13.385395174	-31.867892582
N	-5.419696969	-13.251339937	-29.635979590
C	-4.239765632	-13.791823286	-29.962338511
N	-4.143963399	-13.890162401	-31.301527812
N	-8.579522406	-9.211664334	-30.994075663
C	-9.680354504	-8.399688153	-31.483779514
C	-9.188185598	-7.015804427	-31.927599692
O	-8.034229008	-6.623595029	-31.696649824

N	-10.116102788	-6.283649258	-32.582209465
C	-9.823182991	-5.014496995	-33.244851985
H	-10.721745006	-4.386782001	-33.242884006
H	-9.524394001	-5.204590000	-34.287202007
H	-6.634855947	-8.496378906	-35.345823001
C	-6.877447133	-8.731617296	-34.300434019
C	-5.636856921	-8.999300818	-33.465042971
O	-5.741946916	-9.257695802	-32.249832850
H	-7.531246979	-9.610726931	-34.252218991
N	-4.438081005	-8.977361317	-34.092823581
C	-3.260095064	-9.541345110	-33.464299089
C	-2.196802957	-8.536882954	-33.037758914
O	-1.045543390	-8.932750254	-32.779278572
C	-2.635415503	-10.610208101	-34.385524580
O	-3.508412761	-11.718528375	-34.611979436
N	-2.569016649	-7.252863615	-32.895397324
C	-1.620982043	-6.229227945	-32.487174073
H	-1.491962987	-6.154970016	-31.397095957
H	-1.985814979	-5.271092037	-32.873731985
H	-12.461798990	-11.734244007	-31.272653011
C	-12.324102031	-12.721648987	-31.732006975
C	-11.957924968	-13.787977008	-30.724055023
O	-12.485747330	-14.914188218	-30.790078074
H	-11.530999718	-12.627057860	-32.489737560
N	-11.062485124	-13.466859056	-29.751320048
C	-10.821919023	-14.384822993	-28.638754978
C	-10.975025988	-13.594970002	-27.344678011
O	-10.211733251	-12.650297430	-27.070395475
C	-9.474110321	-15.158217410	-28.684529420
C	-9.304968699	-15.894004283	-30.026999931
C	-9.407964894	-16.129560251	-27.497529965
C	-7.933572655	-16.552096248	-30.212526377
N	-11.991097046	-13.961593887	-26.536716184
C	-12.237610980	-13.280757994	-25.283069009
C	-11.189534240	-13.622029613	-24.217600482
O	-10.828280483	-14.786942840	-24.006866135
H	-13.214770015	-13.615247009	-24.911788996
N	-10.733573323	-12.552396080	-23.524274672
C	-9.830033946	-12.658890080	-22.401257112
C	-10.350510685	-11.742297806	-21.284555828
O	-10.957964247	-10.690476310	-21.555147792
C	-8.377144233	-12.206582839	-22.745686306
C	-7.804298880	-12.792416847	-23.990564581
C	-7.401444318	-12.186047228	-25.159503703
N	-7.538233876	-14.137448930	-24.175475375
C	-7.006922938	-14.318290037	-25.401676078
N	-6.906629733	-13.141821015	-26.028663642
N	-10.060738118	-12.120055704	-20.027690364
C	-10.362963020	-11.260640995	-18.897076012
C	-9.689313998	-9.902911997	-19.046613994
H	-9.989706990	-11.768595003	-17.999012991
C	-8.168967987	-4.099633517	-25.633131801
C	-8.844192026	-5.273295987	-24.993549971
C	-9.333687993	-5.385293005	-23.719741024
C	-9.293022081	-6.447857932	-25.702404923
C	-10.033671275	-7.239183690	-24.772743807
C	-9.171023327	-6.893601349	-27.030759632
N	-10.035810937	-6.566021440	-23.572798508
C	-10.637081250	-8.450473007	-25.133303535
C	-9.774494311	-8.098238828	-27.393335029
C	-10.498495145	-8.867472212	-26.457748496
C	-5.717887019	-7.891401015	-20.592886939

C	-5.038428812	-8.969321423	-21.445425416
C	-5.203631982	-8.799358505	-22.963555025
H	-5.457375338	-9.939028087	-21.131671955
C	-6.654499861	-8.648798755	-23.422512085
N	-3.892614137	-4.982721227	-26.376731604
C	-4.014020931	-4.861951062	-27.812787015
H	-4.117210041	-3.802589953	-28.100561996
C	-5.114888992	-5.751131781	-28.419623498
C	-4.757118294	-7.213939105	-28.286061156
C	-5.265765364	-7.997291613	-27.239900174
C	-3.827962981	-7.799190972	-29.163076820
C	-4.844918742	-9.319916152	-27.060240172
C	-3.407977776	-9.120646198	-28.992439743
C	-3.910455271	-9.885053765	-27.933281981
Cu	-6.131965160	-12.935044076	-27.836163089
H	-8.503991989	-11.994987196	-28.260258847
H	-6.644660287	-10.510535609	-30.031879050
H	-8.233726639	-13.066798232	-30.593312370
H	-7.640879084	-12.022321822	-31.888904128
H	-5.444907819	-13.347735441	-32.939665879
H	-3.459534888	-14.090894108	-29.273793808
H	-7.627351925	-8.986380312	-31.316380194
H	-10.191245145	-8.894542758	-32.326913354
H	-10.429790722	-8.282306401	-30.686130540
H	-11.011126235	-6.720505185	-32.789034510
H	-9.010510423	-4.504769034	-32.717307165
H	-7.417714747	-7.883184103	-33.861480437
H	-4.411363484	-8.774059723	-35.090451275
H	-3.609360075	-10.032522521	-32.541813310
H	-2.333743825	-10.143385775	-35.340934202
H	-1.738279388	-11.007616442	-33.899493543
H	-3.519709639	-6.985451284	-33.135026088
H	-0.640442120	-6.444607176	-32.930055146
H	-13.247534561	-13.025630418	-32.235762508
H	-10.655208394	-12.522552368	-29.721156697
H	-11.622249442	-15.137432571	-28.709335467
H	-8.653963629	-14.422573801	-28.588426120
H	-10.096748005	-16.656887450	-30.115751989
H	-9.472222331	-15.180486633	-30.848912220
H	-10.220959132	-16.870743752	-27.562132200
H	-9.503640459	-15.611798619	-26.532854016
H	-8.455264436	-16.675375249	-27.484628574
H	-7.761505320	-17.362254157	-29.489652961
H	-7.121627086	-15.817885655	-30.094518408
H	-7.846456981	-16.986615630	-31.219072014
H	-12.575331559	-14.752124639	-26.791201615
H	-12.286298645	-12.196179076	-25.453133759
H	-11.082219600	-11.619621877	-23.740157565
H	-9.826048605	-13.709114447	-22.077638116
H	-8.377409856	-11.115082403	-22.868607656
H	-7.736429801	-12.434458638	-21.879563425
H	-7.435798312	-11.132602417	-25.413693014
H	-6.711103681	-15.283620770	-25.796582938
H	-9.548487771	-12.982512827	-19.865182488
H	-11.452583766	-11.148181532	-18.788776738
H	-9.242908238	-4.709153783	-22.874959752
H	-8.620885223	-6.307172985	-27.769481696
H	-10.441806496	-6.942389074	-22.700930753
H	-11.205682226	-9.037968650	-24.410632458
H	-9.694485189	-8.453246422	-28.420039236
H	-10.959226950	-9.803593215	-26.776447240
H	-3.963638227	-9.001351430	-21.200526048

H	-4.622794550	-7.927392090	-23.305375218
H	-4.746350083	-9.672315884	-23.456471908
H	-7.108557639	-7.718376217	-23.052389320
H	-7.271480427	-9.483280953	-23.060971913
H	-6.724485095	-8.634879964	-24.519022537
H	-3.044232581	-5.175626317	-28.223611026
H	-6.082475204	-5.544341292	-27.937105448
H	-5.215214957	-5.480509112	-29.482035880
H	-5.993447772	-7.559370579	-26.553592923
H	-3.423811899	-7.206803795	-29.987372711
H	-5.243437583	-9.905285425	-26.229189526
H	-2.682676808	-9.553327894	-29.683914234
H	-3.575503457	-10.913558966	-27.789840084
H	-3.341268301	-14.257078289	-31.807858876
H	-7.734921401	-14.882892450	-23.511070043
H	-7.454033640	-10.771930598	-27.882948374
H	-4.262520485	-11.408009745	-35.145755692
N	-10.465553325	-8.838413808	-18.754840568
C	-10.118204991	-7.498699009	-19.194367011
H	-10.022163002	-6.809331993	-18.339385991
C	-11.209166598	-6.974869595	-20.123046258
H	-10.981127448	-5.940511416	-20.415958383
O	-11.318176025	-7.732721803	-21.339316394
O	-8.516651961	-9.813942353	-19.436205805
H	-11.430001260	-9.007568273	-18.475067585
H	-12.179792312	-6.971861496	-19.594371089
H	-9.148132728	-7.548757630	-19.703062647
H	-11.248694353	-8.695451461	-21.147082704
H	-7.000727024	-2.350805993	-25.167168000
C	-7.552380944	-3.132925003	-24.622741003
C	-6.547010035	-3.871511006	-23.756512011
O	-5.733639284	-4.683757734	-24.233161246
N	-6.569793339	-3.584031534	-22.437210905
C	-5.630934013	-4.177287976	-21.505241929
C	-6.145717986	-5.486160001	-20.909638043
O	-7.268731551	-5.538790179	-20.377897994
H	-5.472591993	-3.445791009	-20.701968025
N	-5.326876143	-6.549829741	-21.018611204
H	-8.884588710	-3.549865496	-26.268387907
H	-7.378414988	-4.456496492	-26.310977216
H	-8.316762970	-2.637806322	-24.007041542
H	-4.675581978	-4.325119689	-22.024715622
H	-4.421179622	-6.425366618	-21.464261015
H	-1.789517010	-5.874400014	-23.964632992
C	-2.664449974	-5.300128976	-24.289386025
C	-2.712922028	-5.311855995	-25.809405985
O	-1.686715448	-5.575641251	-26.469444568
H	-2.536897228	-4.262230894	-23.944242588
H	-3.583013901	-5.687344396	-23.834337611
H	-4.690535454	-4.776203327	-25.759844633
H	-7.278277594	-2.951175892	-22.077927887
H	-5.431883992	-8.037665994	-19.538078042
H	-6.811273355	-7.952812773	-20.651337364
O	-4.237109706	-13.646951034	-27.033889398
H	-4.124701073	-13.278865345	-26.135595839
H	-4.235597510	-14.616446866	-26.906760045

B2

N	-7.550010218	-11.636262010	-28.562666959
C	-7.522795504	-11.195595111	-29.979658081

C	-8.764969333	-10.346131510	-30.314582513
O	-9.902519964	-10.715646857	-29.962312549
C	-7.506647111	-12.403800352	-30.932733163
C	-6.245606347	-13.201989821	-30.895960814
C	-5.577210165	-13.784783411	-31.949434387
N	-5.585149673	-13.535889510	-29.720889921
C	-4.550127000	-14.310527524	-30.059987355
N	-4.521545673	-14.476952429	-31.397686912
N	-8.543747785	-9.221319418	-31.021439271
C	-9.647346935	-8.399869728	-31.489932267
C	-9.167097815	-7.012013026	-31.932336945
O	-8.015319870	-6.610007675	-31.708433325
N	-10.106066698	-6.283831602	-32.578510419
C	-9.823183000	-5.014496973	-33.244851932
H	-10.721744997	-4.386782013	-33.242884022
H	-9.524394003	-5.204590005	-34.287202035
H	-6.634856012	-8.496378990	-35.345822925
C	-6.877446964	-8.731617046	-34.300434226
C	-5.636857066	-8.999300952	-33.465042822
O	-5.740092152	-9.253208329	-32.249016468
H	-7.531247010	-9.610727002	-34.252218957
N	-4.438375543	-8.979286405	-34.093960605
C	-3.260094890	-9.541345028	-33.464299260
C	-2.196803078	-8.536883046	-33.037758764
O	-1.044903959	-8.932222646	-32.781269383
C	-2.640152191	-10.620056592	-34.376171394
O	-3.517427848	-11.728982450	-34.582535661
N	-2.569157302	-7.252956763	-32.894867434
C	-1.620982018	-6.229227853	-32.487174178
H	-1.491962990	-6.154970055	-31.397095924
H	-1.985814981	-5.271092064	-32.873731966
H	-12.461799015	-11.734244005	-31.272653004
C	-12.324101947	-12.721649003	-31.732007004
C	-11.957925089	-13.787977005	-30.724054990
O	-12.495781968	-14.909927393	-30.784901661
H	-11.531153117	-12.626003243	-32.489781945
N	-11.056704641	-13.470456083	-29.755646102
C	-10.821918882	-14.384822887	-28.638754991
C	-10.975026063	-13.594970131	-27.344678011
O	-10.220411137	-12.643478535	-27.075176283
C	-9.478500735	-15.165930032	-28.685756531
C	-9.380156598	-15.984955854	-29.986980785
C	-9.360956168	-16.064413837	-27.446131930
C	-8.037898513	-16.694912636	-30.190167764
N	-11.989800538	-13.965135249	-26.534739445
C	-12.237610988	-13.280757867	-25.283069014
C	-11.186815311	-13.619391649	-24.217406212
O	-10.824727055	-14.784147145	-24.006313364
H	-13.214770005	-13.615247064	-24.911788996
N	-10.732224399	-12.548556894	-23.525857169
C	-9.820175419	-12.645177083	-22.408379178
C	-10.348737989	-11.738656196	-21.286648665
O	-10.961137084	-10.688099412	-21.552274499
C	-8.378072575	-12.161365505	-22.760505454
C	-7.763963134	-12.759319501	-23.979465492
C	-7.531326364	-12.210965390	-25.221832518
N	-7.238792160	-14.038433003	-24.053254518
C	-6.716205054	-14.226153194	-25.286765698
N	-6.881224423	-13.128355460	-26.025547513
N	-10.059970416	-12.118993165	-20.029432033
C	-10.362963005	-11.260641003	-18.897075931
C	-9.689314019	-9.902911984	-19.046614063

H	-9.989707000	-11.768594998	-17.999013030
C	-8.169057525	-4.098737010	-25.633785492
C	-8.844191665	-5.273296159	-24.993549963
C	-9.333688233	-5.385292874	-23.719741037
C	-9.303283114	-6.440805086	-25.705928058
C	-10.047280803	-7.232112644	-24.777388140
C	-9.187858502	-6.882276960	-27.037693871
N	-10.046611424	-6.563580925	-23.576266812
C	-10.654752653	-8.441531493	-25.142781967
C	-9.794991086	-8.082112688	-27.403293612
C	-10.519603500	-8.853862766	-26.467586508
C	-5.717887019	-7.891400994	-20.592886997
C	-5.057635094	-8.973546951	-21.452838144
C	-5.262197443	-8.811845629	-22.967852076
H	-5.472264042	-9.939909065	-21.123499337
C	-6.719024071	-8.598643726	-23.383289425
N	-3.892007271	-4.982223308	-26.376756456
C	-4.014021146	-4.861951039	-27.812786987
H	-4.117209930	-3.802589969	-28.100562007
C	-5.124236513	-5.741209379	-28.415629389
C	-4.790842258	-7.208196197	-28.270039936
C	-5.338420378	-7.979698673	-27.234894393
C	-3.852650779	-7.811063530	-29.125115865
C	-4.949600070	-9.310513325	-27.047123489
C	-3.463287702	-9.140666364	-28.944454782
C	-4.007038224	-9.894992848	-27.898405827
Cu	-6.058658775	-12.941573682	-27.872924820
H	-8.478543947	-12.025382373	-28.322013964
H	-6.612815893	-10.598759964	-30.126603824
H	-8.369825461	-13.044301769	-30.686729375
H	-7.662941711	-12.050748840	-31.961691349
H	-5.766464555	-13.770371346	-33.016181434
H	-3.837595761	-14.761633673	-29.379491265
H	-7.592375107	-9.012659417	-31.359752289
H	-10.172844793	-8.887960109	-32.328279888
H	-10.385328146	-8.287723194	-30.680870074
H	-10.997133047	-6.729022778	-32.784534416
H	-9.010570666	-4.500691102	-32.720852805
H	-7.417493408	-7.882765909	-33.862101286
H	-4.412653543	-8.780487526	-35.092484673
H	-3.610157579	-10.027866219	-32.539332968
H	-2.343380530	-10.165604062	-35.339045736
H	-1.741258656	-11.012874549	-33.889653254
H	-3.520946763	-6.986151115	-33.130727271
H	-0.640695973	-6.445195693	-32.930334849
H	-13.247537535	-13.025921775	-32.235523448
H	-10.643192867	-12.528128291	-29.729617233
H	-11.624798525	-15.135069985	-28.707474054
H	-8.653595726	-14.431085053	-28.668634038
H	-10.196815913	-16.726653040	-29.994572601
H	-9.564936408	-15.317513124	-30.842843514
H	-10.179778195	-16.801755472	-27.426030863
H	-9.402131885	-15.487654240	-26.511788808
H	-8.411795147	-16.616284025	-27.448897450
H	-7.857702865	-17.467543420	-29.429176831
H	-7.200816675	-15.981554346	-30.148203673
H	-8.007318028	-17.188459518	-31.172574583
H	-12.567020191	-14.762545919	-26.783158842
H	-12.286176706	-12.196711384	-25.456466230
H	-11.077130656	-11.616783996	-23.751050926
H	-9.795378018	-13.696248468	-22.089448379
H	-8.418548228	-11.075009246	-22.917155942

H	-7.739076416	-12.338022679	-21.881501159
H	-7.789104878	-11.216050914	-25.566606094
H	-6.222879904	-15.138694750	-25.600086589
H	-9.539237999	-12.976795573	-19.869417782
H	-11.452614813	-11.148312134	-18.789302040
H	-9.237852337	-4.712871944	-22.872455498
H	-8.637345501	-6.294583566	-27.775396286
H	-10.439796231	-6.948091528	-22.703754310
H	-11.223036357	-9.030848175	-24.421076346
H	-9.716003644	-8.436490979	-28.430685489
H	-10.982136294	-9.788347293	-26.788914150
H	-3.977433888	-9.008105146	-21.233435524
H	-4.653583887	-7.970363999	-23.337304657
H	-4.860574888	-9.709638659	-23.464050765
H	-7.097585812	-7.618015944	-23.060893191
H	-7.375881900	-9.361860101	-22.942574263
H	-6.831768107	-8.650098910	-24.474924800
H	-3.047114207	-5.180655896	-28.226023270
H	-6.089514410	-5.517850895	-27.935778115
H	-5.219194158	-5.475842740	-29.479972464
H	-6.073783009	-7.527428951	-26.566179834
H	-3.418711059	-7.226847501	-29.940306354
H	-5.380282840	-9.890652914	-26.228279119
H	-2.730478004	-9.588038946	-29.618687203
H	-3.708867748	-10.933988030	-27.745564593
H	-3.830367315	-15.021883845	-31.907554942
H	-7.236446764	-14.729644955	-23.306604522
H	-7.432132928	-10.808837825	-27.966525481
H	-4.272420585	-11.423768486	-35.118119660
N	-10.465557771	-8.838301295	-18.754399456
C	-10.118204895	-7.498699049	-19.194366936
H	-10.022163052	-6.809331982	-18.339386026
C	-11.211286582	-6.975083089	-20.120972972
H	-10.981857597	-5.941931992	-20.417332931
O	-11.327520898	-7.735833546	-21.334075510
O	-8.515942443	-9.813405223	-19.433529848
H	-11.430766755	-9.007844892	-18.477520159
H	-12.179593459	-6.967879630	-19.587752042
H	-9.148738081	-7.548853940	-19.704125837
H	-11.253345445	-8.698038237	-21.140413436
H	-7.000726925	-2.350806048	-25.167167994
C	-7.552381259	-3.132924885	-24.622741018
C	-6.547009876	-3.871511052	-23.756512003
O	-5.729793820	-4.679768439	-24.233097335
N	-6.570584498	-3.584702019	-22.436822176
C	-5.630933988	-4.177288041	-21.505242053
C	-6.145718016	-5.486159994	-20.909637980
O	-7.266936005	-5.537771831	-20.374022153
H	-5.472592008	-3.445790977	-20.701967967
N	-5.326838732	-6.550193530	-21.020605412
H	-8.883716469	-3.546746482	-26.268227260
H	-7.377805103	-4.453455715	-26.312036042
H	-8.316862313	-2.638445037	-24.006512603
H	-4.676062940	-4.325082954	-22.025565182
H	-4.424266146	-6.426939345	-21.472949178
H	-1.789517004	-5.874399999	-23.964633001
C	-2.664450002	-5.300129012	-24.289385993
C	-2.712921960	-5.311855966	-25.809406004
O	-1.686306591	-5.575227807	-26.469261994
H	-2.538095985	-4.262299462	-23.943707161
H	-3.582553737	-5.689216313	-23.834818054
H	-4.689866457	-4.774827768	-25.759955607

H	-7.283205633	-2.957026507	-22.076787586
H	-5.431883995	-8.037666005	-19.538077998
H	-6.812121965	-7.946647630	-20.645621502
O	-4.377116128	-13.453622836	-27.098336939
H	-3.687752502	-13.335622853	-27.776331722

C1

N	-7.897516693	-11.115359033	-28.221656987
C	-7.579459962	-10.981504599	-29.686538376
C	-8.807946354	-10.304563706	-30.320421160
O	-9.951453303	-10.692796361	-30.016236617
C	-7.292208233	-12.353433758	-30.343776847
C	-5.901147164	-12.903478171	-30.216254517
C	-4.912717300	-12.822844710	-31.173583486
N	-5.398793455	-13.644305491	-29.142418027
C	-4.142955979	-13.981293690	-29.457315177
N	-3.826240507	-13.499316478	-30.674618577
N	-8.549031677	-9.282488812	-31.150040854
C	-9.612173118	-8.522000552	-31.780395051
C	-9.179091701	-7.067951045	-32.024151070
O	-8.043206764	-6.665008532	-31.733242335
N	-10.124251782	-6.298898528	-32.609702874
C	-9.823183061	-5.014496986	-33.244852012
H	-10.721744968	-4.386782009	-33.242883988
H	-9.524393983	-5.204590004	-34.287202001
H	-6.634856057	-8.496379034	-35.345822945
C	-6.877446846	-8.731616926	-34.300434140
C	-5.636857096	-8.999300988	-33.465042893
O	-5.743020206	-9.258281591	-32.249040902
H	-7.531247041	-9.610727047	-34.252218976
N	-4.438885823	-8.979107776	-34.093181373
C	-3.260094904	-9.541344992	-33.464299182
C	-2.196803066	-8.536883027	-33.037758832
O	-1.048026034	-8.934485384	-32.771538281
C	-2.654617684	-10.640451782	-34.358585445
O	-3.537688178	-11.755182782	-34.511846840
N	-2.568619275	-7.252466113	-32.897626576
C	-1.620981964	-6.229227966	-32.487174083
H	-1.491963017	-6.154970028	-31.397095980
H	-1.985815015	-5.271092003	-32.873731988
H	-12.461799020	-11.734243991	-31.272652974
C	-12.324101935	-12.721649034	-31.732007062
C	-11.957925090	-13.787976952	-30.724054934
O	-12.492486697	-14.910302111	-30.781448890
H	-11.531087736	-12.626348030	-32.489726914
N	-11.052909985	-13.469448686	-29.756348934
C	-10.821918888	-14.384823052	-28.638755041
C	-10.975026105	-13.594969965	-27.344677973
O	-10.195976216	-12.658308306	-27.064184827
C	-9.499263885	-15.196346028	-28.686118892
C	-9.366448886	-15.925058327	-30.037429675
C	-9.468777764	-16.177759583	-27.506530592
C	-8.005927037	-16.583325290	-30.282562307
N	-12.004256349	-13.939358695	-26.551154929
C	-12.237610931	-13.280757999	-25.283069008
C	-11.202859164	-13.696490221	-24.226168424
O	-10.911432762	-14.884794608	-24.041623020
H	-13.214770046	-13.615247006	-24.911789010
N	-10.685509895	-12.673855106	-23.505987662
C	-9.887656921	-12.869598575	-22.316417785

C	-10.350416706	-11.836818680	-21.274458816
O	-10.901536942	-10.773761103	-21.611126915
C	-8.359084891	-12.656900385	-22.543576785
C	-7.727953405	-13.485582516	-23.608621759
C	-7.571399589	-13.229759878	-24.949024308
N	-7.073277999	-14.686519012	-23.398538704
C	-6.540774233	-15.110420934	-24.565646091
N	-6.828173971	-14.240307300	-25.539128335
N	-10.063354259	-12.150351531	-20.001074133
C	-10.362962913	-11.260640992	-18.897075925
C	-9.689314067	-9.902912012	-19.046614065
H	-9.989707044	-11.768594991	-17.999013027
C	-8.165190024	-4.104271640	-25.627915824
C	-8.844191891	-5.273296026	-24.993549939
C	-9.333688080	-5.385292959	-23.719741044
C	-9.344313725	-6.415680730	-25.722742966
C	-10.107047429	-7.189589317	-24.803874221
C	-9.279319468	-6.814633954	-27.066576185
N	-10.064261434	-6.533848852	-23.584667739
C	-10.788901773	-8.345006316	-25.180684335
C	-9.976167942	-7.965009760	-27.457845595
C	-10.713391209	-8.720872333	-26.530776234
C	-5.717886969	-7.891400994	-20.592886986
C	-5.045483162	-8.977081810	-21.439538367
C	-5.293267747	-8.879183230	-22.952540181
H	-5.410174341	-9.947187443	-21.064506111
C	-6.770605311	-8.846845373	-23.348442399
N	-3.880460661	-4.945582056	-26.375185110
C	-4.014020783	-4.861950834	-27.812787010
H	-4.117210139	-3.802590088	-28.100561979
C	-5.080066231	-5.825297100	-28.374858962
C	-4.564417427	-7.240251390	-28.225196795
C	-4.631066511	-7.907906503	-26.991450665
C	-3.851817743	-7.850258854	-29.271929666
C	-3.943497719	-9.109106315	-26.788545943
C	-3.191452497	-9.067306163	-29.085018530
C	-3.212089040	-9.690281345	-27.831356538
Cu	-6.069069605	-14.089709550	-27.341153308
H	-8.698246505	-11.766716784	-28.034694436
H	-6.699738170	-10.329691659	-29.759938700
H	-8.033558037	-13.082001547	-29.986406550
H	-7.482225743	-12.226860723	-31.418968974
H	-4.899091162	-12.358954487	-32.154005870
H	-3.463759971	-14.558666934	-28.839694624
H	-7.573316308	-9.074440954	-31.421298024
H	-9.895305451	-8.967074664	-32.749783312
H	-10.503684719	-8.558742424	-31.138677906
H	-10.998448399	-6.746507139	-32.876403627
H	-9.012039502	-4.515609496	-32.706186423
H	-7.413072181	-7.878615853	-33.865780081
H	-4.414287329	-8.781786212	-35.092118274
H	-3.612108420	-10.015761698	-32.533298917
H	-2.378954243	-10.215818738	-35.340522506
H	-1.745451958	-11.018263806	-33.879054036
H	-3.513980678	-6.981919589	-33.154204070
H	-0.640006204	-6.443483121	-32.929905110
H	-13.247363573	-13.026408672	-32.235454457
H	-10.647797895	-12.525786455	-29.732713291
H	-11.636683885	-15.122673261	-28.709729859
H	-8.656005243	-14.488980310	-28.575206758
H	-10.161584256	-16.686750395	-30.099861721
H	-9.562269046	-15.209411125	-30.850268543

H	-10.321180522	-16.873789016	-27.563320281
H	-9.518304963	-15.662777416	-26.536988581
H	-8.548807221	-16.776804300	-27.513117252
H	-7.772716770	-17.348922389	-29.527993672
H	-7.198988512	-15.833852888	-30.277509764
H	-7.987938945	-17.078078871	-31.264498877
H	-12.592860288	-14.725042946	-26.811606849
H	-12.261034102	-12.191601345	-25.420621957
H	-11.009535078	-11.720238493	-23.669441415
H	-10.064900931	-13.891855142	-21.952588089
H	-8.206998219	-11.600878731	-22.808145172
H	-7.842050837	-12.828319712	-21.588275794
H	-7.961636986	-12.391321753	-25.509835099
H	-5.951979827	-16.016168208	-24.658721849
H	-9.624780635	-13.042972445	-19.792145572
H	-11.452656904	-11.145169887	-18.787530438
H	-9.211638490	-4.723554391	-22.867517057
H	-8.714438877	-6.234799643	-27.798325327
H	-10.484279414	-6.908877226	-22.711057790
H	-11.378578068	-8.919786290	-24.465826052
H	-9.962578537	-8.271402399	-28.503987043
H	-11.247362875	-9.610111127	-26.867566110
H	-3.959096331	-8.961792358	-21.249234682
H	-4.789692489	-7.983674978	-23.351669306
H	-4.799504554	-9.739731049	-23.432066369
H	-7.268468608	-7.935645375	-22.986508445
H	-7.311389186	-9.707549991	-22.927807689
H	-6.887580739	-8.876449599	-24.441060885
H	-3.036884265	-5.160761460	-28.218631765
H	-6.035148290	-5.688569006	-27.845392568
H	-5.248752895	-5.588862328	-29.435405664
H	-5.191435319	-7.463692412	-26.167321812
H	-3.797110648	-7.350904743	-30.241983650
H	-3.961346655	-9.578861637	-25.803022671
H	-2.634493162	-9.515276398	-29.909655212
H	-2.646109165	-10.610519637	-27.666475666
H	-2.934378704	-13.632623387	-31.146281572
H	-6.979001668	-15.169965427	-22.507344336
H	-8.162803642	-10.201377919	-27.825105188
H	-4.286949537	-11.474816461	-35.068708334
N	-10.472318208	-8.839576177	-18.766267167
C	-10.118204946	-7.498699001	-19.194366948
H	-10.022163032	-6.809331996	-18.339386014
C	-11.195214375	-6.966185962	-20.133425550
H	-10.965196405	-5.928766589	-20.412469410
O	-11.280430493	-7.711358399	-21.362353566
O	-8.513357859	-9.812313249	-19.425312468
H	-11.437253489	-9.009039339	-18.488449241
H	-12.175777697	-6.973501389	-19.624748237
H	-9.144066406	-7.550488359	-19.695559374
H	-11.215759929	-8.674975278	-21.179039791
H	-7.000726943	-2.350805972	-25.167168000
C	-7.552381193	-3.132925077	-24.622741019
C	-6.547009859	-3.871510941	-23.756511996
O	-5.747648561	-4.697160420	-24.233704469
N	-6.567499424	-3.581424531	-22.438425727
C	-5.630934010	-4.177288025	-21.505242028
C	-6.145718017	-5.486159997	-20.909637987
O	-7.266398667	-5.537570238	-20.373280909
H	-5.472591993	-3.445790988	-20.701967993
N	-5.330461305	-6.552371803	-21.027687590
H	-8.890581729	-3.566021064	-26.262714905

H	-7.382302396	-4.464423851	-26.312184101
H	-8.318198348	-2.636806155	-24.010056294
H	-4.674464713	-4.325437050	-22.022787748
H	-4.424311394	-6.427784259	-21.472939663
H	-1.789516968	-5.874400023	-23.964633004
C	-2.664450045	-5.300128898	-24.289385965
C	-2.712922068	-5.311856148	-25.809406034
O	-1.695000859	-5.604704176	-26.469734274
H	-2.542204086	-4.263083967	-23.940378024
H	-3.582358551	-5.693590424	-23.837337943
H	-4.681171767	-4.745515118	-25.761015050
H	-7.261043320	-2.930321820	-22.082036944
H	-5.431884020	-8.037666004	-19.538078010
H	-6.811840832	-7.954026965	-20.645660728
O	-5.527011125	-16.184710960	-27.595955832
O	-5.531569369	-11.936742226	-26.796501110
H	-5.705879480	-16.442925869	-28.521628092
H	-7.058480844	-11.430556134	-27.683639613
H	-5.430716608	-11.746767372	-25.843452730
H	-6.057612447	-16.810062907	-27.065536108
H	-4.763301956	-11.514647950	-27.236952512

C2-i

N	-7.867467150	-11.123197160	-28.207025516
C	-7.559463700	-10.971312543	-29.662497544
C	-8.789194914	-10.309511808	-30.310150334
O	-9.933246557	-10.728407620	-30.049736861
C	-7.253330785	-12.336416176	-30.333153358
C	-5.849042164	-12.864015718	-30.248215833
C	-4.903754156	-12.805929525	-31.250089895
N	-5.295004007	-13.583423959	-29.186025177
C	-4.053536694	-13.924938359	-29.544520210
N	-3.792974542	-13.469360212	-30.785031160
N	-8.540953171	-9.257305726	-31.107389070
C	-9.610709916	-8.504724473	-31.735299891
C	-9.173088091	-7.058967303	-32.013652397
O	-8.032113158	-6.655521007	-31.743389447
N	-10.120720639	-6.294998749	-32.602053069
C	-9.823183086	-5.014496947	-33.244852005
H	-10.721744947	-4.386782027	-33.242883985
H	-9.524393977	-5.204590014	-34.287202012
H	-6.634855985	-8.496378964	-35.345822983
C	-6.877447037	-8.731617085	-34.300434021
C	-5.636856970	-8.999300987	-33.465043034
O	-5.739379540	-9.252418718	-32.248556224
H	-7.531246989	-9.610726975	-34.252218992
N	-4.438651813	-8.981028336	-34.095304843
C	-3.260095057	-9.541345007	-33.464298905
C	-2.196802946	-8.536882984	-33.037759092
O	-1.047140219	-8.933790730	-32.773880234
C	-2.653198976	-10.641211526	-34.356512529
O	-3.537168538	-11.754907372	-34.513328317
N	-2.568835624	-7.252611141	-32.896888755
C	-1.620982047	-6.229228032	-32.487173949
H	-1.491962983	-6.154969982	-31.397096015
H	-1.985814983	-5.271091995	-32.873732014
H	-12.461799040	-11.734243995	-31.272652973
C	-12.324101866	-12.721649041	-31.732007072
C	-11.957925195	-13.787976941	-30.724054925
O	-12.494932202	-14.909913173	-30.781583888

H	-11.531227972	-12.625661260	-32.489800086
N	-11.054739190	-13.469609038	-29.756070328
C	-10.821918786	-14.384823005	-28.638754975
C	-10.975026151	-13.594970004	-27.344678057
O	-10.191228487	-12.668297382	-27.054402949
C	-9.490448144	-15.181842192	-28.685242661
C	-9.349063233	-15.911749663	-30.034862851
C	-9.445974603	-16.160245130	-27.503519069
C	-7.979188287	-16.551445596	-30.277121709
N	-12.009261822	-13.937150405	-26.553751984
C	-12.237610920	-13.280758032	-25.283068976
C	-11.204465692	-13.698523349	-24.227115866
O	-10.922766897	-14.888838922	-24.037893504
H	-13.214770049	-13.615246971	-24.911789029
N	-10.679814796	-12.678062702	-23.509118243
C	-9.895615363	-12.882134268	-22.311717625
C	-10.358937130	-11.847188250	-21.272437272
O	-10.923062640	-10.791023934	-21.608068310
C	-8.359774766	-12.699155876	-22.517542262
C	-7.741515101	-13.500515710	-23.609382211
C	-7.274493005	-13.087057546	-24.832272860
N	-7.455931335	-14.854586749	-23.556607642
C	-6.849021297	-15.219242893	-24.707261503
N	-6.721838796	-14.159146255	-25.511062848
N	-10.061025684	-12.152158657	-19.998934854
C	-10.362962863	-11.260640983	-18.897075940
C	-9.689314108	-9.902912011	-19.046614069
H	-9.989707061	-11.768594998	-17.999013014
C	-8.162736254	-4.102068336	-25.633342571
C	-8.844191885	-5.273296051	-24.993549927
C	-9.333688089	-5.385292944	-23.719741052
C	-9.340197977	-6.417181513	-25.719409282
C	-10.100103336	-7.201118449	-24.797399044
C	-9.264198093	-6.827372379	-27.063737152
N	-10.072251184	-6.550042908	-23.586976511
C	-10.756466609	-8.378924851	-25.181849262
C	-9.931234797	-7.988699989	-27.451507361
C	-10.665375382	-8.758482257	-26.520447108
C	-5.717887016	-7.891400981	-20.592886957
C	-5.038041219	-8.976040404	-21.435593207
C	-5.280428694	-8.883627396	-22.949596648
H	-5.399149519	-9.947210909	-21.059794971
C	-6.756736075	-8.867707510	-23.350034047
N	-3.884985867	-4.960037711	-26.375799088
C	-4.014021035	-4.861951012	-27.812786975
H	-4.117209986	-3.802589987	-28.100562011
C	-5.091897865	-5.798591815	-28.392954137
C	-4.615653962	-7.227563119	-28.262407005
C	-4.804911175	-7.952498408	-27.075324648
C	-3.832577884	-7.804477343	-29.276648660
C	-4.186709944	-9.193237246	-26.887736545
C	-3.229602502	-9.052792098	-29.103012560
C	-3.390150188	-9.744880233	-27.897404881
Cu	-5.916505480	-13.913928833	-27.318156554
H	-8.685084533	-11.749352911	-28.041774498
H	-6.687614688	-10.308768471	-29.740964607
H	-7.967535481	-13.082416016	-29.955819917
H	-7.473765656	-12.218721085	-31.403497411
H	-4.934103041	-12.368409978	-32.242182675
H	-3.346998871	-14.484431905	-28.941490085
H	-7.569043620	-9.027480721	-31.367833244
H	-9.915390549	-8.966631464	-32.690416016

H	-10.491793885	-8.520550152	-31.077905188
H	-11.001072185	-6.740484966	-32.850989047
H	-9.011823458	-4.511914324	-32.709593978
H	-7.413881514	-7.879892645	-33.864360031
H	-4.414353096	-8.787358377	-35.094898207
H	-3.613185305	-10.013966679	-32.532765059
H	-2.373948514	-10.216770791	-35.337635192
H	-1.746098062	-11.020797796	-33.874583452
H	-3.515621468	-6.983038146	-33.149300826
H	-0.640169851	-6.443952799	-32.930005190
H	-13.247298414	-13.026662837	-32.235516291
H	-10.635316048	-12.531023612	-29.740956317
H	-11.632138250	-15.127263131	-28.709874558
H	-8.657248667	-14.463236319	-28.576734249
H	-10.132027619	-16.686036804	-30.096150782
H	-9.554242041	-15.201171277	-30.849632719
H	-10.284770172	-16.872489082	-27.562230716
H	-9.510260545	-15.643508719	-26.535734928
H	-8.513705882	-16.740074263	-27.503344913
H	-7.740268281	-17.316037198	-29.522540960
H	-7.183735075	-15.788139463	-30.272950556
H	-7.947957522	-17.045364223	-31.258987228
H	-12.602773661	-14.718101154	-26.816811762
H	-12.259756262	-12.191205214	-25.418238452
H	-10.995761206	-11.722316598	-23.675894408
H	-10.093907353	-13.901783250	-21.950384760
H	-8.170454314	-11.641381068	-22.746980764
H	-7.862200405	-12.917775042	-21.560451709
H	-7.272615289	-12.089198923	-25.249037877
H	-6.517043767	-16.232849176	-24.900918882
H	-9.611858702	-13.038944452	-19.788652233
H	-11.452819261	-11.145790456	-18.788661025
H	-9.220465462	-4.724597028	-22.865459492
H	-8.707711081	-6.239570083	-27.796746197
H	-10.467345053	-6.935110098	-22.715994971
H	-11.336327055	-8.964147633	-24.466213065
H	-9.904814928	-8.302367088	-28.495874708
H	-11.178412319	-9.660168847	-26.858615190
H	-3.952439487	-8.954807696	-21.240790610
H	-4.784099470	-7.984352937	-23.349494671
H	-4.777010622	-9.740423278	-23.425618639
H	-7.265040709	-7.958663079	-22.997041046
H	-7.288725535	-9.731085307	-22.923687261
H	-6.870188805	-8.907862247	-24.442564722
H	-3.039428437	-5.165327747	-28.220912540
H	-6.049940851	-5.646988049	-27.872944763
H	-5.243812774	-5.541029019	-29.451547319
H	-5.419734706	-7.529771298	-26.278316591
H	-3.680119265	-7.255886425	-30.209008557
H	-4.321419243	-9.728388257	-25.946684623
H	-2.618061557	-9.476847067	-29.901450856
H	-2.887158173	-10.702334540	-27.743601183
H	-2.921657743	-13.608989162	-31.291754057
H	-7.662512883	-15.482039103	-22.782247660
H	-8.109392138	-10.211309689	-27.794714825
H	-4.290679979	-11.469268851	-35.061814833
N	-10.468352920	-8.839062739	-18.758956547
C	-10.118204898	-7.498699024	-19.194366978
H	-10.022163047	-6.809331982	-18.339385994
C	-11.209036546	-6.974254564	-20.123277158
H	-10.976309144	-5.943346733	-20.424227572
O	-11.325432137	-7.739704573	-21.334662545

O	-8.515402310	-9.812954973	-19.431798380
H	-11.432294445	-9.008078404	-18.477283721
H	-12.178174439	-6.963039409	-19.592117916
H	-9.148085508	-7.549836680	-19.702729783
H	-11.258734521	-8.699293159	-21.133876124
H	-7.000726971	-2.350805982	-25.167168001
C	-7.552381102	-3.132925033	-24.622741018
C	-6.547009934	-3.871510986	-23.756512012
O	-5.730426512	-4.680550028	-24.233217914
N	-6.570749117	-3.584960523	-22.436882911
C	-5.630933996	-4.177287999	-21.505241995
C	-6.145718010	-5.486160014	-20.909638010
O	-7.263667096	-5.536022742	-20.367186831
H	-5.472591996	-3.445790991	-20.701968001
N	-5.331787777	-6.552948510	-21.029423992
H	-8.874281489	-3.553739742	-26.274320339
H	-7.367153482	-4.459316291	-26.305072260
H	-8.318979044	-2.640142906	-24.007970973
H	-4.676055994	-4.324939444	-22.025636469
H	-4.429282220	-6.430394435	-21.482458985
H	-1.789516988	-5.874400006	-23.964632996
C	-2.664450033	-5.300128989	-24.289385996
C	-2.712921981	-5.311855998	-25.809406005
O	-1.691381619	-5.594367974	-26.469348644
H	-2.540682042	-4.262831037	-23.941474041
H	-3.582393592	-5.691735805	-23.836269743
H	-4.683826340	-4.754379244	-25.759911790
H	-7.281300653	-2.954632661	-22.077237584
H	-5.431883999	-8.037666008	-19.538078028
H	-6.811465198	-7.957853491	-20.648445987
O	-5.559659481	-15.952413374	-27.674137194
O	-5.763942999	-12.016779211	-26.960241417
H	-5.912735064	-16.185929026	-28.556678958
H	-6.975321989	-11.510034908	-27.655030906
H	-5.034222525	-11.665203280	-27.507216103
H	-5.949002089	-16.613885304	-27.071190643

C2-ii

N	-7.529245613	-11.094633434	-28.389675056
C	-7.452331601	-11.068852906	-29.867057230
C	-8.717583890	-10.379717782	-30.414200931
O	-9.855618160	-10.807868697	-30.131749005
C	-7.323687508	-12.498305681	-30.449913799
C	-5.967372918	-13.141218596	-30.394716347
C	-5.032308093	-13.145930988	-31.409483472
N	-5.463923122	-13.915589032	-29.346805022
C	-4.264271672	-14.357797458	-29.729633156
N	-3.975956293	-13.908205261	-30.968796528
N	-8.516613664	-9.263819525	-31.140927859
C	-9.621521138	-8.472586440	-31.649303886
C	-9.168705226	-7.046161530	-31.991183747
O	-8.022773556	-6.641465336	-31.743308233
N	-10.115586790	-6.291663472	-32.594573732
C	-9.823182904	-5.014497041	-33.244851949
H	-10.721745062	-4.386781972	-33.242884030
H	-9.524394028	-5.204589986	-34.287202019
H	-6.634855976	-8.496378918	-35.345823020
C	-6.877447048	-8.731617198	-34.300433925
C	-5.636856929	-8.999300914	-33.465043122
O	-5.734835713	-9.247680903	-32.247665266

H	-7.531246997	-9.610726946	-34.252219007
N	-4.438932338	-8.982270679	-34.096253044
C	-3.260095213	-9.541345132	-33.464298637
C	-2.196802839	-8.536882836	-33.037759350
O	-1.045728086	-8.932624056	-32.778451192
C	-2.647997917	-10.634635393	-34.362342203
O	-3.529868198	-11.746272177	-34.538894848
N	-2.569037211	-7.252673796	-32.896044947
C	-1.620981976	-6.229228184	-32.487173717
H	-1.491963012	-6.154969948	-31.397096134
H	-1.985815018	-5.271091943	-32.873732060
H	-12.461798992	-11.734244008	-31.272653006
C	-12.324102039	-12.721648951	-31.732007018
C	-11.957924946	-13.787977051	-30.724054964
O	-12.515788600	-14.901077142	-30.771240806
H	-11.532242790	-12.623212434	-32.490600381
N	-11.042926627	-13.475826954	-29.764532230
C	-10.821919058	-14.384822995	-28.638755097
C	-10.975025996	-13.594969994	-27.344677863
O	-10.198401599	-12.667396647	-27.051508086
C	-9.517079261	-15.226387977	-28.676070847
C	-9.384073324	-15.933422003	-30.039274499
C	-9.533788127	-16.227117758	-27.512084036
C	-8.104493009	-16.753134596	-30.228358855
N	-12.011541223	-13.941951471	-26.551870558
C	-12.237610823	-13.280757912	-25.283069146
C	-11.206230996	-13.689742767	-24.223026479
O	-10.919525424	-14.877569899	-24.024527104
H	-13.214770130	-13.615247072	-24.911788928
N	-10.691319581	-12.661734649	-23.509086675
C	-9.881030443	-12.847178730	-22.326961434
C	-10.350762351	-11.828730043	-21.275211513
O	-10.916042531	-10.769324711	-21.599622570
C	-8.357098153	-12.618783744	-22.573608451
C	-7.740299228	-13.438322041	-23.653449756
C	-7.399403556	-13.086738244	-24.938367911
N	-7.332148371	-14.756197687	-23.526210187
C	-6.774278271	-15.157907909	-24.692059958
N	-6.798506647	-14.158665566	-25.573112784
N	-10.060365346	-12.147860081	-20.002893922
C	-10.362962992	-11.260640976	-18.897076047
C	-9.689314022	-9.902912014	-19.046613976
H	-9.989706991	-11.768595011	-17.999012967
C	-8.171852324	-4.096986094	-25.633709904
C	-8.844191993	-5.273295990	-24.993550040
C	-9.333687988	-5.385293016	-23.719740968
C	-9.285335482	-6.448326239	-25.703507944
C	-10.023595570	-7.244799521	-24.775111533
C	-9.159728261	-6.890851418	-27.034280388
N	-10.032657394	-6.572968294	-23.575520427
C	-10.617096054	-8.461115579	-25.141695659
C	-9.756232200	-8.095648466	-27.402078457
C	-10.475759024	-8.872971392	-26.466218064
C	-5.717887144	-7.891400987	-20.592887192
C	-5.027430407	-8.970625636	-21.435528738
C	-5.240697737	-8.859593776	-22.952699959
H	-5.398206934	-9.944892631	-21.077734110
C	-6.709350539	-8.850008563	-23.379819853
N	-3.886186840	-4.963603174	-26.375811042
C	-4.014021247	-4.861950954	-27.812786922
H	-4.117209856	-3.802590042	-28.100562026
C	-5.076408383	-5.810518695	-28.400085260

C	-4.556579249	-7.225831778	-28.289579114
C	-4.740661930	-7.981338163	-27.120887760
C	-3.723327687	-7.745588195	-29.293387532
C	-4.072867580	-9.197076339	-26.943177605
C	-3.065749984	-8.967446875	-29.126058245
C	-3.225750161	-9.690454612	-27.940108819
Cu	-5.962767307	-14.157494094	-27.395615802
H	-8.383466486	-11.594964806	-28.098527909
H	-6.568526175	-10.480970262	-30.149654679
H	-8.066284986	-13.144676677	-29.961391028
H	-7.609716796	-12.447166760	-31.510575575
H	-5.037601114	-12.689230761	-32.393318131
H	-3.602374967	-14.985211507	-29.143000876
H	-7.558451454	-9.024127686	-31.439657057
H	-10.062164925	-8.931937884	-32.551026402
H	-10.419739752	-8.440617009	-30.892268259
H	-10.998098379	-6.738816248	-32.831834408
H	-9.011303266	-4.508200057	-32.713429171
H	-7.415118755	-7.880675060	-33.863843513
H	-4.414716789	-8.790446945	-35.096188722
H	-3.612566853	-10.018584324	-32.535061738
H	-2.360293840	-10.199628258	-35.336623578
H	-1.745105489	-11.019702583	-33.876996356
H	-3.517878130	-6.984261693	-33.141916115
H	-0.640402049	-6.444514756	-32.930167845
H	-13.247552628	-13.027010654	-32.234837526
H	-10.595921266	-12.548050166	-29.768971459
H	-11.646736282	-15.111699048	-28.710422352
H	-8.654158626	-14.550061432	-28.535133035
H	-10.262718265	-16.585338532	-30.178296695
H	-9.438858144	-15.174876113	-30.835597232
H	-10.377330238	-16.929158458	-27.614703013
H	-9.631422583	-15.723817022	-26.539534358
H	-8.604464573	-16.810620101	-27.483131510
H	-8.032232337	-17.584190800	-29.512265401
H	-7.205847650	-16.128850794	-30.110710720
H	-8.076357549	-17.187122590	-31.238893390
H	-12.603733745	-14.724878504	-26.811158440
H	-12.261314280	-12.191667674	-25.422840501
H	-10.999763955	-11.707597424	-23.695163642
H	-10.042333864	-13.873223189	-21.966088769
H	-8.215706541	-11.561905729	-22.839897277
H	-7.828350194	-12.787457074	-21.623123664
H	-7.571250905	-12.138458917	-25.433222271
H	-6.367909059	-16.146190993	-24.873758676
H	-9.602385673	-13.031923992	-19.799541070
H	-11.452848191	-11.146037060	-18.789087759
H	-9.247966441	-4.709235979	-22.874403010
H	-8.612211180	-6.298648189	-27.770558277
H	-10.434217897	-6.953256187	-22.705486833
H	-11.180707932	-9.056481351	-24.421305783
H	-9.676674474	-8.444097685	-28.432401741
H	-10.930775492	-9.811359061	-26.786927008
H	-3.945566687	-8.954525272	-21.220533251
H	-4.743649071	-7.951491181	-23.331117647
H	-4.721576288	-9.705690894	-23.431056581
H	-7.235421113	-7.956798615	-23.012699730
H	-7.235337849	-9.731320005	-22.984354860
H	-6.804200216	-8.863075995	-24.474763843
H	-3.035478502	-5.157052294	-28.217830302
H	-6.034157414	-5.690772242	-27.871493652
H	-5.240967442	-5.541977136	-29.454150561

H	-5.394704672	-7.603011855	-26.332541973
H	-3.573395068	-7.171471694	-30.210663384
H	-4.208304688	-9.757528534	-26.016675504
H	-2.416220606	-9.347113940	-29.917019877
H	-2.703317705	-10.637435225	-27.797634413
H	-3.126198401	-14.114007599	-31.488356644
H	-7.427796484	-15.334565113	-22.694429938
H	-7.621921318	-10.136624778	-28.033105205
H	-4.285934910	-11.451643360	-35.079031198
N	-10.467811286	-8.838871894	-18.757608315
C	-10.118204981	-7.498699001	-19.194367040
H	-10.022162999	-6.809331996	-18.339385978
C	-11.208722880	-6.976101096	-20.124512873
H	-10.976723144	-5.945222460	-20.426337038
O	-11.323469040	-7.743213392	-21.334703998
O	-8.515328681	-9.812845066	-19.431456841
H	-11.432422724	-9.008216892	-18.478459312
H	-12.178246141	-6.965041479	-19.593911970
H	-9.147900195	-7.549995851	-19.702439858
H	-11.252322743	-8.702870614	-21.133687255
H	-7.000727013	-2.350805993	-25.167168006
C	-7.552380974	-3.132925032	-24.622740995
C	-6.547009992	-3.871510984	-23.756512025
O	-5.730627700	-4.680634642	-24.233254397
N	-6.570413237	-3.584512820	-22.436892878
C	-5.630934042	-4.177288009	-21.505242084
C	-6.145717950	-5.486160016	-20.909637918
O	-7.264636701	-5.535912740	-20.368840694
H	-5.472591995	-3.445790981	-20.701967955
N	-5.331106361	-6.552027996	-21.026252882
H	-8.888494515	-3.543464347	-26.264602917
H	-7.383041019	-4.450104928	-26.315512111
H	-8.315764605	-2.637500535	-24.005823466
H	-4.676042430	-4.324974663	-22.025630216
H	-4.429203025	-6.430025111	-21.480510714
H	-1.789516963	-5.874399970	-23.964633001
C	-2.664450085	-5.300129071	-24.289385999
C	-2.712921893	-5.311855939	-25.809406003
O	-1.689000814	-5.587914862	-26.468391519
H	-2.539537731	-4.262600827	-23.942410919
H	-3.582456022	-5.690431460	-23.835351544
H	-4.685125954	-4.759680566	-25.759594712
H	-7.282877894	-2.956621698	-22.076903400
H	-5.431883925	-8.037666002	-19.538077863
H	-6.810899154	-7.960564266	-20.652737351
O	-5.760808916	-16.048378079	-27.365032714
O	-5.489481039	-12.186820441	-27.213114317
H	-5.679130659	-16.356223983	-28.285365674
H	-6.314902453	-11.689030223	-27.681530399
H	-5.467544006	-11.881544463	-26.287742782

C3-i

N	-7.757874064	-11.256431880	-28.234814314
C	-7.363597262	-10.909593081	-29.628240833
C	-8.611727796	-10.262530817	-30.277304744
O	-9.747459793	-10.690165578	-29.986018358
C	-6.914444092	-12.181496895	-30.396691364
C	-5.481128349	-12.638359598	-30.262214327
C	-4.635497062	-12.759897427	-31.346109411
N	-4.796076524	-13.106642732	-29.129330032

C	-3.592616095	-13.499634951	-29.550909327
N	-3.463224967	-13.302122873	-30.878810606
N	-8.398525625	-9.217274997	-31.093227148
C	-9.494831775	-8.493399594	-31.714274004
C	-9.109731969	-7.032133841	-32.002679884
O	-7.981661691	-6.588228562	-31.743428973
N	-10.086732089	-6.297634921	-32.588677869
C	-9.823182933	-5.014497012	-33.244851878
H	-10.721745031	-4.386781997	-33.242884050
H	-9.524394028	-5.204590000	-34.287202063
H	-6.634856013	-8.496379080	-35.345823048
C	-6.877446980	-8.731616777	-34.300433852
C	-5.636857005	-8.999301094	-33.465043086
O	-5.727472267	-9.232450909	-32.243794089
H	-7.531247009	-9.610727065	-34.252219044
N	-4.440507092	-8.987663208	-34.098268236
C	-3.260094995	-9.541344998	-33.464298845
C	-2.196803013	-8.536882972	-33.037759147
O	-1.046184609	-8.933469005	-32.778051103
C	-2.645086100	-10.632704552	-34.363428436
O	-3.525530303	-11.744775012	-34.545051667
N	-2.569032780	-7.252904476	-32.895702022
C	-1.620981971	-6.229228027	-32.487173964
H	-1.491963014	-6.154969978	-31.397095999
H	-1.985815004	-5.271092012	-32.873731999
H	-12.461799039	-11.734243984	-31.272652965
C	-12.324101922	-12.721649036	-31.732007067
C	-11.957925061	-13.787976972	-30.724054921
O	-12.510912258	-14.902550383	-30.772163872
H	-11.532498885	-12.625088618	-32.491230360
N	-11.047679183	-13.473093958	-29.761750093
C	-10.821918976	-14.384823053	-28.638755148
C	-10.975025998	-13.594969964	-27.344677891
O	-10.230469540	-12.637176101	-27.065128401
C	-9.516749222	-15.215781255	-28.717327950
C	-9.464824846	-16.028945413	-30.025081912
C	-9.410834160	-16.126020140	-27.485388524
C	-8.116308558	-16.711063297	-30.282305809
N	-11.989133490	-13.968790655	-26.534278846
C	-12.237611031	-13.280757958	-25.283068993
C	-11.184550029	-13.635594691	-24.212021887
O	-10.894456280	-14.812525678	-23.963163123
H	-13.214769977	-13.615247028	-24.911789014
N	-10.635275702	-12.572832773	-23.569467630
C	-9.803409275	-12.686758674	-22.387689024
C	-10.384501708	-11.777679989	-21.286315970
O	-11.025831237	-10.750094063	-21.566947810
C	-8.333759898	-12.205041794	-22.590491649
C	-7.454367148	-12.950578667	-23.534694352
C	-6.825024069	-12.510893388	-24.677589663
N	-6.965973077	-14.230770576	-23.333022902
C	-6.089074123	-14.525916171	-24.325778941
N	-5.979824077	-13.494023856	-25.159860833
N	-10.073935912	-12.129523577	-20.024203383
C	-10.362962944	-11.260640975	-18.897076050
C	-9.689314018	-9.902912027	-19.046613983
H	-9.989707022	-11.768595009	-17.999012972
C	-8.163952519	-4.101407769	-25.633366058
C	-8.844192032	-5.273295987	-24.993550120
C	-9.333687961	-5.385293039	-23.719740915
C	-9.342675257	-6.416557444	-25.719474186
C	-10.102575837	-7.199637939	-24.797269721

C	-9.264124070	-6.830304815	-27.063041844
N	-10.074691605	-6.548010149	-23.586744250
C	-10.756029824	-8.379689941	-25.179887241
C	-9.924402881	-7.995753061	-27.448124476
C	-10.658817684	-8.763853001	-26.516692821
C	-5.717886936	-7.891401000	-20.592887034
C	-5.059790678	-8.972271147	-21.455329127
C	-5.285684587	-8.818662040	-22.968150972
H	-5.461737077	-9.940722724	-21.116435343
C	-6.751367051	-8.630950213	-23.365354245
N	-3.889829678	-4.975319175	-26.376404351
C	-4.014020998	-4.861951086	-27.812787015
H	-4.117210004	-3.802589949	-28.100561995
C	-5.119924581	-5.752069683	-28.407957521
C	-4.756306407	-7.207457158	-28.233345057
C	-5.243760865	-7.953080567	-27.150328128
C	-3.816426609	-7.807380435	-29.088449091
C	-4.781450727	-9.250993083	-26.908294455
C	-3.354828235	-9.104548390	-28.853425254
C	-3.828166942	-9.826499093	-27.752530949
Cu	-5.213033086	-13.385112042	-27.092486353
H	-7.381462656	-12.349435974	-27.911046325
H	-6.541544367	-10.184255489	-29.591789429
H	-7.602258932	-12.999435725	-30.132904241
H	-7.060128199	-11.989050262	-31.468896782
H	-4.769678552	-12.509067351	-32.393253483
H	-2.817731377	-13.940509656	-28.933898657
H	-7.432654405	-9.007645277	-31.400652211
H	-9.795538326	-8.964930871	-32.666215948
H	-10.369450905	-8.536074502	-31.049592607
H	-10.951934177	-6.772675302	-32.836434068
H	-9.012163404	-4.499343201	-32.720413769
H	-7.413219820	-7.878837340	-33.865809829
H	-4.417723942	-8.802889870	-35.099600982
H	-3.611308009	-10.018852046	-32.534887550
H	-2.355555634	-10.195156954	-35.335982875
H	-1.742870144	-11.017915469	-33.877131660
H	-3.519517799	-6.985064623	-33.135642226
H	-0.640564808	-6.444852239	-32.930272274
H	-13.248239244	-13.025593954	-32.234291779
H	-10.612820023	-12.544141674	-29.751832554
H	-11.651420057	-15.107885551	-28.703645147
H	-8.665526430	-14.517774320	-28.709789231
H	-10.268907164	-16.784491029	-30.010096032
H	-9.688077284	-15.359435896	-30.869720205
H	-10.267066715	-16.818544893	-27.437674936
H	-9.390073437	-15.552286792	-26.547414534
H	-8.493328230	-16.728501787	-27.519131212
H	-7.877730672	-17.461727676	-29.515140744
H	-7.296405878	-15.976707161	-30.298160739
H	-8.122182048	-17.226199032	-31.254225384
H	-12.564325522	-14.768863659	-26.779320517
H	-12.276074643	-12.196563665	-25.455930266
H	-10.961259718	-11.634644333	-23.798438716
H	-9.818068725	-13.739075253	-22.070621868
H	-8.372077562	-11.163437416	-22.937159052
H	-7.859342448	-12.189839059	-21.596458391
H	-6.919993717	-11.546584998	-25.164464637
H	-5.575564497	-15.477831130	-24.409661534
H	-9.526876005	-12.969895623	-19.859144856
H	-11.451564129	-11.144208291	-18.781921923
H	-9.218550514	-4.725122706	-22.865266416

H	-8.705191587	-6.244339990	-27.795766026
H	-10.467190417	-6.934184512	-22.715296639
H	-11.334952961	-8.965258991	-24.463553368
H	-9.881888346	-8.323601014	-28.487017308
H	-11.163804781	-9.671053202	-26.852249622
H	-3.976571039	-8.996727846	-21.249672434
H	-4.694892579	-7.969149119	-23.347958205
H	-4.876566615	-9.712055783	-23.466238124
H	-7.141296567	-7.654277733	-23.044578578
H	-7.387523138	-9.402296232	-22.908890337
H	-6.879025425	-8.693268202	-24.455072094
H	-3.046607157	-5.179523004	-28.225559914
H	-6.086877750	-5.532516101	-27.930005746
H	-5.215718961	-5.503160062	-29.475934109
H	-5.979647714	-7.503116609	-26.480468349
H	-3.431474039	-7.241986836	-29.940682915
H	-5.158055620	-9.810329492	-26.049668728
H	-2.617429198	-9.549488916	-29.524107239
H	-3.463369391	-10.834149259	-27.549715640
H	-2.641102600	-13.527131629	-31.433451230
H	-7.215613247	-14.852970209	-22.567298306
H	-8.789448135	-11.346180131	-28.191232502
H	-4.278398722	-11.451133003	-35.090180478
N	-10.466167668	-8.838494996	-18.755875917
C	-10.118205072	-7.498698975	-19.194367007
H	-10.022162965	-6.809332004	-18.339385988
C	-11.212079405	-6.975162491	-20.120701212
H	-10.981147727	-5.943375506	-20.420197344
O	-11.330792049	-7.737522574	-21.332871884
O	-8.516718744	-9.813009622	-19.436208641
H	-11.430119724	-9.007648155	-18.474391141
H	-12.179669159	-6.965265313	-19.586419437
H	-9.149083354	-7.548857489	-19.704673616
H	-11.280764970	-8.699440252	-21.134538934
H	-7.000726994	-2.350806064	-25.167168066
C	-7.552380962	-3.132924826	-24.622740868
C	-6.547010095	-3.871511129	-23.756512029
O	-5.729416990	-4.679501108	-24.232794148
N	-6.570849054	-3.584921537	-22.436738278
C	-5.630934002	-4.177287955	-21.505242045
C	-6.145718009	-5.486160007	-20.909637975
O	-7.266472616	-5.537472326	-20.372990729
H	-5.472591991	-3.445790998	-20.701967970
N	-5.327549356	-6.550484017	-21.021840615
H	-8.876904260	-3.552333773	-26.272243274
H	-7.369797201	-4.456999066	-26.307752011
H	-8.318653853	-2.640082844	-24.007558581
H	-4.676127353	-4.325013266	-22.025695943
H	-4.425149908	-6.427408287	-21.474678511
H	-1.789517025	-5.874400026	-23.964633010
C	-2.664449940	-5.300128948	-24.289385998
C	-2.712922009	-5.311855973	-25.809405991
O	-1.687450792	-5.579786706	-26.469324382
H	-2.538709204	-4.262426704	-23.943170863
H	-3.582463745	-5.689840421	-23.835074311
H	-4.688599685	-4.770510285	-25.760016217
H	-7.282746904	-2.956336117	-22.076815867
H	-5.431884033	-8.037666003	-19.538077983
H	-6.812265553	-7.946850728	-20.643849577
O	-7.051209414	-13.594892527	-27.656480467
O	-3.429018144	-13.323417046	-26.462951763
H	-7.596500919	-13.839453216	-26.886999416

H	-2.840092038	-12.928538467	-27.128454843
H	-7.470423392	-10.533567587	-27.568866529

C3-ii

N	-6.901728673	-10.718203875	-28.690764186
C	-7.266539074	-11.047884902	-30.077725960
C	-8.608469234	-10.387427154	-30.448616243
O	-9.712449936	-10.845757717	-30.082720239
C	-7.311515050	-12.576607812	-30.266497376
C	-5.966769514	-13.243915085	-30.302356912
C	-5.109339993	-13.314253227	-31.382701664
N	-5.400646936	-13.965619068	-29.252827249
C	-4.239520378	-14.446971892	-29.695535319
N	-4.031115543	-14.068177998	-30.976926635
N	-8.496478320	-9.223243051	-31.123360610
C	-9.637159434	-8.393054100	-31.463222729
C	-9.168893476	-7.011459767	-31.939759065
O	-8.011961352	-6.608564443	-31.740630888
N	-10.111126916	-6.283511393	-32.579518797
C	-9.823183043	-5.014496956	-33.244852029
H	-10.721744966	-4.386782027	-33.242883981
H	-9.524393984	-5.204590017	-34.287201993
H	-6.634856002	-8.496379074	-35.345823014
C	-6.877447000	-8.731616796	-34.300433917
C	-5.636856971	-8.999301121	-33.465043106
O	-5.723215556	-9.211160070	-32.242050111
H	-7.531247001	-9.610727048	-34.252219017
N	-4.440790470	-8.994609563	-34.102507904
C	-3.260095078	-9.541344953	-33.464298800
C	-2.196802938	-8.536882993	-33.037759172
O	-1.045511882	-8.932060333	-32.779148041
C	-2.640367127	-10.634910654	-34.358073738
O	-3.516441243	-11.749906196	-34.538563323
N	-2.569018332	-7.252586756	-32.896423717
C	-1.620981985	-6.229227990	-32.487173958
H	-1.491963009	-6.154970008	-31.397096004
H	-1.985815006	-5.271092015	-32.873732006
H	-12.461799007	-11.734244001	-31.272652987
C	-12.324101981	-12.721649058	-31.732007020
C	-11.957925045	-13.787976885	-30.724054996
O	-12.529560620	-14.894818344	-30.761525483
H	-11.532447691	-12.622106945	-32.490900268
N	-11.037126324	-13.478862876	-29.768827292
C	-10.821918898	-14.384823158	-28.638754942
C	-10.975026130	-13.594969885	-27.344678067
O	-10.193089957	-12.681401043	-27.034376556
C	-9.520971522	-15.232964171	-28.676672714
C	-9.388550183	-15.937413679	-30.040873602
C	-9.534796144	-16.235416775	-27.514505723
C	-8.096747598	-16.735744615	-30.236880435
N	-12.018141699	-13.939689227	-26.555078840
C	-12.237610854	-13.280757985	-25.283068975
C	-11.225903891	-13.709613663	-24.212722097
O	-11.000217153	-14.905806289	-23.986120486
H	-13.214770078	-13.615247027	-24.911789009
N	-10.667655732	-12.693097973	-23.513844249
C	-9.900542068	-12.899494743	-22.305724430
C	-10.351806522	-11.850652241	-21.275030312
O	-10.908494391	-10.791105821	-21.612750594
C	-8.358709030	-12.772302040	-22.503815711

C	-7.768565688	-13.635169818	-23.565372278
C	-7.167465348	-13.281661242	-24.750801716
N	-7.674229139	-15.016483051	-23.515587640
C	-7.051303763	-15.448903558	-24.639042056
N	-6.723654218	-14.412334495	-25.410225435
N	-10.058850984	-12.152879902	-19.998350852
C	-10.362962976	-11.260640950	-18.897076065
C	-9.689314024	-9.902912034	-19.046613967
H	-9.989707000	-11.768595019	-17.999012966
C	-8.173754558	-4.096252948	-25.634240921
C	-8.844191962	-5.273296024	-24.993550020
C	-9.333688012	-5.385292998	-23.719740985
C	-9.257808675	-6.465419250	-25.692744495
C	-9.986226257	-7.265622935	-24.759558451
C	-9.102423925	-6.930558993	-27.012882639
N	-10.013422446	-6.582012220	-23.567150903
C	-10.546110525	-8.501925070	-25.110849684
C	-9.659547362	-8.158924054	-27.363448804
C	-10.374381632	-8.935872955	-26.424323880
C	-5.717887149	-7.891401018	-20.592887064
C	-5.031116358	-8.973711081	-21.434289122
C	-5.267787203	-8.882235678	-22.949180370
H	-5.388587619	-9.946796644	-21.060021525
C	-6.742020368	-8.895873928	-23.356093480
N	-3.887017919	-4.966205733	-26.375798853
C	-4.014021121	-4.861951011	-27.812786967
H	-4.117209928	-3.802590001	-28.100562024
C	-5.073066581	-5.811369759	-28.402826190
C	-4.552835555	-7.226714905	-28.292582964
C	-4.774630833	-8.000138600	-27.142411884
C	-3.689822717	-7.732319954	-29.278552017
C	-4.119441616	-9.223378163	-26.965730349
C	-3.039266008	-8.957510678	-29.110055594
C	-3.241788880	-9.701180805	-27.943368858
Cu	-5.745365733	-14.250788770	-27.213651566
H	-7.690946291	-10.908860110	-28.065464766
H	-6.488307902	-10.631497393	-30.729954847
H	-7.918228853	-13.013261573	-29.462774110
H	-7.832087920	-12.795321295	-31.211144757
H	-5.180836948	-12.911127302	-32.386646332
H	-3.557866490	-15.066913530	-29.125004165
H	-7.565142508	-8.936092551	-31.455698794
H	-10.256728827	-8.865815022	-32.243450585
H	-10.285418918	-8.267712591	-30.580626100
H	-11.007271706	-6.722879356	-32.774830459
H	-9.010267830	-4.502832600	-32.719539056
H	-7.416664378	-7.882960994	-33.860986797
H	-4.420154516	-8.823829344	-35.106291646
H	-3.610858118	-10.017878148	-32.533958280
H	-2.349558452	-10.198863936	-35.331296916
H	-1.738509416	-11.016480551	-33.868339431
H	-3.519218106	-6.984945415	-33.137903139
H	-0.640438445	-6.444702466	-32.930128819
H	-13.247691670	-13.026444557	-32.234819208
H	-10.555059561	-12.568811956	-29.792162740
H	-11.650587866	-15.107203267	-28.711402053
H	-8.657265780	-14.561287111	-28.536018554
H	-10.259168279	-16.601159660	-30.175876512
H	-9.456951665	-15.177679908	-30.834804271
H	-10.378641858	-16.937792979	-27.613211301
H	-9.625834167	-15.733706615	-26.540824720
H	-8.606446215	-16.821406670	-27.491800155

H	-8.001911413	-17.561670379	-29.517302362
H	-7.210058229	-16.094028988	-30.128849352
H	-8.069438853	-17.174400476	-31.245545361
H	-12.620659685	-14.712701087	-26.819826412
H	-12.251253443	-12.190922554	-25.416413157
H	-10.938217201	-11.730931456	-23.716374447
H	-10.132084886	-13.910004358	-21.937302209
H	-8.127122712	-11.730334777	-22.765510803
H	-7.878879129	-12.976778986	-21.533747419
H	-6.983000543	-12.303381802	-25.178879738
H	-6.869435078	-16.496950912	-24.850478633
H	-9.615741840	-13.041839138	-19.785160502
H	-11.453022479	-11.145710084	-18.789766877
H	-9.261865629	-4.700542760	-22.880053890
H	-8.555786555	-6.341449699	-27.752331486
H	-10.425323877	-6.953840975	-22.698764021
H	-11.101069085	-9.099160242	-24.385284021
H	-9.543645084	-8.534836105	-28.380179994
H	-10.794767951	-9.894570890	-26.732109473
H	-3.946366186	-8.946944684	-21.234988930
H	-4.788256544	-7.971615499	-23.344130325
H	-4.744742950	-9.726378479	-23.426278886
H	-7.275367037	-8.008370899	-22.985495280
H	-7.252882679	-9.782887903	-22.952886310
H	-6.849868275	-8.914176076	-24.449697212
H	-3.034546022	-5.155411933	-28.216892395
H	-6.032098089	-5.694602535	-27.875962751
H	-5.236069228	-5.541596582	-29.456954029
H	-5.453901334	-7.633497277	-26.369761798
H	-3.511892277	-7.144405169	-30.181939119
H	-4.302730636	-9.814883735	-26.068015276
H	-2.366360775	-9.326545185	-29.886428130
H	-2.732380432	-10.655662136	-27.802430023
H	-3.222323388	-14.311679304	-31.542541317
H	-8.032376071	-15.617007675	-22.776903499
H	-6.681488085	-9.723030246	-28.598463177
H	-4.280979523	-11.453681899	-35.065794551
N	-10.468791223	-8.838938414	-18.758624315
C	-10.118204997	-7.498698989	-19.194367015
H	-10.022162982	-6.809332010	-18.339386004
C	-11.208247202	-6.976722760	-20.125382884
H	-10.975457289	-5.946649987	-20.429438452
O	-11.323589560	-7.746243406	-21.333987346
O	-8.514530287	-9.812106894	-19.428116780
H	-11.434175023	-9.008680940	-18.482424082
H	-12.177772870	-6.963848032	-19.594741721
H	-9.147762953	-7.550531672	-19.702144769
H	-11.250262763	-8.705277023	-21.131618679
H	-7.000727006	-2.350806034	-25.167168001
C	-7.552381002	-3.132924928	-24.622741001
C	-6.547010004	-3.871511025	-23.756512001
O	-5.730006368	-4.679784955	-24.233134531
N	-6.570426817	-3.584261419	-22.436794007
C	-5.630934004	-4.177288003	-21.505242068
C	-6.145717956	-5.486159981	-20.909637944
O	-7.262656246	-5.534245068	-20.364393540
H	-5.472592016	-3.445790994	-20.701967963
N	-5.332467096	-6.552784139	-21.029165990
H	-8.889800742	-3.541527196	-26.264755220
H	-7.385003630	-4.450136697	-26.315752944
H	-8.315157207	-2.636965074	-24.005420038
H	-4.676232391	-4.324952737	-22.026006316

H	-4.433321394	-6.432215196	-21.489354515
H	-1.789516958	-5.874399932	-23.964633012
C	-2.664450094	-5.300129150	-24.289385970
C	-2.712921918	-5.311855919	-25.809406022
O	-1.687396945	-5.582668200	-26.468132198
H	-2.540056496	-4.262530629	-23.942510976
H	-3.582127638	-5.691467321	-23.835597291
H	-4.686933676	-4.766308134	-25.759586233
H	-7.284950093	-2.958995166	-22.076429238
H	-5.431883922	-8.037665985	-19.538077954
H	-6.811119746	-7.960797754	-20.650715583
O	-5.356163563	-16.128376268	-27.432597689
O	-5.374979892	-12.420268437	-26.817532937
H	-5.660382091	-11.858270138	-27.573013754
H	-5.379121559	-16.560134425	-26.561392624

References

(1) Gómez-Piñeiro, R. J.; Pantazis, D. A.; Orio, M. Comparison of Density Functional and Correlated Wave Function Methods for the Prediction of Cu(II) Hyperfine Coupling Constants. *ChemPhysChem* **2020**, *21*, 2667-2679.

(2) Yang, L.; Powell, D. R.; Houser, R. P. Structural variation in copper(I) complexes with pyridylmethylamide ligands: structural analysis with a new four-coordinate geometry index, tau4. *Dalton Trans.* **2007**, 955-964.

(3) Addison, A. W.; Rao, T. N.; Reedijk, J.; van Rijn, J.; Verschoor, G. C. Synthesis, structure, and spectroscopic properties of copper(II) compounds containing nitrogen–sulphur donor ligands; the crystal and molecular structure of aqua[1,7-bis(N-methylbenzimidazol-2′-yl)-2,6-dithiaheptane]copper(II) perchlorate. *J. Chem. Soc., Dalton Trans.* **1984**, 1349-1356.
